# Steric Effects Dictate the Formation of Terminal Arylborylene Complexes of Ruthenium from Dihydroboranes

**DOI:** 10.1002/chem.201902890

**Published:** 2019-09-17

**Authors:** Carsten Lenczyk, Dipak Kumar Roy, Jörn Nitsch, Krzysztof Radacki, Florian Rauch, Rian D. Dewhurst, F. Matthias Bickelhaupt, Todd B. Marder, Holger Braunschweig

**Affiliations:** ^1^ Institute for Inorganic Chemistry Julius-Maximilians-Universität Würzburg, Am Hubland, 97074, Würzburg (Germany), and Institute for Sustainable Chemistry & Catalysis with Boron, Julius-Maximilians-Universität Würzburg Am Hubland 97074 Würzburg Germany; ^2^ Department of Theoretical Chemistry and Amsterdam Center for Multiscale Modeling (ACMM) Vrije Universiteit Amsterdam De Boelelaan 1083, 1081 HV Amsterdam, (The Netherlands), and Institute for Molecules and Materials (IMM), Radboud University, Heyendaalseweg 135 6525 AJ Nijmegen The Netherlands; ^3^ Discipline of Chemistry Indian Institute of Technology Indore Khandwa Road Simrol, Indore 453552, M.P. India

**Keywords:** boranes, borohydrides, borylenes, steric effects, sigma boranes

## Abstract

The steric and electronic properties of aryl substituents in monoaryl borohydrides (Li[ArBH_3_]) and dihydroboranes were systematically varied and their reactions with [Ru(PCy_3_)_2_HCl(H_2_)] (Cy: cyclohexyl) were studied, resulting in bis(σ)‐borane or terminal borylene complexes of ruthenium. These variations allowed for the investigation of the factors involved in the activation of dihydroboranes in the synthesis of terminal borylene complexes. The complexes were studied by multinuclear NMR spectroscopy, mass spectrometry, X‐ray diffraction analysis, and density functional theory (DFT) calculations. The experimental and computational results suggest that the *ortho*‐substitution of the aryl groups is necessary for the formation of terminal borylene complexes.

## Graphical abstract

**Protect the boron**: Systematic investigation of the steric and electronic factors of dihydroboranes revealed that boranes bearing *ortho*‐substituted aryl groups are required for borylene formation on a ruthenium(II) center.

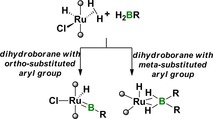

## Introduction

The dehydrogenative coupling of element hydrides for the formation of element–element bonds has attracted significant attention in recent years, and is becoming increasingly valuable in the synthesis of main‐group molecules and polymers.^1^ Although a large majority of work on dehydrocoupling is focused on the homonuclear dehydrocoupling of Si−H^2^ and P−H bonds,^3^ research interest in the heteronuclear dehydrocoupling of amine–boranes has soared over the last decade due to their growing importance as potential low‐weight hydrogen‐storage materials.^4^ In contrast, homodehydrocoupling reactions of B−H bonds were discovered in 1984 by Sneddon in the reactions of boranes and carboranes with PtBr_2_.^5^ This work later inspired us to develop a more atom‐efficient route to the synthesis of diboron(4) reagents of relevance to organic chemistry,^6^ leading to the establishment of the first synthetically viable dehydrogenative coupling of pinacolborane (HBPin, Pin=1,2‐O_2_C_2_Me_4_) and catecholborane (HBCat, Cat=1,2‐O_2_C_6_H_4_) to the corresponding diboranes(4) using either homo‐ or heterogeneous catalysts in 2011.^7^ Prior to this, Marder and co‐workers had observed the formation of small amounts of B_2_Pin_2_ from HBPin as a byproduct during the catalytic borylation of C−H bonds with HBPin.^8^ These results added another entry to the handful of methods for the selective construction of electron‐precise B−B bonds.^6^, ^9^

Although dihydrosilanes undergo dehydrocoupling to yield oligomeric or polymeric species,^2^ only one example of the dehydrogenation of dihydroboranes is known, whereby combination of a zerovalent platinum complex with the bulky duryldihydroborane (DurBH_2_, Dur=2,3,5,6‐Me_4_C_6_H) produced complex reactions leading to B−B single and double‐bond formation.^10^ Even though oxidative addition of the B−H bond of (RO)_2_BH to metal centers is facile,^11^ there are few examples of oxidative addition of the B−H bonds of dihydroboranes^12^ which in most cases result in relatively stable κ^2^‐bis(σ)‐borane complexes, preventing the oxidative addition of the B−H bonds.^13^ Nevertheless, the oxidative addition of both B−H bonds of a dihydroborane ([MesBH_2_]_2_) was achieved by Alcaraz, Sabo‐Etienne, and co‐workers in the synthesis of a borylene complex using the ruthenium–dihydrogen complex [Ru(PCy_3_)_2_HCl(H_2_)].^14^

The extensive work on both transition‐metal‐bound and metal‐free borylenes has suggested that addition of further (usually aliphatic) groups to boron‐bound aryl units is a prerequisite for borylene formation.13b, 15, 16 Thus, when equimolar quantities of Na[B^*m*^Fxyl_4_] (^*m*^Fxyl=3,5‐(CF_3_)_2_C_6_H_3_) were added to platinum boryl complexes of the form *trans*‐[PtBr(BBrAr)(PR_3_)_2_], with varying aryl substituents bound to boron, we encountered different outcomes of the reaction.^17^ The 4‐*tert*‐butylphenyl‐substituted complex formed a T‐shaped cationic boryl complex, whereas the duryl‐substituted complex underwent a formal boron‐to‐metal halide shift and formation of the corresponding cationic borylene complexes. In further work we showed that the use of boron substituents other than mesityl did not lead to analogous platinum borylene complexes but resulted instead in the abstraction of the bromo ligand at platinum.

Given the possibility of constructing boron–boron bonds through dehydrocoupling, we were interested in investigating whether the dehydrogenation process during the borylene formation from bis(σ‐B−H) ruthenium complexes14a, 14b suffers from limitations related to the substitution pattern of the boron‐bound aryl group of dihydroboranes, and if a chloride ligand at the metal center is necessary or not. Therefore, we synthesized a series of dihydroboranes and metal‐organic borohydrides, allowing us to embark on a systematic investigation of the steric and electronic factors required for borylene complex formation on ruthenium(II).

## Results and Discussion

### Dihydrido bis(σ)‐borane complexes: synthesis and characterization

Preliminary tests with a range of transition‐metal complexes indicated that [Ru(PCy_3_)_2_HCl(H_2_)] was the most promising for dehydrogenation reactions (discussed in greater detail below). To investigate the electronic and steric influence on the dehydrogenation of transition metal bis(σ‐B−H) complexes, we synthesized a range of dihydroboranes and aryl hydroborates with both electron‐rich and ‐poor aryl substituents. Li[DurBH_3_],13d (Dur=2,3,5,6‐Me_4_C_6_H) and Li[^*m*^FxylBH_3_]^18^ were synthesized according to the literature methods, whereas Li[^*o*^FxylBH_3_] (^*o*^Fxyl=2,6‐(CF_3_)_2_C_6_H_3_) was synthesized by modification of the synthesis of Li[^*m*^FxylBH_3_] developed by Wagner and co‐workers^18^ Based on protocols established by Pelter et al.^19^ Li[AnilBH_3_] (Anil=2,6‐Me_2_‐4‐(NMe_2_)C_6_H_2_) and Li[^*m*^XylBH_3_] (^*m*^Xyl=3,5‐Me_2_C_6_H_3_) were synthesized from their corresponding boronic esters. The dihydroboranes were generated from their aryl hydroborates Li[ArBH_3_] by treatment with trimethylsilyl chloride (TMSCl) and used in situ, except for DurBH_2_, which was isolated and used as a pure solid.

**Scheme 1:**
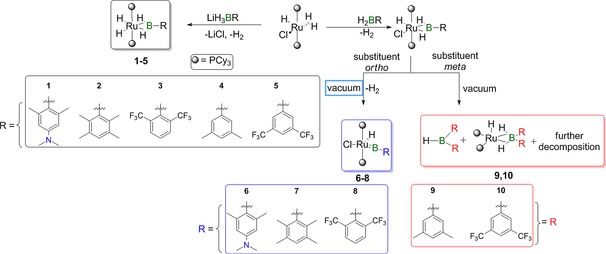
Synthesis of bis(σ)‐borane and borylene complexes.

The reactions of the lithium trihydroborates with [Ru(PCy_3_)_2_HCl(H_2_)] yielded the corresponding dihydrido bis(σ)‐borane complexes **1**–**5** in high yields (Scheme 1). The constitution of complexes **1**–**5** was verified by NMR spectroscopy, single‐crystal X‐ray diffraction analysis, and high‐resolution mass spectrometry. The high‐field region of the ^1^H NMR spectra of the bis(σ)‐borane complexes is diagnostic. A broad singlet and a triplet in a 1:1 ratio are assigned to the B−H and the Ru−H hydrogen nuclei, respectively. For all complexes, the broad singlet sharpened upon ^11^B decoupling, whereas the triplet collapsed to a sharp singlet upon ^31^P decoupling. The ^11^B NMR data for **1**–**5** show broad signals between *δ*=59 and 66 ppm (Table 1) that are in good agreement with previously described bis(σ)‐borane complexes (*δ*=54–70 ppm).^20^ Although complex **3** provided NMR data similar to those of **1**, **2**, **4**, and **5**, its NMR spectra revealed a further set of signals for each nucleus at room temperature. For example, the ^31^P{^1^H} NMR spectrum at room temperature exhibits a persistent set of two singlets at *δ*=79.8 and 81.2 ppm in approximately a 2:1 ratio indicating the presence of two phosphorus‐containing species. The hydride region of its ^1^H NMR spectrum displays three signals at *δ*=−17.13, −11.90, and −5.65 ppm. Despite the detection of two different sets of NMR signals for **3** in solution, mass spectrometry data supports its molecular formula. This suggested the presence of two isomers in solution; however, only one isomer was detected in the solid state. To confirm the presence of two isomers of **3** in solution we performed variable‐temperature (VT) NMR experiments, which showed that the ^1^H NMR signal at *δ*=−17.1 ppm split into two broad signals at −90 °C, whereas that at −5.7 split into a complex set of broad signals. All attempts to improve the resolution of these signals through measurement of 2D (^1^H−^1^H, ^1^H−^31^P) experiments and decoupling (^11^B, ^31^P) were unfortunately unsuccessful. The NMR results together with a literature precedent13c confirm the existence of two isomers in solution, such as dihydrido bis(σ)‐borane or σ‐borate species (see the Supporting Information).

**Table 1:** ^11^B NMR data and selected bond lengths and angles of **1**–**5**.

Complex	^11^B [ppm]	Ru−B [Å]	B−C1 [Å]	Ru‐B‐C1 [°]	P1‐Ru‐P2 [°]
**1**	59.9	1.964(3)	1.564(3)	177.7(2)	153.44(2)
**2**	57.7	1.953(4)	1.558(5)	177.6(2)	149.28(3)
**3**	48.2	1.950(3)	1.547(4)	176.8(2)	151.83(3)
**4**	63.1	1.953(2)	1.581(2)	176.8(2)	151.83(3)
**5**	66.4	1.944(3)	1.564(3)	170.3(2)	147.73(3)

**Figure 1:**
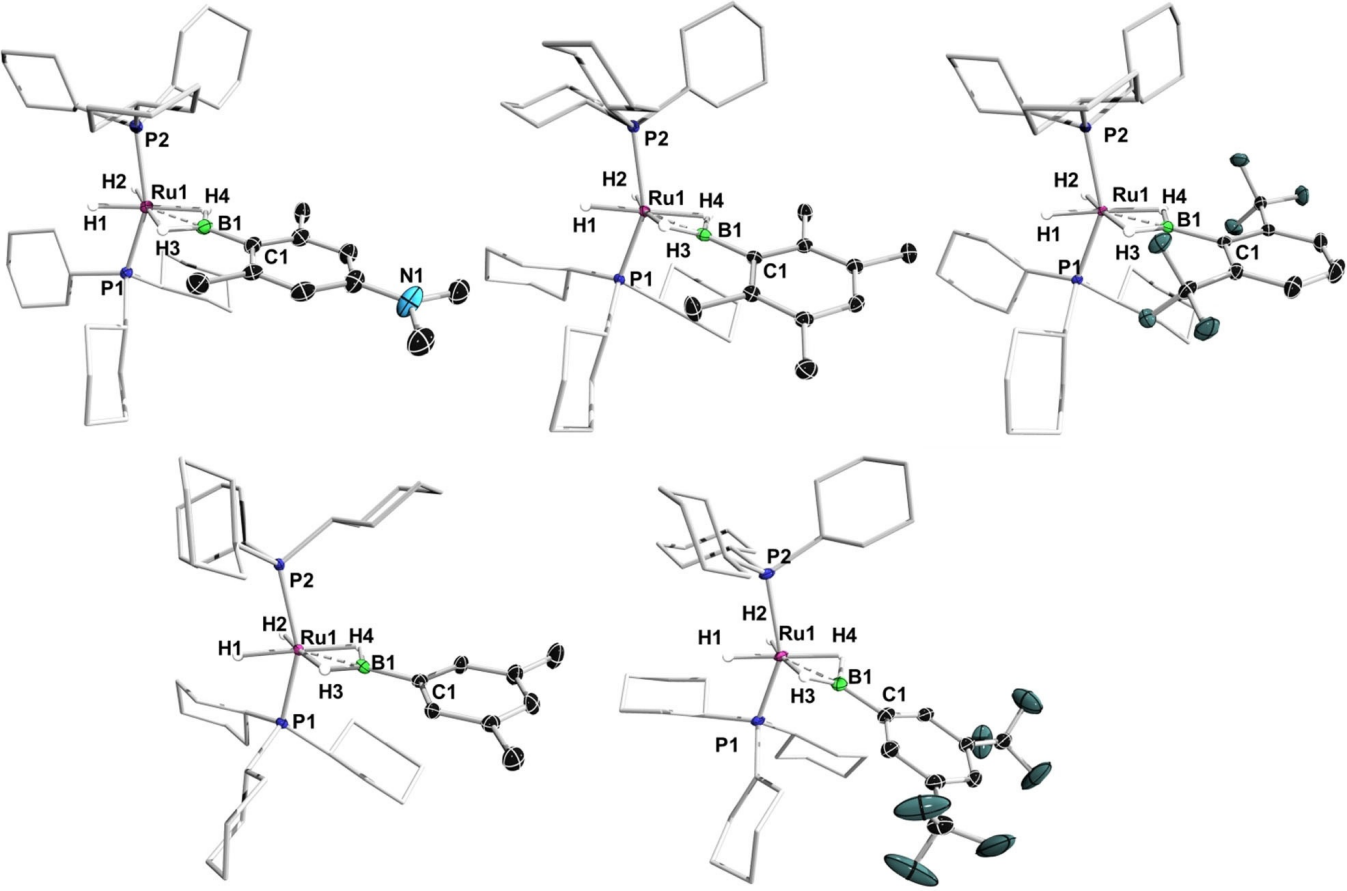
Crystallographically derived structures of **1**–**5**. Ellipsoids are shown at the 50 % probability level. Hydrogen atoms not bound to a metal center have been removed for clarity. Selected bond lengths [Å] and angles [°] for **1**: Ru1−B1 1.964(3), B1−C1 1.564(3); Ru1**‐**B1**‐**C1 177.7(2), P1**‐**Ru1**‐**P2 153.44(2), H1**‐**Ru1**‐**B1**‐**C1 6.63. For **2**: Ru1−B1 1.953(4), B1−C1 1.5585(5); Ru1**‐**B1**‐**C1 177.6(2), P1**‐**Ru1**‐**P2 149.28(3), H1**‐**Ru1**‐**B1**‐**C1 58.47. For **3**: Ru1−B1 1.953(2), B1−C1 1.581(2); Ru1**‐**B1**‐**C1 176.8(2), P1**‐**Ru1**‐**P2 151.83(3), H1**‐**Ru1**‐**B1**‐**C1 26.71. For **4**: Ru1−B1 1.950(3), B1−C1 1.547(4); Ru1**‐**B1**‐**C1 178.7(1), P1**‐**Ru1**‐**P2 147.24(2), H1**‐**Ru1**‐**B1**‐**C1 10.79. For **5**: Ru1−B1 1.944(3), B1−C1 1.564(3); Ru1**‐**B1**‐**C1 170.3(2), P1**‐**Ru1**‐**P2 147.73(3), H1**‐**Ru1**‐**B1**‐**C1 71.86.

Single crystals suitable for X‐ray structure analysis of **1**–**5** were obtained either by slow evaporation of pentane solutions, or by layering toluene solutions of the complexes with pentane. The solid‐state structures of **1**–**5** confirmed their formulation as being analogous to previously reported bis(σ)‐borane complexes.13c Only minor differences are observed in the solid‐state structures of **1**–**5**. In each structure, the Ru atom possesses a pseudo‐octahedral environment with the phosphine ligands in axial positions and four coplanar terminal and bridging hydrogen atoms occupying the equatorial coordination sites of the ruthenium (Figure 1).

**Figure 2:**
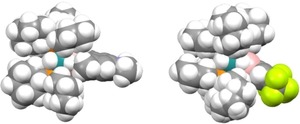
Space‐filling model of **1** and **5** showing the interactions between the aryl boron substituent and the phosphine ligands.

The interaction between the ruthenium and the boron atom is delineated by Ru−B distances (1.944(3)–1.964(3) Å) that are shorter than the sum of the respective covalent radii (2.09 Å, Table 1) and comparable to the ruthenium–boron distances of RuH_2_(η^2^:η^2^‐H_2_BR)(PCy_3_)_2_ (R=Mes, 1.938(4); R=*t*Bu 1.934(2); R=Ph, 1.923(8) Å). The significant bending of the P1‐Ru‐P2 angle (**1**: *d*_Ru−B_=1.964(3), ∡P1‐Ru‐P2=153.4°; **5**: *d*_Ru−B_=1.944(3), ∡P1‐Ru‐P2=147.7°; Table 1; Figure 2) is presumably a consequence of attractive dispersive interactions between C−H units of the PCy_3_ groups. The sum of the angles at boron in all of the complexes is 360°, which establishes the planar environment and sp^2^‐hybridization of the boron atom.

### Terminal borylene complexes: synthesis and characterization

In agreement with previous results,13c the ruthenium dihydrido bis(σ)‐borane complexes of this type could not be further dehydrogenated to afford borylene complexes, suggesting the vital importance of a non‐hydrogen ligand at the metal center. Given this reluctance, we performed the reaction of [Ru(PCy_3_)_2_(H_2_)HCl] with in situ‐generated dihydroboranes in order to prevent chloride/hydride exchange at Ru. To investigate the effect of the electronic and steric properties of dihydroboranes with respect to the formation of borylene complexes from their corresponding bis(σ)‐borane complexes, we prepared a range of dihydroboranes either in pure form or in situ from the reaction of monoaryl borohydrides (Li[ArBH_3_]) and TMSCl and treated them with a solution of [Ru(PCy_3_)_2_(H_2_)HCl]. In the case of dihydroboranes bearing *ortho*‐substituted aryl groups, namely DurBH_2_, ^*o*^FxylBH_2_, and AnilBH_2_, the ^11^B NMR spectra of the reaction mixture showed signals in the range *δ*=60–75 ppm, suggesting the formation of corresponding bis(σ)‐borane complexes. Subsequent evacuation of the reaction mixture and extraction with pentane allowed for the isolation of borylene complexes **6**–**8**. Complexes **6**–**8** revealed ^11^B NMR signals at *δ*=110.9, 110.1, and 98.8 ppm, respectively, comparable to those of reported ruthenium terminal borylene complexes.13b

**Figure 3:**
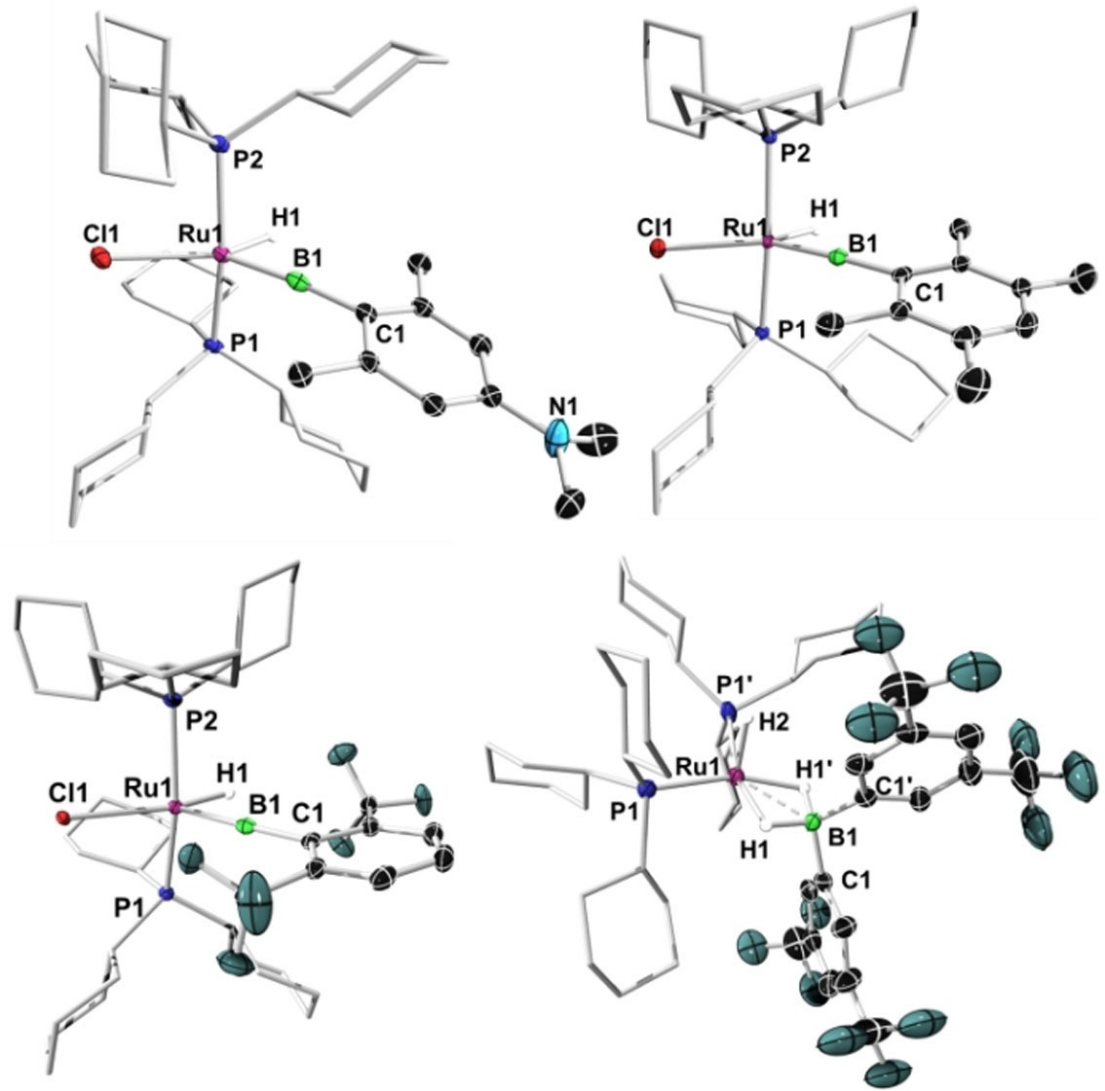
Crystallographically‐derived structures of **6**–**8** and **10**. Ellipsoids are shown at the 50 % probability level. Hydrogen atoms not bound to a metal center have been removed for clarity. Selected bond lengths [Å] and angles [°] for **6**: Ru1−B 1.807(3), B1−C1 1.532(4), Ru1−Cl1 2.4615(8); Cl1‐Ru1‐B1 136.00(9), Ru1‐B1‐C1 173.9(2), P1‐Ru‐P2 169.01(2). For **7**: Ru1−B 1.795(2), B1−C1 1.545(2), Ru1−Cl1 2.4521(7); Cl1‐Ru1‐B1 130.46(6), Ru1‐B1‐C1 177.0(1), P1‐Ru‐P2 168.44(2). For **8**: Ru1−B1 1.793(2), B1−C1 1.571(2), Ru1−Cl 2.4930(8); Cl1‐Ru1‐B1 128.28(7), Ru1‐B1‐C1 175.5(2), P1‐Ru1‐P2 161.74(2). For **10**: Ru1−B1 2.194(5), B1−C1 1.605(4), Ru1−H1 1.74(11), Ru1−H2 1.35(7); B1‐Ru1‐P1 126.62(3), P1‐Ru1‐P′ 106.77(5), C1‐B1‐C1′ 115.4(3).

In reactions using dihydroboranes with *meta*‐substitution only at the aryl moieties, namely ^*m*^FxylBH_2_ and ^*m*^XylBH_2_, we observed the formation of bis(σ)‐borane complexes, as indicated by ^11^B NMR spectroscopy. However, application of high vacuum or storage of the reaction mixture at room temperature led only to decomposition, the most prominent decomposition product being monoboranes of the form R_2_BH. In the case of the borane ^*m*^FXylBH_2_, the isolation of a borate complex [Ru(PCy_3_)_2_H(κ^2^‐H_2_B(^*m*^FXyl)_2_)] (**10**) was verified by means of NMR spectroscopy, single‐crystal X‐ray structure determination, and high‐resolution mass spectrometry. As in the case of bis(σ)‐borane complexes **1**–**5**, the comparison of the metric parameters of the borylene complexes **6**–**8** in the solid‐state structures as shown in Figure 3 reveals only minor differences, with complex **6** having a slightly longer Ru−B distance (1.807(3) Å) along with the smallest P1‐Ru‐P2 angle (169.01(3)°) distortion (Table 2). Complex **8** displays the largest distortion of the P‐Ru‐P angle.

**Table 2:** ^11^B NMR data and selected bond lengths and angles of **6**–**8**.

Complex	^11^B [ppm]	Ru−B [Å]	B−C1 [Å]	Ru‐B‐C1 [°]	P1‐Ru‐P2 [°]
**6**	110.9	1.807(3)	1.532(4)	173.9(2)	169.01(3)
**7**	110.1	1.795(2)	1.545(2)	177.0(1)	168.44(2)
**8**	98.8	1.793(2)	1.571(2)	175.5(2)	161.74(2)

**Figure 4:**
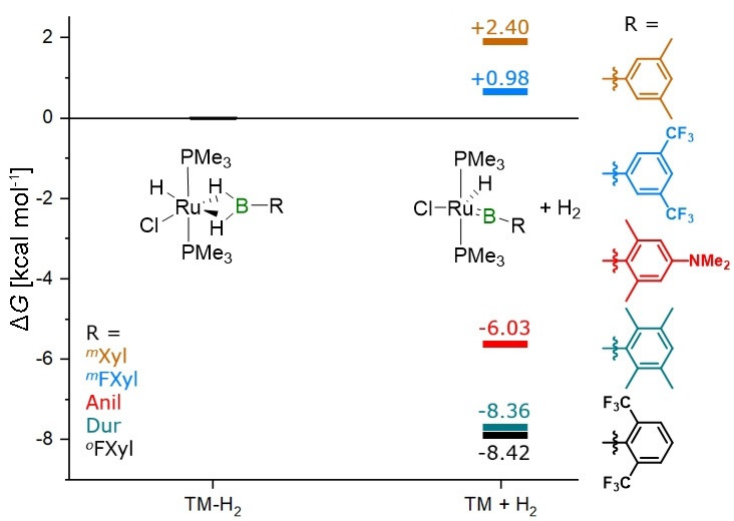
Calculated Δ*G* values (ZORA‐BLYP‐D3‐BJ/TZ2P) for the conversion of bis(σ)‐borane complexes to borylene complexes.

Given the isolation of different bis(σ)‐borane and borylene complexes, we were eager to shed some light on the factors involved in the conversion of bis(σ)‐borane complexes to borylene complexes with the help of DFT calculations at the ZORA‐BLYP‐D3‐BJ/TZ2P level of theory. We calculated Gibbs free energies (Δ*G*) for the transformation of bis(σ)‐borane complexes to the borylene complexes along with liberation of hydrogen (TM−H_2_→TM+H_2_; Figure 4) and found that the steric properties of the boranes play a crucial role, with the electronic properties having only a minor impact on the overall process.

Boranes containing *ortho*‐substituted aryl groups tend to destabilize the bis(σ)‐borane complexes and favor the formation of borylene complexes by dissociation of dihydrogen, resulting in an exergonic reaction (Δ*G* between −6.03 and −8.42 kcal mol^−1^). For boranes with aryl groups with unsubstituted 2,6 positions we observed positive Δ*G* values suggesting an endergonic reaction (Δ*G* between +0.98 and +2.40 kcal mol^−1^). Even though we obtained negative Δ*G* values for all of the *ortho*‐substituted boranes, [^*o*^FxylBH_2_] has the most negative one. Conversion of the bis(σ)‐borane complex involving *meta*‐substituted boranes is endergonic and electronic effects of the boranes are subtle, accounting only for a 1.42 kcal mol^−1^ difference in the calculated Δ*G* for the formation of the borylene complex. Our previous work on platinum borylene complexes also suggested that *ortho*‐substitution of the boron‐bound aryl group is a prerequisite for a halide shift and borylene formation.17d

**Scheme 2:**
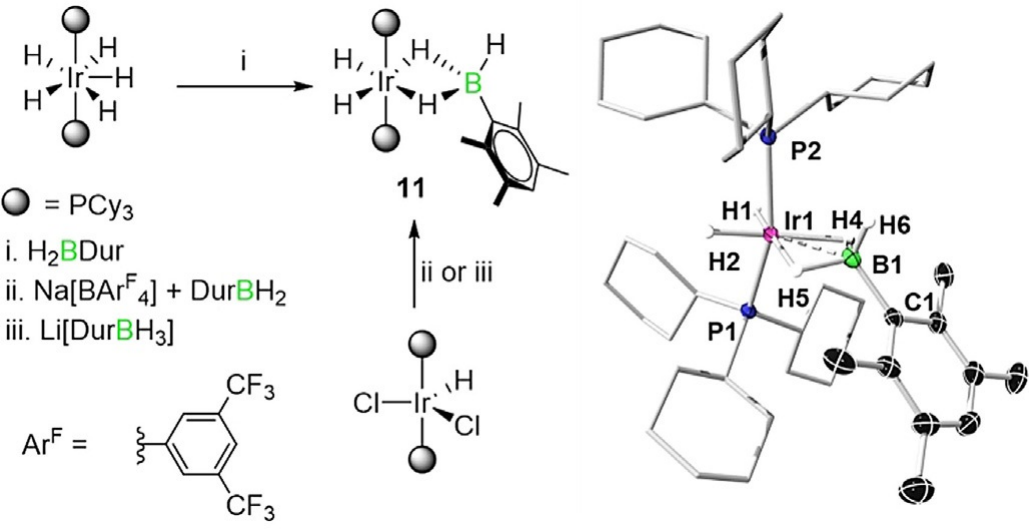
Synthesis and solid‐state structure of iridium borate complex **11**.

In order to evaluate the suitability of [Ru(PCy_3_)_2_HCl(H_2_)] for dihydroborane dehydrogenation and borylene formation relative to other promising late‐transition‐metal complexes, we tested three different rhodium and iridium complexes with duryl‐substituted borane precursors. The treatment of either [Rh(PCy_3_)_2_Cl(H_2_)] or [Rh(PCy_3_)_2_Cl]_2_ with either [DurBH_2_]_2_ or Li[DurBH_3_] led only to decomposition of the starting materials. With [Ir(PCy_3_)_2_H_5_] and DurBH_2_ we isolated the iridium borate complex **11** (Scheme 2) in 64 % yield; however, application of high vacuum to either the reaction mixture or pure **11** provided no sign of conversion to bis(σ)‐borane or borylene complexes. Treatment of [Ir(PCy_3_)_2_(H)Cl_2_] with either Na[B^*m*^Fxyl_4_]/DurBH_2_ or Li[DurBH_3_] also gave **11** in moderate yields. The ^11^B NMR spectrum of **11** showed a broad signal at *δ*=21.1 ppm and the ^1^H NMR spectrum revealed signals at *δ*=−19.83 and −6.37 ppm for terminal (Ir‐bound) and bridging hydrides, respectively, along with a broad peak at *δ*=8.47 ppm for the terminal B−H. The solid‐state structure of **11**, as shown in Scheme 2, exhibits a tetrahedral arrangement of the boron center, and the Ir−B distance (2.237(3) Å) is similar to other related iridium dihydroborate systems, such as [(*t*BuPCP)IrH(κ^2^‐H_2_BHDur)],13d (2.283(2); *t*BuPCP: κ^3^‐C_6_H_3_‐1,3‐[CH_2_P*t*Bu_2_]_2_) [(Cy_3_P)_2_Ir(H)_2_(κ^2^‐H_2_BH⋅NMe_3_)][B^*m*^Fxyl_4_],^20^ (2.207(7)), and [(SIMes)_2_Ir(H)_2_(κ^2^‐H_2_BH⋅NMe_2_H)][B^*m*^Fxyl_4_]^21^ (2.21(4) Å; SIMes: 1,3‐bis(2,4,6‐trimethylphenyl)imidazolidin‐2‐ylidene).

## Conclusions

A series of monoaryl borohydrides and dihydroboranes were prepared and treated with [Ru(PCy_3_)_2_HCl(H_2_)], leading to either bis(σ)‐borane or borylene complexes. By systematic variation of steric and electronic factors, trends are observed in the coordination behavior of the boron‐containing precursors, the most striking being: (a) the chloride/hydride exchange process at Ru (through the use of Li[ArBH_3_]) prevents further hydrogen loss and borylene generation; and (b) boranes bearing *ortho*‐substituted aryl groups are seemingly required to form borylenes. Results of DFT calculations were in accordance with the fact that the formation of borylenes from bis(σ)‐borane complexes is only observed experimentally when boranes with *ortho*‐substituted aryl groups were used. Experimental findings further demonstrated the necessity of the chloro ligand (or at least a ligand that is not a hydride) in the bis(σ)‐borane complex as a second criterion for the formation of borylene complexes.^22^

## Conflict of interest

The authors declare no conflict of interest.

## Supporting information

As a service to our authors and readers, this journal provides supporting information supplied by the authors. Such materials are peer reviewed and may be re‐organized for online delivery, but are not copy‐edited or typeset. Technical support issues arising from supporting information (other than missing files) should be addressed to the authors.

Supplementary
