# Supplementary material for: Steric Effects Dictate the Formation of Terminal Arylborylene Complexes of Ruthenium from Dihydroboranes
Source: Chemistry. 2019 Sep 17;25(59):13566–71. doi: 10.1002/chem.201902890 (PMC7079023; doi:10.1002/chem.201902890)
Supplement: Supplementary file 1 — Supplementary [file CHEM-25-13566-s001.pdf]

CHEMISTRY
A European Journal

Supporting Information

Steric Effects Dictate the Formation of Terminal Arylborylene Complexes of Ruthenium from Dihydroboranes

Carsten Lenczyk,[a] Dipak Kumar Roy,[a, c] Jörn Nitsch,[a] Krzysztof Radacki,[a] Florian Rauch,[a] Rian D. Dewhurst,[a] F. Matthias Bickelhaupt,[b] Todd B. Marder,[a] and Holger Braunschweig*[a]

chem_201902890_sm_miscellaneous_information.pdf

Materials and Methods

General considerations: all reactions were performed under an atmosphere of dry argon (argon 5.0) using standard Schlenk or glovebox techniques. All solvents were purified by distillation using the appropriate drying agents, deoxygenated using three freeze-pump-thaw cycles and stored over molecular sieves under dry argon prior to use. Deuterated solvents used for NMR spectroscopy were purchased from Cambridge Isotope Laboratories, deoxygenated by freeze-pump-thaw cycles and dried under an argon atmosphere over molecular sieves. ^{1}H, ^{11}B and $^{13}C\{^{1}H\}$ $^{31}P\{^{1}H\}$ NMR spectroscopy data were obtained at ambient temperature using either a *Bruker DRX-400* (operating at 400.1 MHz for ^{1}H, 128.4 MHz for ^{11}B, 100.6 MHz for $^{13}C\{^{1}H\}$ and 162 MHz for $^{31}P\{^{1}H\}$) or a *Bruker Avance 500* NMR spectrometer (operating at 500.1 MHz for ^{1}H, 160.5 MHz for ^{11}B, 125.8 MHz for $^{13}C\{^{1}H\}$ and 202 MHz for $^{31}P\{^{1}H\}$). ^{1}H NMR spectra were referenced via residual proton resonances of C_6D_6 (^{1}H, 7.16 ppm). $^{13}C\{^{1}H\}$ spectra were referenced to C_6D_6 (^{13}C, 128.06 ppm). High-resolution mass spectrometry was performed using a Thermo Scientific Exactive Plus spectrometer in LIFDI mode.

Materials: TMSCl was purchased from Sigma-Aldrich and distilled under argon. [PCy_3],[1] 4-bromo-*N,N*,3,5-tetramethylaniline,[2] [^{o}FXyl-I][3] [$DurBH_2$]$_2$[4] Li[$DurBH_3$],[4] Li[^{m}FXylBH$_3$][5] and the precursors [RuCl(μ-Cl)(η^3:η^3-$C_{10}H_{16}$)]$_2$,[6] [Ru(PCy_3)$_2$(H_2)HCl],[7] [RhCl(PCy_3)$_2$]$_2$,[8] [Rh(PCy_3)$_2$$H_2$Cl],[9] [Ir($PCy_3$)$H_5$)][10] and [Ir($PCy_3$)$Cl_2$H][10] were synthesized according to known methods.

Lithium (3,5-dimethylphenyl)borate

Li[^{m}XylBH$_3$]·1.5 THF

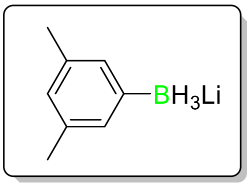

^{1}H NMR (400.1 MHz, C_6D_6 / Et_2O, 296 K): δ (ppm) = 1.62 (q, 3H, $^{1}J_{H\text{-}B}$ = 75.4 Hz, B-*H*), 2.38 (s, 6H, CH_3, CH_3^{meta}), 6.19 (s, 1H, CH, CH^{para}), 6.61 (s, 2H, CH, CH^{ortho}).

^{11}B NMR (128.4 MHz, C_6D_6 / Et_2O, 296 K): δ (ppm) = –25.1 (q, $^{1}J_{B\text{-}H}$ = 75.4 Hz, *B*H_3).

^{13}C{^{1}H} NMR (100.6 MHz, C_6D_6 / Et_2O, 296 K): δ (ppm) = 21.8 (CH_3, *C*H_3^{meta}), 126.0 (CH, *C*H^{para}), 134.0 (CH, *C*H^{ortho}), 135.6 (C_q, *C*CH_3) 149.9 (C_q, C_q-B).

ESI-MS (Toluene) positive *m/z*: $[M\text{-}H\text{+}THF]^+$ 190.1343 (calculated: 190.1529) $[M\text{-}BH_2Li]^+$ 106.0770 (calculated: 106.0783)

Lithium *N,N*,3,5-tetramethylaniline-4-borate

Li[AnilBH$_3$]·1.5 THF

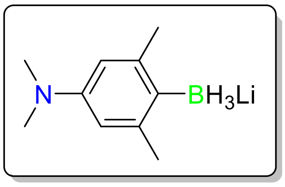

^{1}H NMR (400.1 MHz, C_6D_6/THF, 296 K): δ (ppm) = 1.43 (q, $^{1}J_{B\text{-}H}$ = 76.2 Hz, 3H, B-*H*), 2.78 (s, 6H, CH_3, CH_3^{ortho}), 2.80 (s, 6H, CH_3, NCH_3), 6.80 (s, 2H, CH, CH^{ortho}).

^{11}B NMR (128.4 MHz, C_6D_6 /THF, 296 K): δ (ppm) = –30.8 (q, $^{1}J_{B\text{-}H}$ = 76.2 Hz, *B*H_3).

^{13}C{^{1}H} NMR (100.6 MHz, C_6D_6/THF, 296 K): δ (ppm) = 25.6 (CH_3, *C*H_3^{ortho}), 25.6 (CH_2, THF), 41.8 (CH_3, *C*H_3^{ortho}), 68.1 (CH_2, THF), 113.4 (CH, *C*H^{meta}), 138.6 (C_q, C_q-B), 142.5 (C_q), 148.9 (C_q).

ESI-MS (Toluene) positive *m/z*: $[M\text{-}H\text{+}THF]^+$ 233.1929 (calculated: 233.1951) $[M\text{-}Li\text{-}H]^+$ 161.1359 (calculated: 161.1376).

Lithium 1,3-bis(trifluoromethyl)phenyl-2-borate

Li[^{o}FXylBH$_3$]·1.5 Et_2O

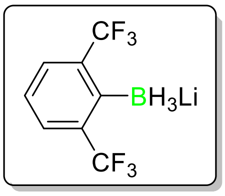

^{1}H NMR (400.1 MHz, C_6D_6, 296 K): δ (ppm) = 1.72 (q, $^{1}J_{B\text{-}H}$ = 79.8 Hz, 3H, B-*H*), 6.82 (t, $^{3}J_{H\text{-}H}$ = 8.0 Hz, CH, C*H*^{para}), 7.75 (d, $^{3}J_{H\text{-}H}$ = 8.0 Hz, CH, C*H*^{meta}).
^{11}B NMR (128.4 MHz, C_6D_6, 296 K): δ (ppm) = –30.7 (q, $^{1}J_{B\text{-}H}$ = 79.8 Hz, *B*H_3).
^{13}C{^{1}H} NMR (100.6 MHz, C_6D_6, 296 K): δ (ppm) = 123.5 (CH, *C*H^{para}), 126.8 (q, $^{2}J_{C\text{-}F}$ = 274.5 Hz, C_q, *C*F_3), 128.5 (CH, *C*H^{meta}) 136.3 (q, $^{2}J_{C\text{-}F}$ = 27.5 Hz, C_q, *C*CF_3), 156.5 (C_q, *C*$_q$-B).
^{19}F NMR (376.5 MHz, C_6D_6, 296 K): δ (ppm) = –58.4 (s).
ESI-MS (Toluene) positive *m/z*: $[M\text{-}2H\text{-}Li\text{+}THF]^+$ 297.0861 (calculated: 297.0885).

Synthetic protocol for the synthesis of ruthenium dihydrido bis(σ-borane) complexes [Ru(PCy_3)$_2$$H_2$($\sigma^2$-$H_2$BR)]

To [Ru(PCy_3)$_2$(H_2)HCl] (120 mg, 0.17 mmol, 1.0 equiv) the corresponding metal organic borohydride was added dropwise as an ethereal solution (2 mL). *In the case of metal organic borohydrides with unknown amounts of coordinating solvent, the addition was carried out by stepwise addition and subsequent monitoring by NMR spectroscopy.*
A spontaneous gas evolution was observed and the solution was stirred at room temperature for 5 min. The previously orange solution turned yellow and after removal of the solvent the complexes were suspended in toluene and filtered over celite. The solvent was then removed under vacuum, leading to the desired complexes.
Crystals were either obtained by slow evaporation of pentane solutions, or layering a toluene solution of the complexes with pentane.

[Ru(PCy_3)$_2$$H_2$($\sigma^2$-$H_2$BAnil)] (1)

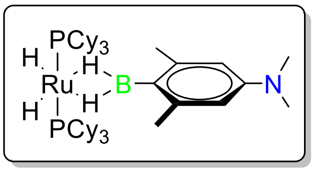

Li[AnilBH_3]·1.5 THF (57.15 mg, 0.204 mmol, 1.2 equiv)
yield: 52% (72 mg, 0.09 mmol)

^{1}H NMR (400.1 MHz, C_6D_6, 296 K): δ (ppm) = –11.35 (m, 2H, Ru-*H*), –6.04 (br m, 2H, σ^2-B-*H*), 1.13-1.38 (m, 18H, *Cy*), 1.55-1.73 (m, 18H, *Cy*), 1.73-1.94 (m, 18H, *Cy*), 2.20 (m, 12H, *Cy*), 2.47 (s, 6H, CH_3, CH_3^{ortho}), 3.06 (s, 6H, CH_3, NCH_3).
^{11}B NMR (128.4 MHz, C_6D_6, 296 K): δ (ppm) = 66.4 (br. s).
^{13}C{^{1}H} NMR (100.6 MHz, C_6D_6, 296 K): δ (ppm) = 24.3 (CH_3, N*C*H_3), 27.3 (CH_2, *CH_2*-Cy), 28.3 (CH_2, *CH_2*-Cy), 30.9 (CH_2, *CH_2*-Cy), 39.5 (CH_2, *CH_2*-Cy), 39.5 (CH_3, *C*H_3^{ortho}-Aniline), 112.0 (CH, *C*H^{meta}-Aniline), 130.0 (C_q, C_q-B), 146,7 (C_q), 151.6 (C_q).
^{31}P{^{1}H} NMR (161.9 MHz, C_6D_6, 296 K): δ (ppm) = 83.2 (s).
ESI-MS (Toluene) positiv *m/z,* [M-4H] 821.4898 (calculated: 821.4893), [M-H+Cl] 859.4665 (calculated: 859.4626), [M-3H+Cl] 857.4667 (calculated: 857.4669).

[Ru(PCy$_3$)$_2$H$_2$(σ^2-H$_2$BDur)] (2)

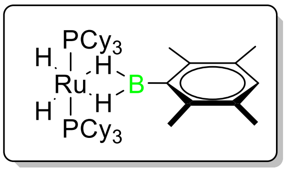

Li[DurBH$_3$]·1.5 THF (54 mg, 0.204 mmol, 1.2 equiv)
yield: 87% (120 mg, 0.15 mmol)

^{1}H NMR (400.1 MHz, C_6D_6, 296 K): δ (ppm) = –11.16 (t, $^{2}J_{P\text{-}H}$ = 25.8 Hz, 2H, Ru-*H*), –5.95 (br s, 2H, σ^2-B-*H*), 1.16-1.34 (m, 18H, *Cy*), 1.53-1.73 (m, 18H, *Cy*), 1.73-1.95 (m, 18H, *Cy*), 2.15 (s, 6H, CH_3, *C*H_3-Dur), 2.16-2.25 (m, 12H, *Cy*), 2.95 (s, 6H, CH_3, *C*H_3-Dur), 6.91 (s, 1H, CH^{para}-Dur).
^{11}B NMR (128.4 MHz, C_6D_6, 296 K): δ (ppm) = 57.7 (br. s).
^{13}C{^{1}H} NMR (100.6 MHz, C_6D_6, 296 K): δ (ppm) = 14.2 (CH_3, CH_3-Dur), 19.1 (CH_3, *C*H_3-Dur) 20.7 (CH_2, *CH_2*-Cy), 27.3 (CH_2, *CH_2*-Cy), 28.2 (CH_2, *CH_2*-Cy), 30.9 (CH_2, *CH_2*-Cy), 39.4 (CH, *CH*-Cy), 128.6, 133.5 (C_q, *C*$_q$-Dur), 134.6 (CH, *C*H^{para}-Dur),140.8 (C_q, *C*$_q$-Dur), 141.9 (C_q, *C_q*-B).
^{31}P{^{1}H} NMR (161.9 MHz, C_6D_6, 296 K): δ (ppm) = 83.7 (s).
ESI-MS (Toluene) positive *m/z*, [M] 810.4866 (calculated: 810.5107), [M-4H] 806.4794 (calculated: 806.4794).

[Ru(PCy$_3$)$_2$H$_2$(σ^2-H$_2$B^{o}FXyl)] (3)

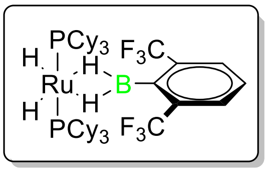

Li[^{o}FXylBH$_3$] ·1.5 Et$_2$O (70 mg, 0.204 mmol, 1.2 equiv)
yield: 36% (55 mg, 0.06 mmol)

The product is highly soluble in pentane, whereby an additional 40 mg could be obtained by fractional crystallization. However, this sample is contaminated by grease. Upon dissolving the crystals two sets of signals were obtained in the ^{1}H, ^{11}B, ^{19}F, and ^{31}P{1H} NMR spectra.

^{1}H NMR (400.1 MHz, C_6D_6, 340 K): δ (ppm) = –17.13 (br. s, Ru-*H*), –11.90 (br, s, Ru-H), –5.65 (br, s, 2H, σ^2-B-*H*), 1.19-1.373 (m, 1H, *Cy*), 1.56-1.90 (m, 36H, *Cy*), 2.11-2.2 (m, 12H, *Cy*), 6.77 (t, $^{3}J_{H-H}$ = 7.85 Hz, CH, CH^{para}), 7.52 (d, $^{3}J_{H-H}$ = 7.85 Hz, 2H, CH^{meta}).

^{11}B NMR (128.5 MHz, C_6D_6, 296/340 K): δ (ppm) = 16.5 (br. s), 48.2 (br.s).

^{13}C{^{1}H} NMR (125.8 MHz, C_6D_6, 296 K): δ (ppm) = 27.2 (CH_2, *CH_2*-Cy), 28.3 (CH_2, *CH_2*-Cy), 30.8 (CH_2, *CH_2*-Cy), 39.4 (CH, *CH*-Cy), 125.3 (d, $^{1}J_{C-F}$ = 275.7 Hz, C_q, *CF_3*), 127.6 (CH, *C*H^{para}-^{o}FXyl), 128.6 (br. s, CH, CH^{meta}-^{o}FXyl), 129.0 (br s, C_q, C_q-B), 136.7 (q, $^{2}J_{C-F}$ = 29.70 Hz, C_q, *C*CF_3-^{o}FXyl).

^{19}F NMR (125.8 MHz, C_6D_6, 296 K): δ (ppm) = -54.6, -56.1.

^{31}P{^{1}H} NMR (202.5 MHz, C_6D_6, 296 K): δ (ppm) = 79.8, 81.9.

ESI-MS (Toluene) positive *m/z*, [M] 890.3988 (calculated: 890.3994), [M-H +Li] 896.4398 (calculated: 896.4310) [M-4H] 886.3929 (calculated: 886.3915).

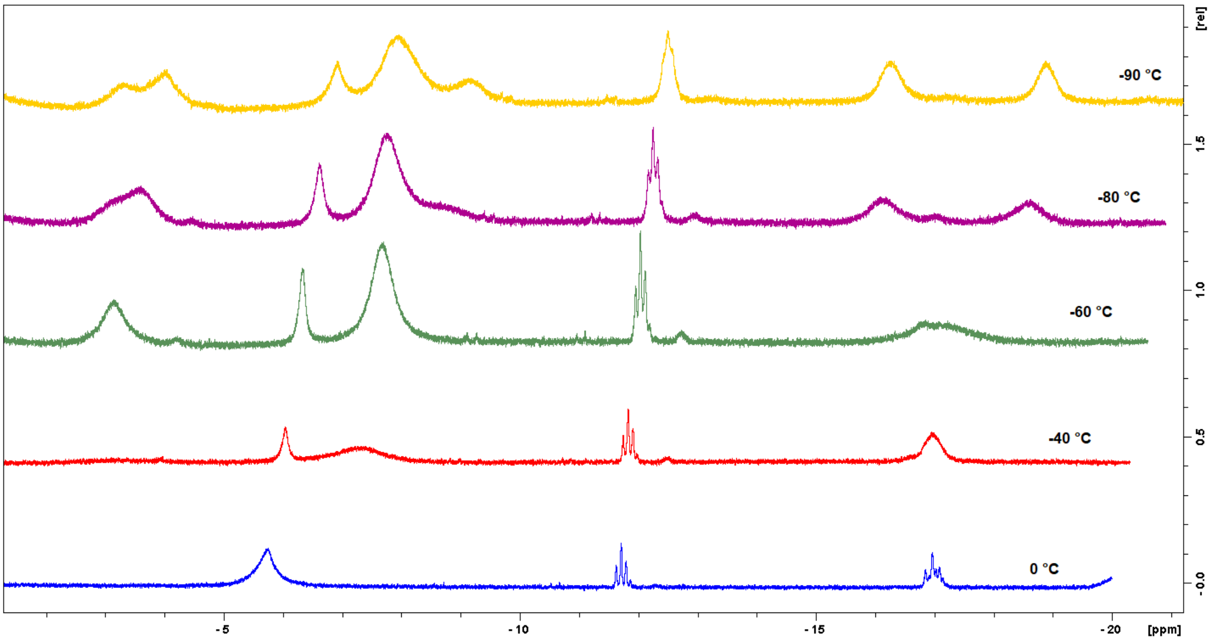

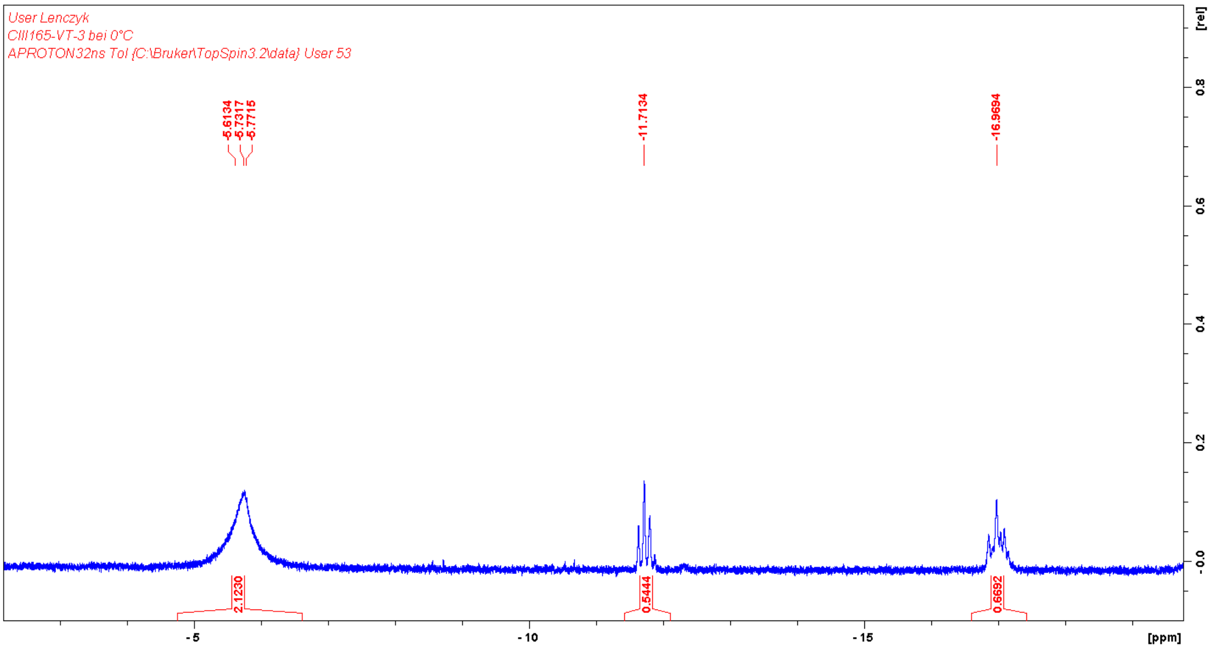

Figure S1. Variable-temperature ^{1}H NMR plot (top). Integration of hydride region at 0 °C of **3** (bottom).

[Ru(PCy$_3$)$_2$H$_2$(σ^2-H$_2$B^{m}Xyl)] (4)

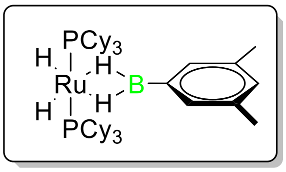

Li[^{m}XylBH$_3$] ·1.5 THF (48.4 mg, 0.204 mmol, 1.2 equiv)

yield: 61% (82 mg, 0.10 mmol)

^{1}H NMR (400.1 MHz, C_6D_6, 296 K): δ (ppm) = –11.27 (t, $^{2}J_{P\text{-}H}$ = 25.9 Hz 2H, Ru-*H*), –5.7 (br s, 2H, σ^2-B-*H*), 1.17-1.33 (m, 18H, *Cy*), 1.56-1.71 (m, 18H, *Cy*), 1.75-1.92 (m, 18H, *Cy*), 2.19 (m, 12H, *Cy*), 2.23 (s, 6H, CH$_3$, C*H*$_3^{meta}$-^{m}Xyl), 6.86 (s, 1H, C*H*^{para}-^{m}Xyl), 7.75 (s, 2H, C*H*^{ortho}-^{m}Xyl)

^{11}B NMR (128.4 MHz, C_6D_6, 296 K): δ (ppm) = 63.1 (br. s).

^{13}C{^{1}H} NMR (100.6 MHz, C_6D_6, 296 K): δ (ppm) = 21.3 (CH$_3$, *C*H$_3^{meta}$), 27.3 (CH$_2$, *CH$_2$*-Cy), 28.2 (CH$_2$, *CH$_2$*-Cy), 30.9 (CH$_2$, *CH$_2$*-Cy), 39.1 (CH, *CH*-Cy), 132.1 (CH, *C*H^{para}-^{m}Xyl), 134.2 (CH, CH^{ortho}-^{m}Xyl), 137.1 (C$_q$, *C*$_q$-^{m}Xyl), 145.1 (C$_q$, *C*$_q$-B).

^{31}P{^{1}H} NMR (161.9 MHz, C_6D_6, 296 K): δ (ppm) = 81.4 (s).

ESI-MS (Toluene) positive *m/z*, [M-3H] 779.4500 (calculated: 779.4559) [M-4H] 778.4467 (calculated: 778.4481), [M-3H+Cl] 814.4235 (calculated: 814.4247).

[Ru(PCy$_3$)$_2$H$_2$(σ^2-H$_2$B^{m}FXyl)] (5)

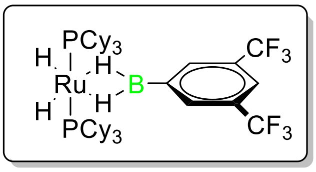

Li[^{m}FXylBH$_3$] ·1.5 Et$_2$O (70 mg, 0.204 mmol, 1.2 equiv)
yield: 62% (94 mg, 0.11 mmol)

^{1}H NMR (400.1 MHz, C_6D_6, 296 K): δ (ppm) = –11.61 (m, 2H, Ru-*H*), –5.31 (br. s, 2H, σ^2-B-*H*), 1.14-1.23 (m, 18H, *Cy*), 1.46-1.58 (m, 12H, *Cy*), 1.58-1.66 (m, 6H, *Cy*), 1.72-1.84 (m, 18H, *Cy*), 2.06-2.15 (m, 12H, *Cy*), 7.78 (s, 1H, CH^{para}), 8.48 (s, 2H, CH^{ortho})
^{11}B NMR (128.4 MHz, C_6D_6, 296 K): δ (ppm) = 66.4 (br. s).
^{13}C{^{1}H} NMR (100.6 MHz, C_6D_6, 296 K): δ (ppm) = 27.1 (CH_2, *CH$_2$*-Cy), 28.1 (CH_2, *CH$_2$*-Cy), 30.9 (CH_2, *CH$_2$*-Cy), 38.9 (CH, *CH*-Cy), 122.9 (CH, *C*H^{para}-^{m}FXyl), 124.2 (q, $^{1}J_{C\text{-}F}$ = 272.9 Hz, C_q, *C*F$_3$), 131.5 (q, $^{2}J_{C\text{-}F}$ = 32.7 Hz, C_q, *C*CF$_3$), 135.3 (CH, *C*H^{ortho}-^{m}FXyl), 147.85 (C_q, *C$_q$*-B).
^{19}F NMR (376.5 MHz, C_6D_6, 296 K): δ (ppm) = –62.6 (s).
^{31}P{^{1}H} NMR (161.9 MHz, C_6D_6, 296 K): δ (ppm) = 81.2 (s).
ESI-MS (Toluene) positive *m/z*, [M+H] 891.4399 (calculated: 891.4306), [M-BH$_3$+Li] 884.4168 (calculated: 884.4139).

Synthetic protocol for the synthesis of borylene complexes [Ru(PCy$_3$)$_2$HCl(BR)] (6-8)

In reactions involving metal organic borohydrides, the conversion of the borates to dihydroboranes was performed in Et$_2$O with approximately 2 equiv TMSCl (c = 0.086 mmol/mL). Within a few seconds the clear colorless solution turns into a white suspension. After 20 min all volatiles were quickly removed in vacuum (30 mmHg) resulting in the formation of a colorless oil or white solid. The reaction mixture was dissolved in Et$_2$O and used in the reaction. In the case of DurBH$_2$ the dihydroboranes was added separately to the metal precursor.

[Ru(PCy$_3$)$_2$(H$_2$)HCl] (120 mg, 0.17 mmol, 1 equiv) was dissolved in toluene. To the orange ethereal solution, a freshly prepared solution of the dihydroborane (1.5 equiv) was added. *In the case of metal organic borohydrides with unknown amounts of coordinating solvent, the addition was carried out by stepwise addition and subsequent monitoring by NMR spectroscopy.*
A spontaneous gas evolution can be observed and the solution was stirred at room temperature for 5 min. The previous orange solution turned yellow and after removal of the solvent the complexes were suspended in toluene and filtered over celite. The remaining filter was extracted with additional portions of toluene. The organic phases were combined and the solvent was removed under vacuum. The resulting reaction product was washed with pentane and dried in vacuum. Crystals were either obtained by slow evaporation of pentane solutions, or layering a toluene solution of the complexes with pentane.

[Ru(PCy$_3$)$_2$HCl(BAnil)] (6)

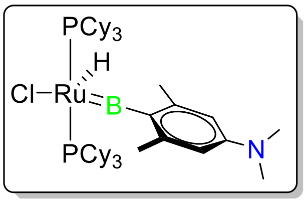

Li[AnilBH$_3$]·1.5 THF (71 mg, 0.255 mmol, 1.5 equiv.)

yield: 50% (73 mg, 0.085 mmol)

^{1}H NMR (500.1 MHz, C_6D_6, 296 K): δ (ppm) = –15.15 (t, $^{2}J_{P\text{-}H}$ = 18.3 Hz, 1H, Ru-*H*), 1.13-1.28 (m, 12H, *Cy*), 1.28-1.39 (m, 12H, *Cy*), 1.65 (m, 6H, *Cy*), 1.70-1.85 (m, 24H, *Cy*), 2.38-2.51 (m, 12H, *Cy*; s, 6H, NC*H*$_3$), 2.89 (s, 3H, C*H*$_3^{ortho}$-Anil), 3.25 (s, 3H, C*H*$_3^{ortho}$-Anil), 6.22 (m, 2H, C*H*^{meta}-Anil).

^{11}B NMR (160.5 MHz, C_6D_6, 296 K): δ (ppm) = 110.9 (br. s).

^{13}C{^{1}H} NMR (125.8 MHz, C_6D_6, 296 K): δ (ppm) = 23.4 (CH_3, *C*H$_3^{ortho}$-Anil), 24.4 (CH_3, *C*H$_3^{ortho}$-Anil), 27.1 (CH_2, *C*H$_2$-Cy), 28.1 (CH_2, *C*H$_2$-Cy), 28.4 (CH_2, *C*H$_2$-Cy), 30.6 (CH_2, *C*H$_2$-Cy), 31.1 (CH, *C*H-Cy), 37.1 (CH, *C*H-Cy), 39.4 (CH_3, N*C*H$_3$), 110.4 (CH, *C*H^{meta}-Anil), 111.1 (CH, *C*H^{meta}-Anil), 128.6 (C_q, *C*$_q$-B), 142.5 (C_q, C_qCH$_3$-Anil), 146.29 (C_q, *C*$_q$CH$_3$-Anil), 151.4 (C_q, C_qN-Anil).

^{31}P{^{1}H} NMR (202.5 MHz, C_6D_6, 296 K): δ (ppm) = 50.2 (s).

ESI-MS (Toluene) positive *m/z*, [M] 857.4633 (calculated: 857.4669).

[Ru(PCy$_3$)$_2$HCl(BDur)] (7)

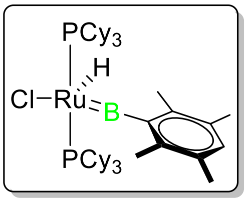

DurBH$_2$ (37 mg, 0.255 mmol, 1.5 equiv.)

yield: 74% (106 mg, 0.13 mmol)

^{1}H NMR (500.1 MHz, C_6D_6, 296 K): δ (ppm) = –14.61 (t, $^{2}J_{P\text{-}H}$ = 18.5 Hz, 1H, Ru-*H*), 1.13-1.35 (m, 12H, *Cy*), 1.25-1.39 (m, 6H, *Cy*), 1.56-1.83 (m, 30H, *Cy*), 1.98-2.03 (m, 6H, *Cy*), 2.03 (s, 6H C*H*$_3$-Dur), 2.04 (s, 3H, C*H*$_3$-Dur) 2.33-2.48 (m , 12H, *Cy*), 2.85 (s, 3H, C*H*$_3$-Dur), 3.25 (s, 3H, C*H*$_3$-Dur), 6.81 (s, CH, C*H*^{para}-Dur).

^{11}B NMR (160.5 MHz, C_6D_6, 296 K): δ (ppm) = 110.1 (br. s).

^{13}C{^{1}H} NMR (125.8 MHz, C_6D_6, 296 K): δ (ppm) = 19.5 (CH_3, *C*H$_3$-Dur), 19.5 (CH_3, CH$_3$-Dur), 19.8 (CH_3, CH$_3$-Dur), 21.5 (CH_3, CH$_3$-Dur), 27.0 (CH_2, *C*H$_2$-Cy), 28.0 (CH_2, *C*H$_2$-Cy), 28.3 (CH_2, *C*H$_2$-Cy), 30.5 (CH_2, *C*H$_2$-Cy), 30.9 (CH_2, *C*H$_2$-Cy), 36.9 (CH, *C*H-Cy), 133.8 (CH, *C*H^{para}-Dur), 134.0 (C_q, *C*$_q$CH$_3$-Dur), 134.6 (C_q, *C*$_q$CH$_3$-Dur), 136.6 (C_q, *C*$_q$CH$_3$-Dur), 138.8 (C_q, *C*$_q$-B), 140.6 (C_q, *C*$_q$CH$_3$-Dur).

^{31}P{^{1}H} NMR (202.5 MHz, C_6D_6, 296 K): δ (ppm) = 50.8 (s).

ESI-MS (Toluene) positive *m/z* [M] 842.4542 (calculated: 842.4560), [M-Cl] 807.4821 (calculated: 807.4872), [M-Cl-H] 806.4788 (calculated: 806.4794).

[Ru(PCy$_3$)$_2$HCl(B^{o}FXyl)] (8)

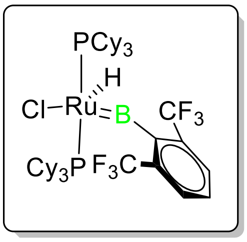

Li[^{o}FXylBH$_3$]·1.5 Et$_2$O (88 mg, 0.255 mmol, 1.5 equiv.)

yield: 54% (86 mg, 0.09 mmol)

The product is highly soluble in pentane, whereby additional 40 mg could be obtained by fractional crystallization. However, this sample is contaminated by grease.

^{1}H NMR (500.1 MHz, C_6D_6, 296 K): δ(ppm) = –15.52 (m, 1H, Ru-*H*), 1.03 (m, 6H, *Cy*), 1.23 (m, 6H, *Cy*), 1.35 (m, 6H, *Cy*), 1.61 (m, 18, *Cy*), 1.80 (m, 12H, *Cy*), 1.96 (m, 6H, *Cy*), 2.43 (m, 6H, *Cy*), 2.55 (m, 6H, *Cy*), 6.71 (t, $^3J_{H\text{-}H}$ = 7.93 Hz, 1H, CH^{para}-^{o}CF$_3$), 7.25 (d, $^3J_{H\text{-}H}$ = 7.9 Hz, 1H, CH^{meta}), 7.35 (d, $^3J_{H\text{-}H}$ = 7.93 Hz, 1H, CH^{meta}).
^{11}B NMR (160.5 MHz, C_6D_6, 296 K): δ (ppm) = 98.8 (br s).
^{13}C{^{1}H} NMR (125.8 MHz, C_6D_6, 296 K): δ (ppm) = 27.1 (CH$_2$, *C*H$_2$-Cy), 27.9 (CH$_2$, *C*H$_2$-Cy), 28.3 (CH$_2$, *C*H$_2$-Cy), 28.0 (CH$_2$, *C*H$_2$-Cy), 30.2 (CH$_2$, *C*H$_2$-Cy), 31.0 (CH$_2$, *C*H$_2$-Cy), 36.3 (CH, *C*H-Cy), 124.4 (d, $^1J_{C\text{-}F}$ = 275.8 Hz, *C*F$_3$), 124,7 (d, $^1J_{C\text{-}F}$ 276.7 Hz, *C*F$_3$), 129.3 (CH, *C*H^{meta}-^{o}CF$_3$), 129.8 (CH, *C*H^{para}-^{o}CF$_3$), 130.5 (CH, *C*H^{meta}-^{o}CF$_3$), 133.1 (d, $^2J_{C\text{-}F}$ = 30.8 Hz, C$_q$, *C*CF$_3$), 137.2 (d, $^2J_{C\text{-}F}$ = 30.5 Hz, C$_q$, *C*CF$_3$), 141.6 (C$_q$, *C*$_q$-B).
^{19}F NMR (125.8 MHz, C_6D_6, 296 K): δ (ppm) = –54.6 (s), –53.6 (m).
^{31}P{^{1}H} NMR (202.5 MHz, C_6D_6, 296 K): δ (ppm) = 46.5 (s).
ESI-MS (Toluene) positive *m/z*, [M+H] 923.3718 (calculated: 923.3760), [M-H] 921.3698 (calculated: 921.3604), [M-Cl] 887.3958 (calculated: 887.3993).

[Ru(PCy$_3$)$_2$HCl(σ^2-H$_2$B^{m}Xyl)]

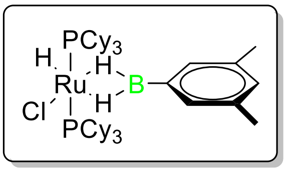

Li[^{m}XylBH$_3$]·1.5 THF (60 mg, 0.255 mmol, 1.5 equiv.)

^{11}B NMR (160.5 MHz, C_6D_6, 296 K): δ (ppm) = 46.7 (br s).
^{31}P{^{1}H} NMR (202.5 MHz, C_6D_6, 296 K): δ (ppm) = 53.6 (s).

[Ru(PCy$_3$)$_2$H(σ^2-H$_2$B(^{m}FXyl)$_2$)] (10)

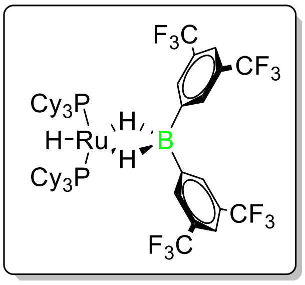

Li[^{m}FXylBH$_3$]·1.5 Et$_2$O (88 mg, 0.255 mmol, 1.5 equiv.)

Yield: 45% (85 mg, 0.077 mmol)

^{1}H NMR (400.1 MHz, C_6D_6, 296 K): δ (ppm) = –5.92 (br s, 2H, σ^2-B-*H*), 1.01-1.23 (m, 18H, *Cy*), 1.36-1.55 (m, 12H, *Cy*), 1.55-1.64 (m, 6H, *Cy*), 1.66-1.74 (m, 18H, *Cy*), 1.74-1.91 (m, 12H, *Cy*), 7.81 (s, 2H, CH^{para}-^{m}FXyl), 8.30 (br m, 4H, CH^{ortho}-^{m}FXyl).
^{13}C{^{1}H} NMR (100.6 MHz, C_6D_6, 296 K): δ (ppm) = 26.7 (CH$_2$, *C*H$_2$-Cy), 28.0 (CH$_2$, *C*H$_2$-Cy), 30.7 (CH$_2$, *C*H$_2$-Cy), 31.2 (CH$_2$, *C*H$_2$-Cy), 39.2 (CH, *C*H-Cy), 120.7 (CH, *C*H^{para}-^{m}FXyl), 124.8 (q, $^1J_{C-F}$ = 272.9 Hz, C$_q$, *C*F3), 130.8 (q, $^2J_{C-F}$ = 32.3 Hz, C$_q$, *C*CF$_3$), 133.36 (CH, *C*H^{ortho}-^{m}FXyl), 153.1 (C$_q$, *C*$_q$-B).
^{19}F NMR (376.5 MHz, C_6D_6, 296 K): δ (ppm) = –62.2 (s).
^{31}P{^{1}H} NMR (161.9 MHz, C_6D_6, 296 K): δ (ppm) = 50.2 (s).
ESI-MS (Toluene) positive *m/z*, [M] 1102.4143 (calculated: 1102.4289), [M-2H] 1100.4133 (calculated: 1100.4132).

[Ir(PCy$_3$)$_2$H$_2$(κ^2-H$_3$BDur)] (11)

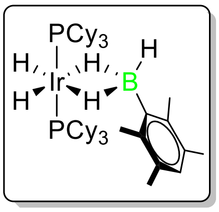

To a suspension of [Ir(PCy$_3$)$_2$H$_5$] (200 mg, 0.26 mmol, 1.0 equiv) in benzene, solid DurBH$_2$ (40 mg, 0.26 mmol, 1.0 equiv) was added. After gas evolution the reaction mixture was heated to 60 °C overnight. The white suspension turns into an orange solution containing a white solid. After cooling, all volatiles were removed by vacuum and the remaining reaction mixture was washed with pentane to afford a white solid, which was dried in vacuum. **Yield:** 64% (150 mg, 0.16 mmol).

^{1}H NMR (500.1 MHz, C_6D_6, 296 K): δ (ppm) = –19.83 (m, $^2J_{H-P}$ = 16.5 Hz, 2H, Ir-*H*), –6.37 (br s, 2H, κ^2-B-*H*), 1.11-1.44 (m, 24H, *Cy*), 1.55-1.79 (m, 21H, *Cy*), 1.79-1.93 (m, 12H, *Cy*), 2.22 (m, 9H, *Cy*), 2.33 (s, 6H, *C*H$_3$-Dur), 2.57 (s, 6H, *C*H$_3$-Dur), 6.91 (s, CH, *C*H^{para}), 8.47 (br s, 1H, B-H).
^{11}B NMR (160.5 MHz, C_6D_6, 296 K): δ (ppm) = 20.6 (br s).
^{13}C{^{1}H} NMR (125.8 MHz, C_6D_6, 296 K): δ (ppm) = 18.7 (CH$_3$, *C*H$_3$-Dur), 20.7 (CH$_3$, *C*H$_3$-Dur), 26.9 (CH$_2$, *CH$_2$*-Cy), 27.2 (CH$_2$, *CH$_2$*-Cy), 27.9 (CH$_2$, *CH$_2$*-Cy), 28.1 (CH$_2$, *CH$_2$*-Cy), 30.9 (CH$_2$, *CH$_2$*-Cy), 31.2 (CH$_2$, *CH$_2$*-Cy), 36.9 (CH, *CH*-Cy), 37.9 (CH, *CH*-Cy), 129.2 (CH, *C*H^{para}-Dur), 132.3 (C$_q$, *C*$_q$-Dur), 133.7 (C$_q$, *C*$_q$-Dur), 155.6 (C$_q$, *C*$_q$-B).
^{31}P{^{1}H} NMR (202.5 MHz, C_6D_6, 296 K): δ (ppm) = 33.8 (m).
ESI-MS (Toluene) positive *m/z*, [M-4H]$^+$ 898.5390 (calculated: 898.5358). [M-6H]$^+$ 896.5368 (calculated: 896.5301).

NMR Spectra:

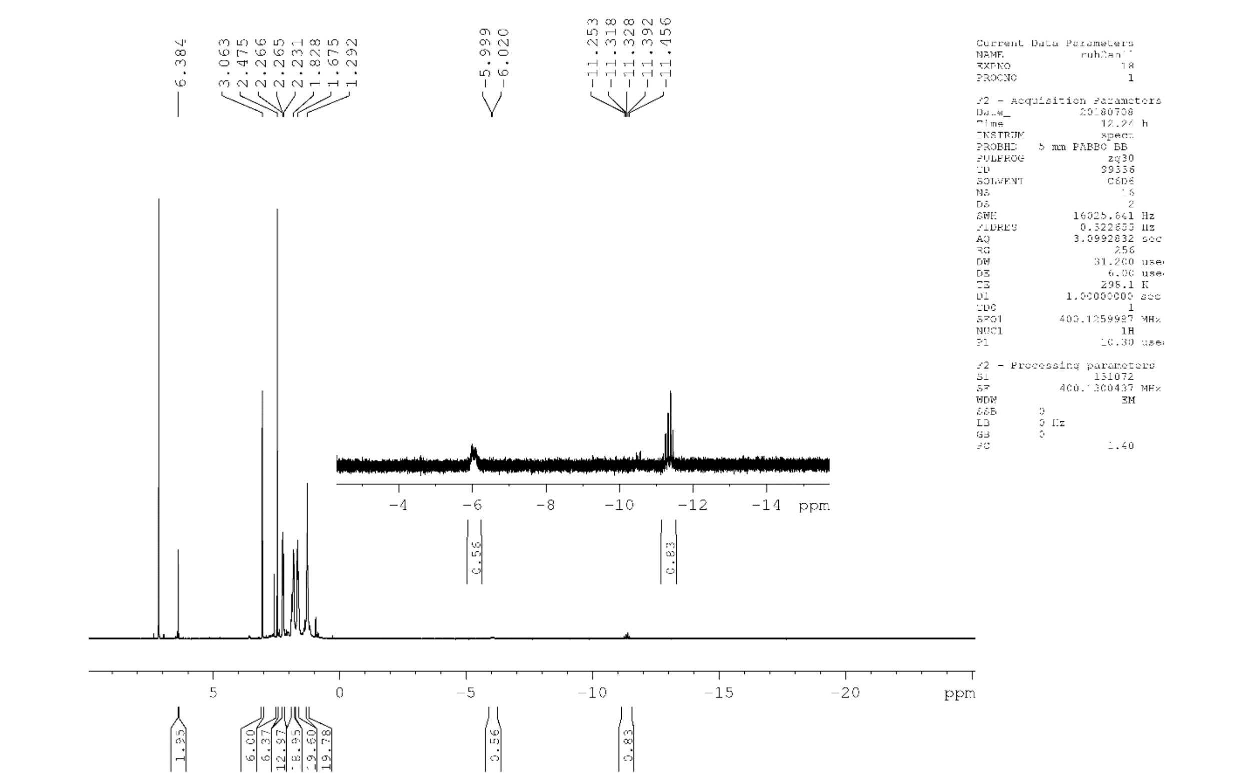

Figure S2: ^{1}H NMR spectrum of **[Ru(PCy$_3$)$_2$H$_2$(σ^2-H$_2$BAnil)] (1)** in C_6D_6

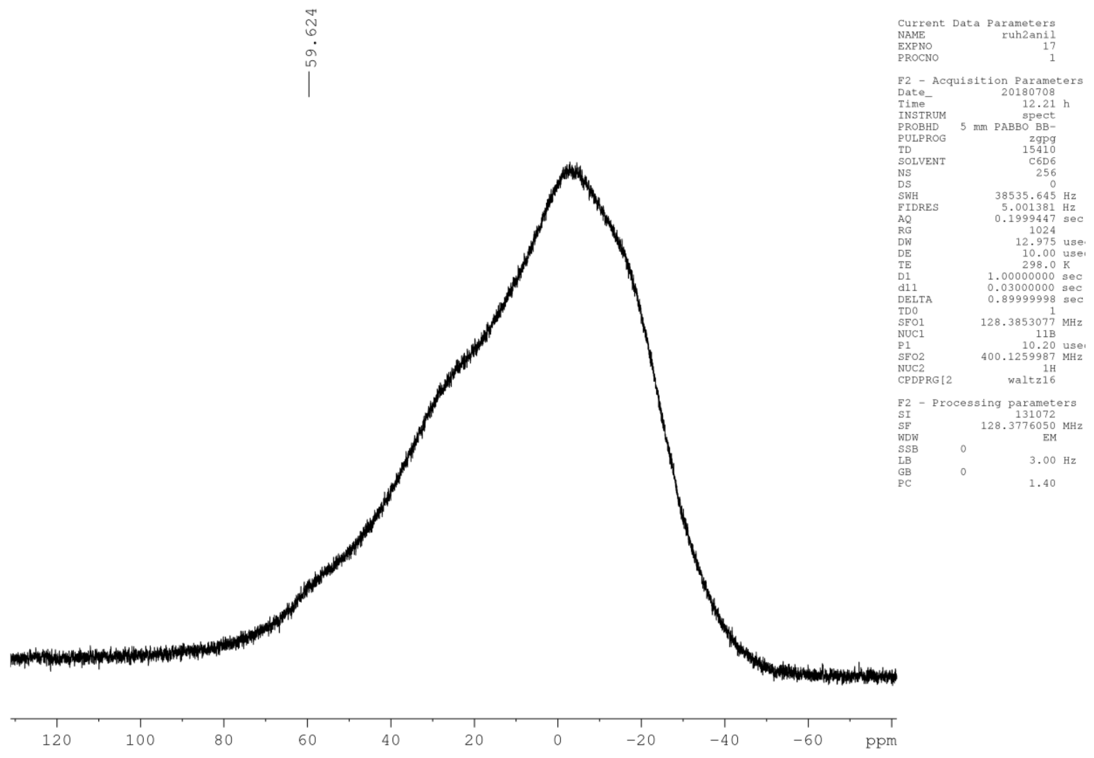

Figure S3: ^{11}B NMR spectrum of **[Ru(PCy$_3$)$_2$H$_2$(σ^2-H$_2$BAnil)] (1)** in C_6D_6

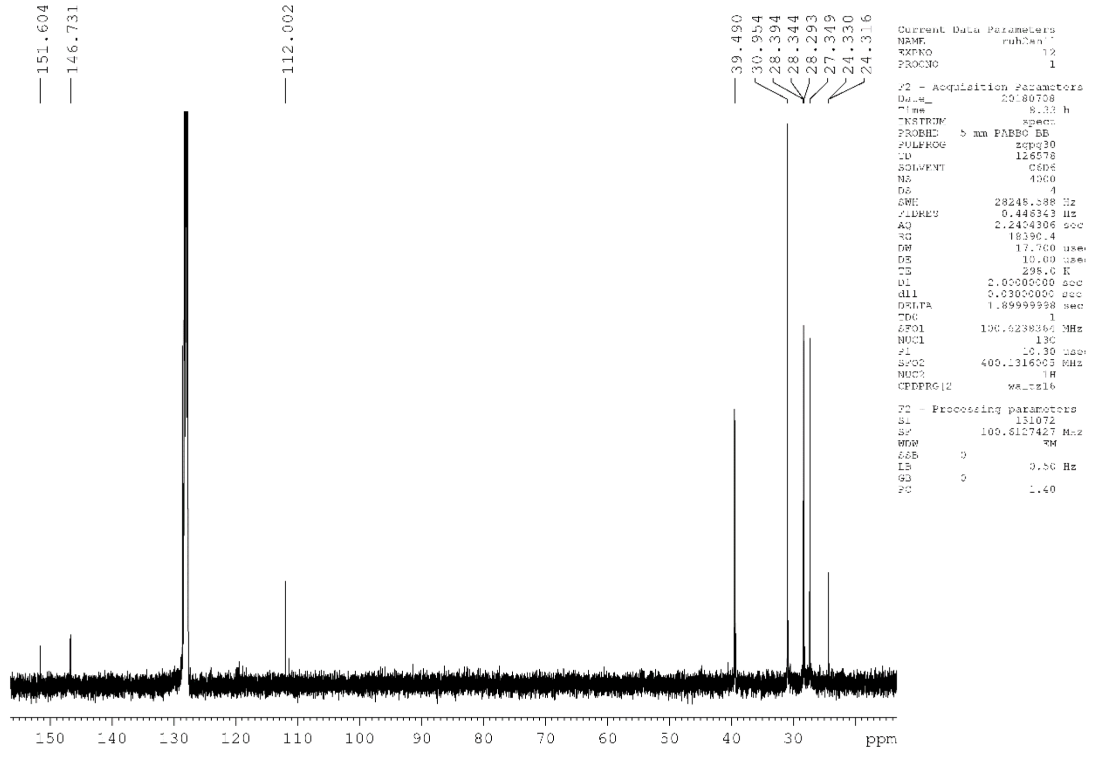

Figure S4: $^{13}C\{^{1}H\}$ NMR spectrum of **$[Ru(PCy_3)_2H_2(\sigma^2\text{-}H_2BAnil)]$ (1)** in C_6D_6

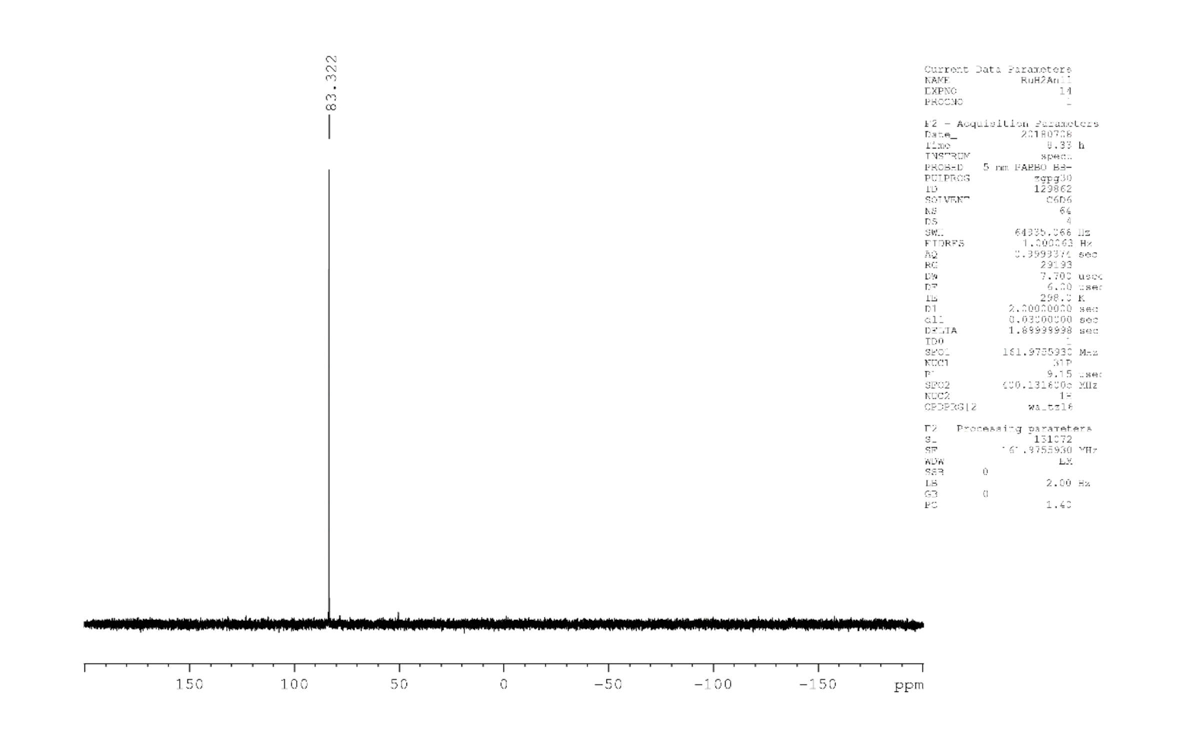

Figure S5: $^{31}P\{^{1}H\}$ NMR spectrum of **$[Ru(PCy_3)_2H_2(\sigma^2\text{-}H_2BAnil)]$ (1)** in C_6D_6

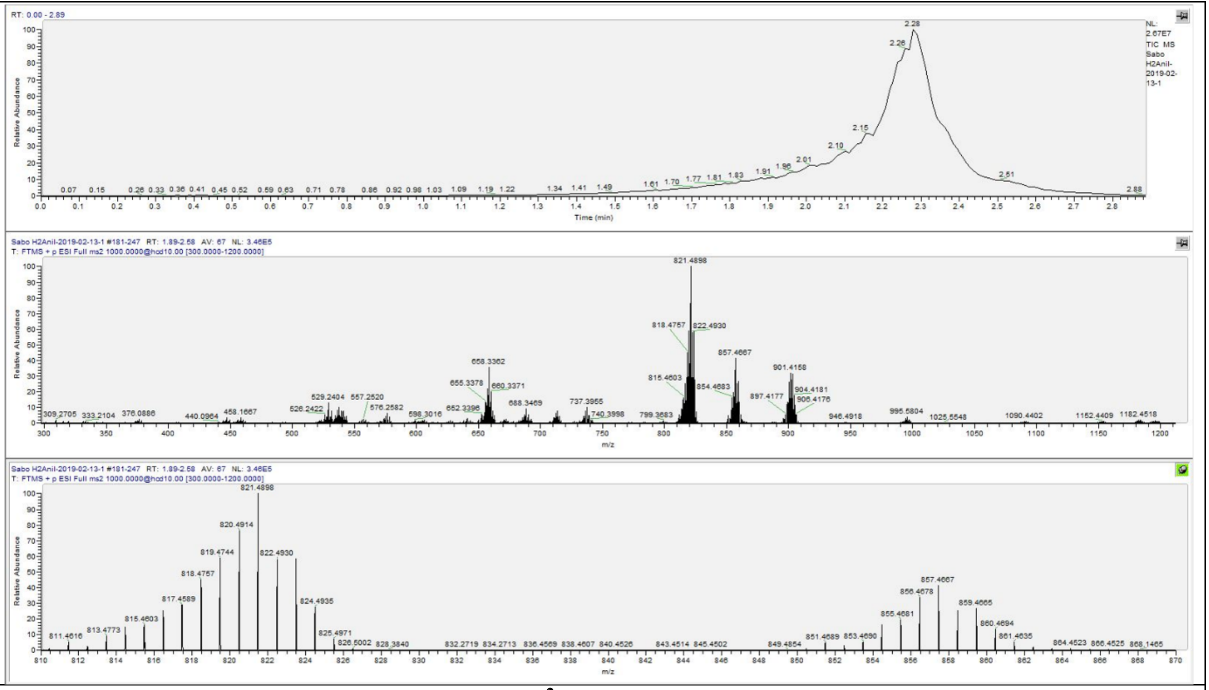

Figure S6: Mass spectrum of **$[Ru(PCy_3)_2H_2(\sigma^2\text{-}H_2BAnil)]$ (1)**

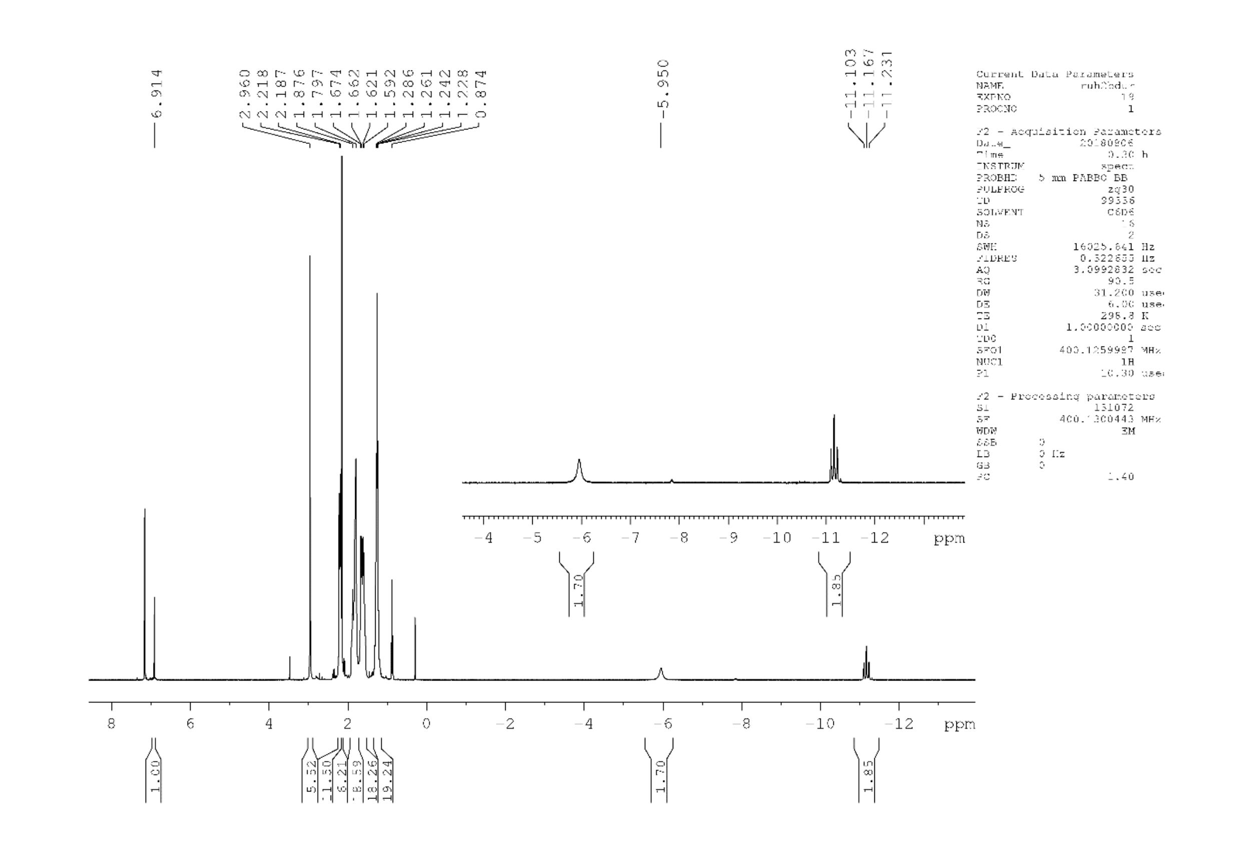

Figure S7: ^{1}H NMR spectrum of **[Ru(PCy$_3$)$_2$H$_2$(σ^2-H$_2$BDur)] (2)** in C_6D_6

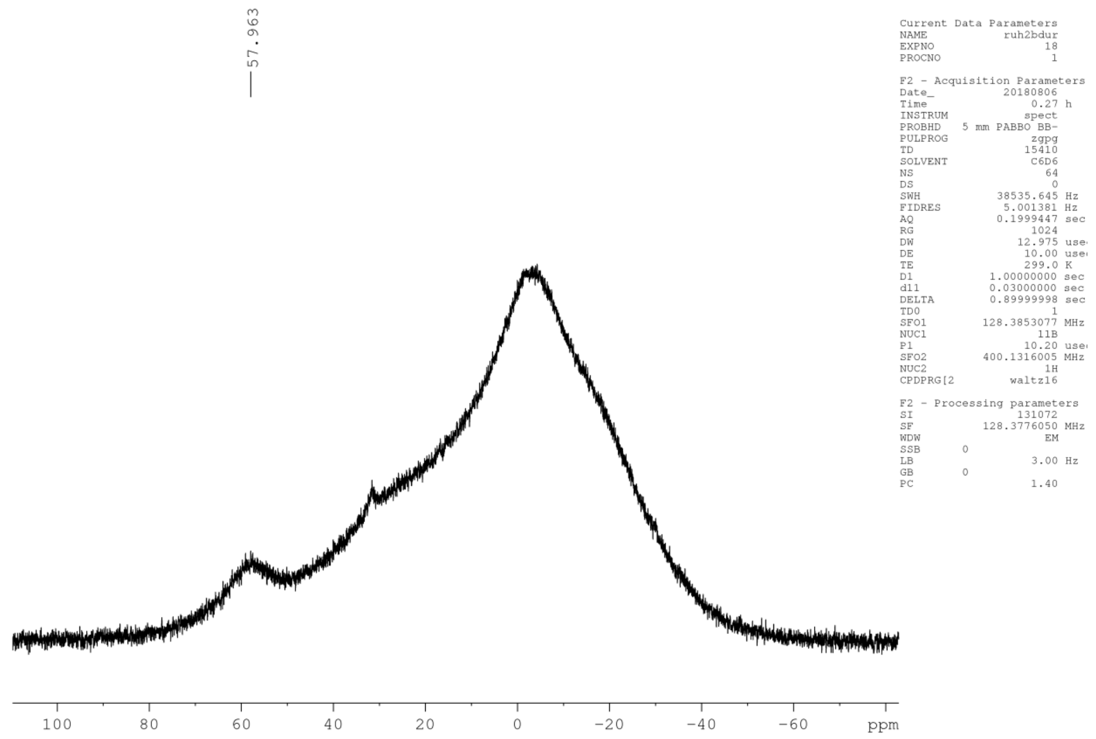

Figure S8: ^{11}B NMR spectrum of **[Ru(PCy$_3$)$_2$H$_2$(σ^2-H$_2$BDur)] (2)** in C_6D_6

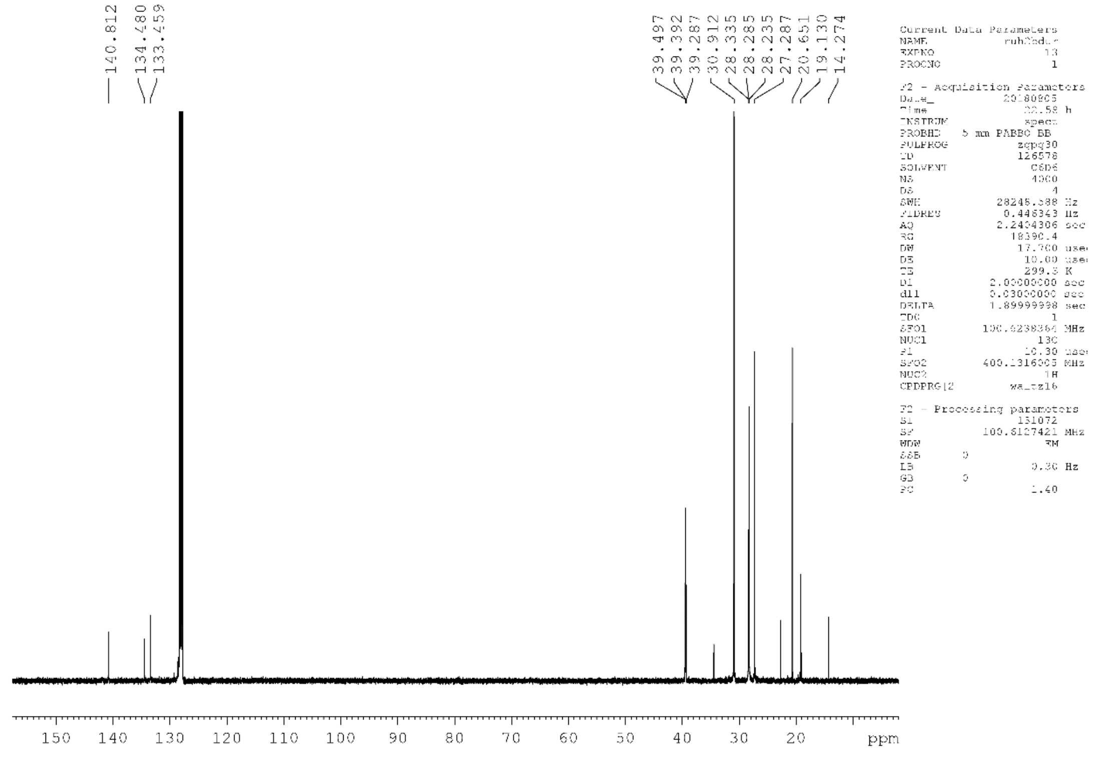

Figure S9: $^{13}C\{^{1}H\}$ NMR spectrum of **$[Ru(PCy_3)_2H_2(\sigma^2\text{-}H_2BDur)]$ (2)** in C_6D_6

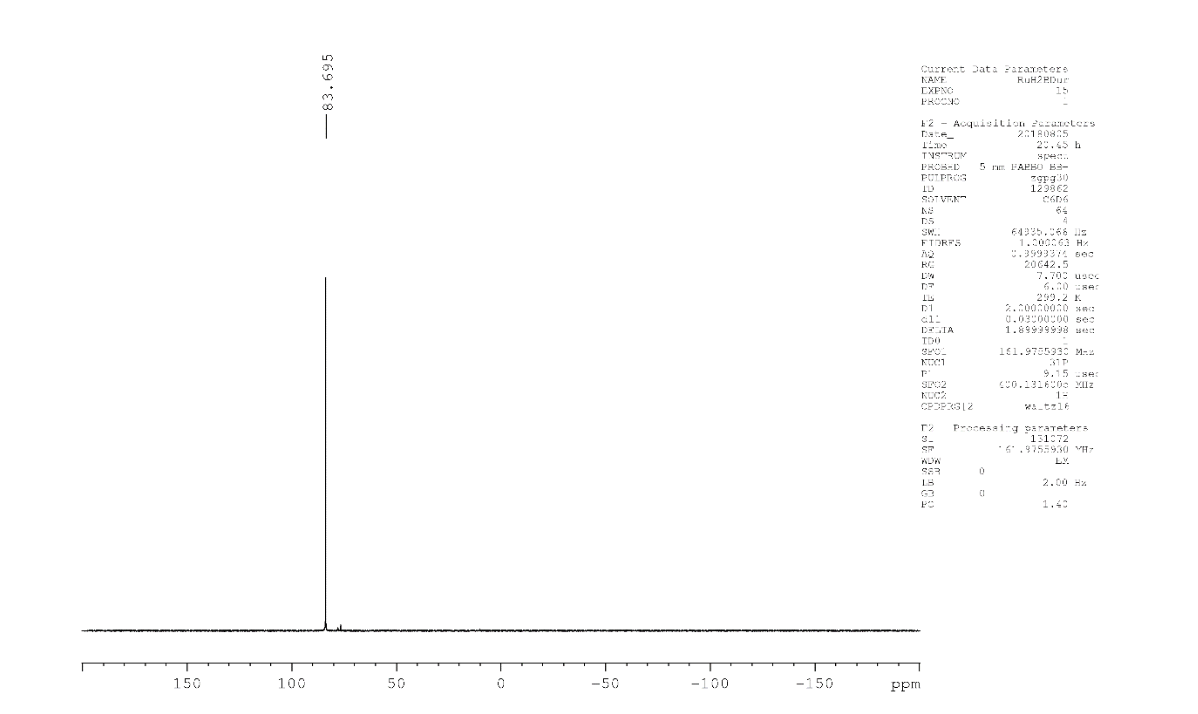

Figure S10: $^{31}P\{^{1}H\}$ NMR spectrum of **$[Ru(PCy_3)_2H_2(\sigma^2\text{-}H_2BDur)]$ (2)** in C_6D_6

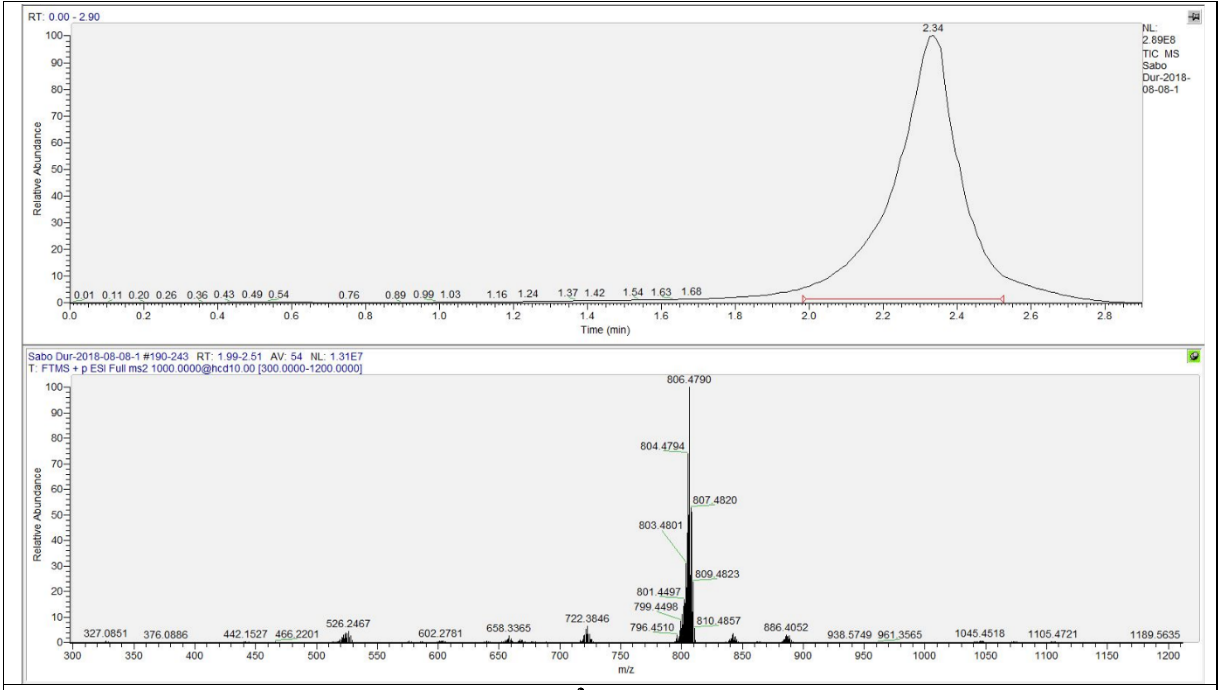

Figure S11: Mass spectrum of **$[Ru(PCy_3)_2H_2(\sigma^2\text{-}H_2BDur)]$ (2)**

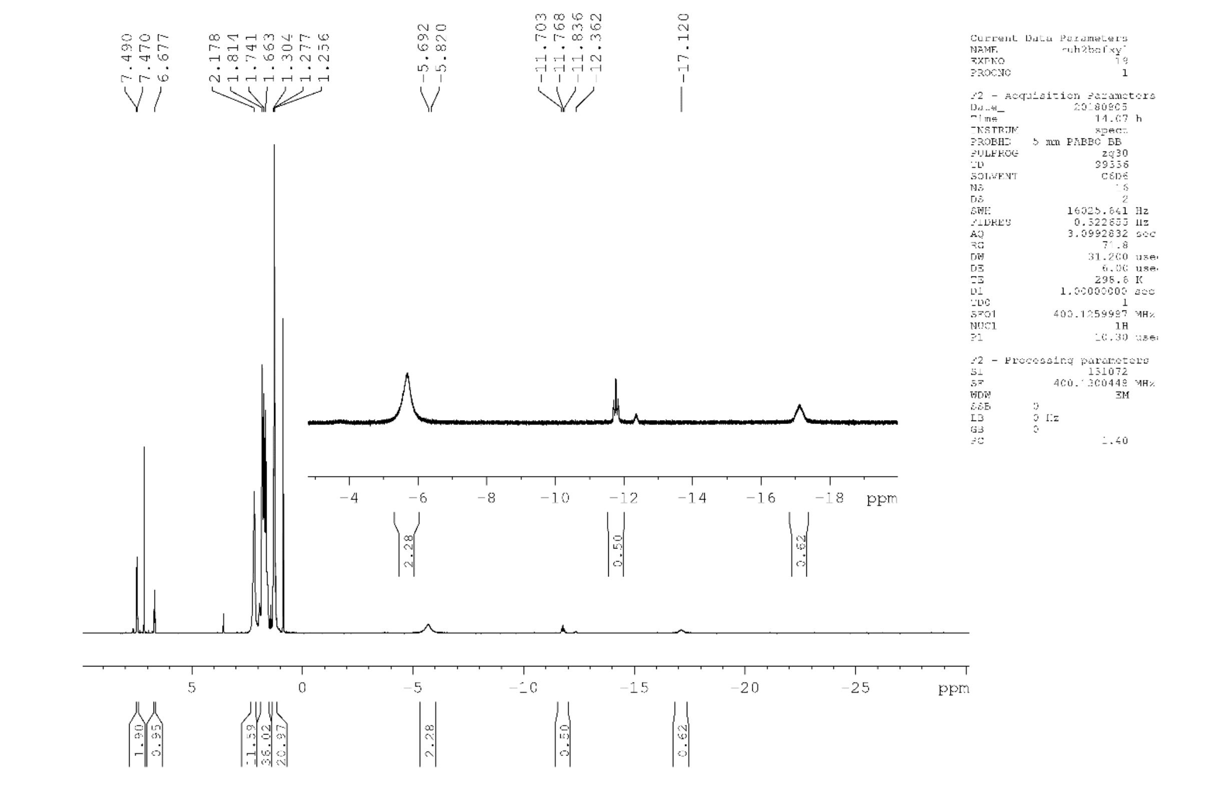

Figure S12: ^{1}H NMR spectrum of **[Ru(PCy$_3$)$_2$H$_2$(σ^2-H$_2$B^{o}FXyl)] (3)** in C_6D_6.

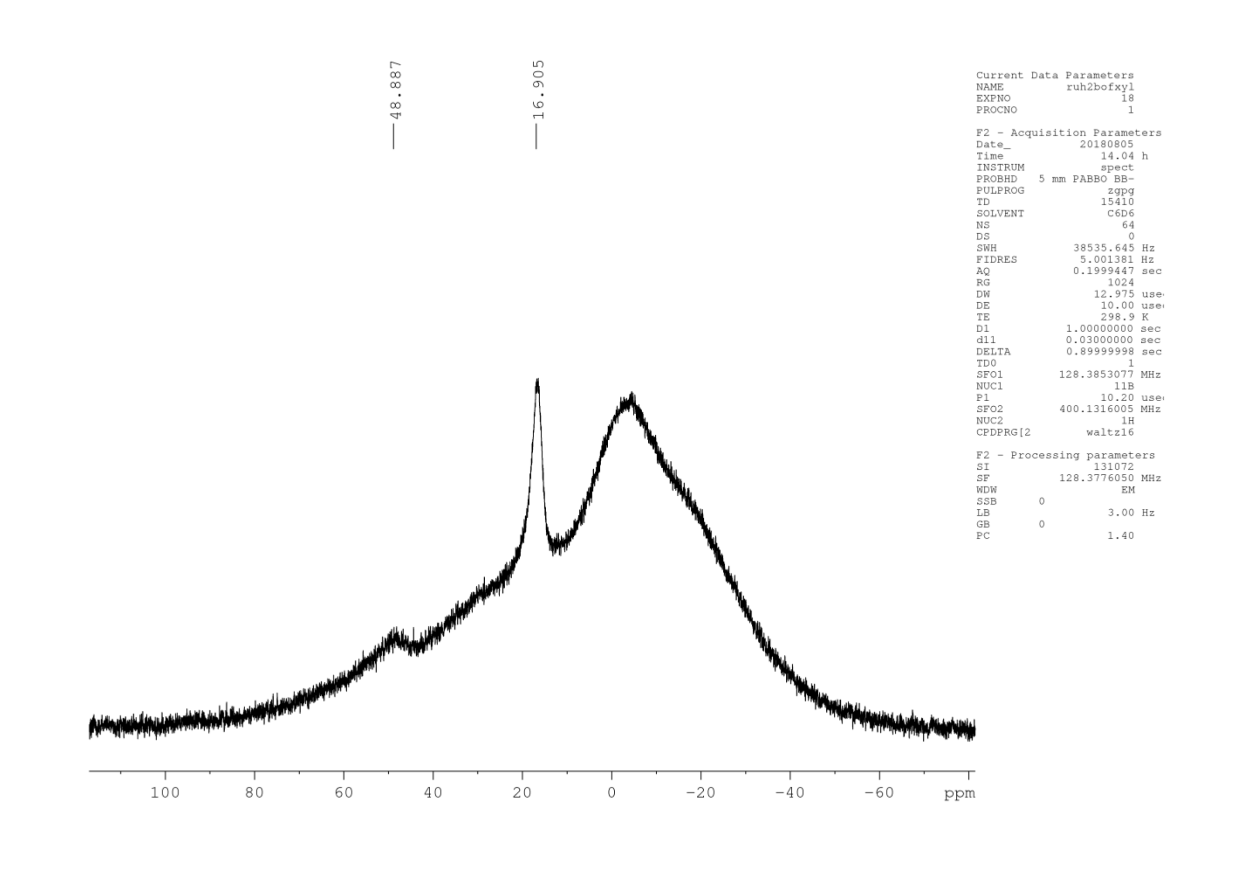

Figure S13: ^{11}B NMR spectrum of **[Ru(PCy$_3$)$_2$H$_2$(σ^2-H$_2$B^{o}FXyl)] (3)** in C_6D_6.

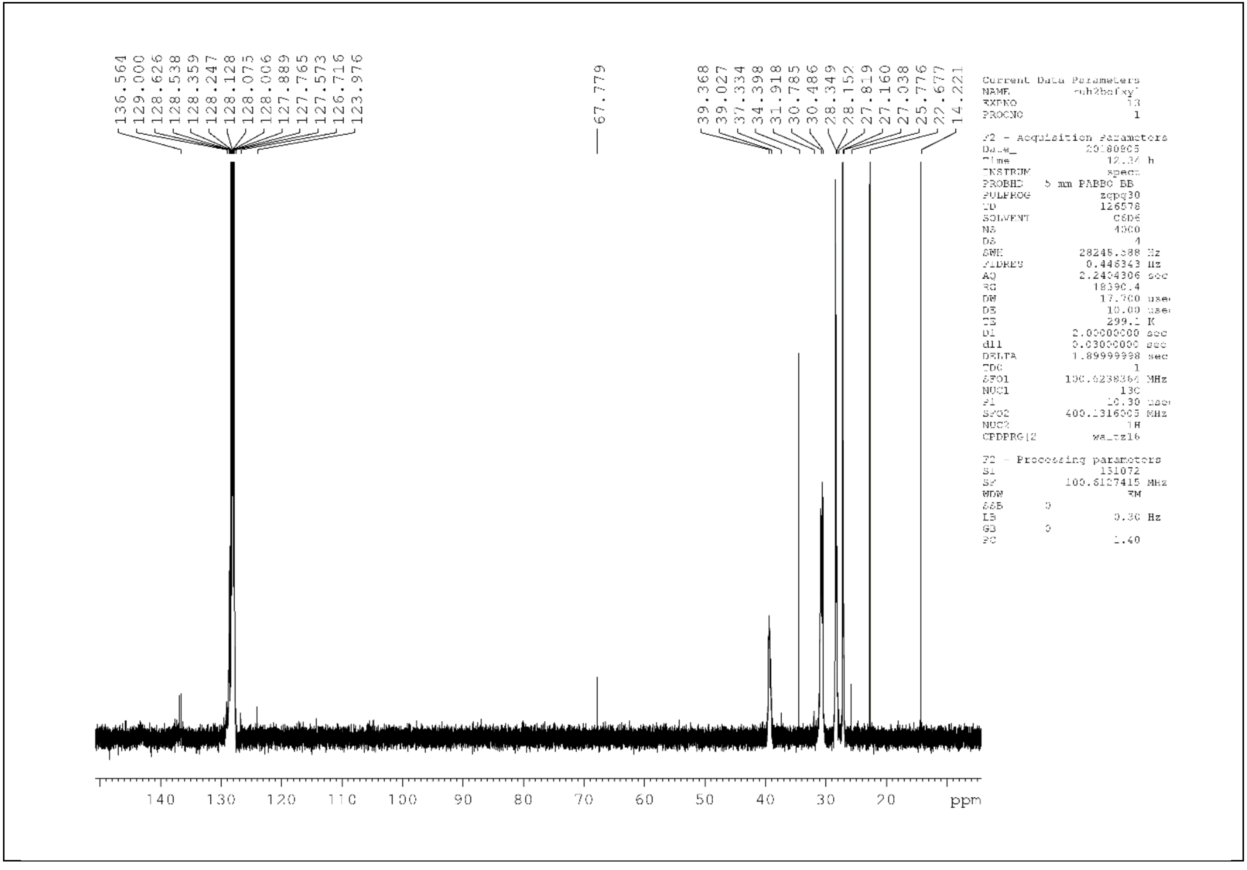

Figure S14: $^{13}C\{^{1}H\}$ NMR spectrum of **[Ru(PCy$_3$)$_2$H$_2$(σ^2-H$_2$B^{o}FXyl)] (3)** in C_6D_6.

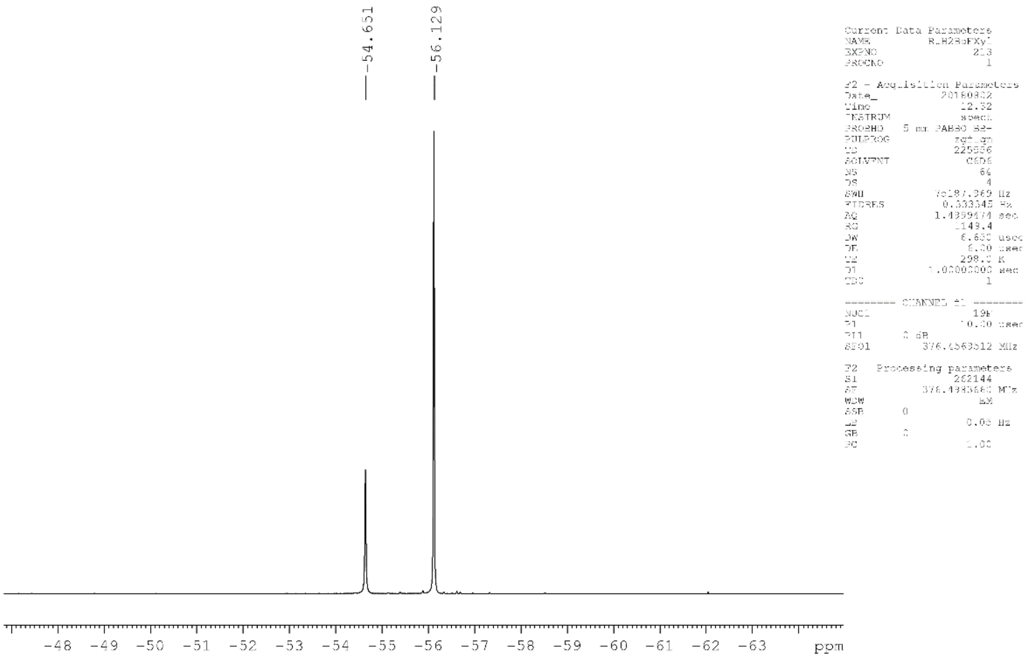

Figure S15: ^{19}F NMR spectrum of **[Ru(PCy$_3$)$_2$H$_2$(σ^2-H$_2$B^{o}FXyl)] (3)** in C_6D_6.

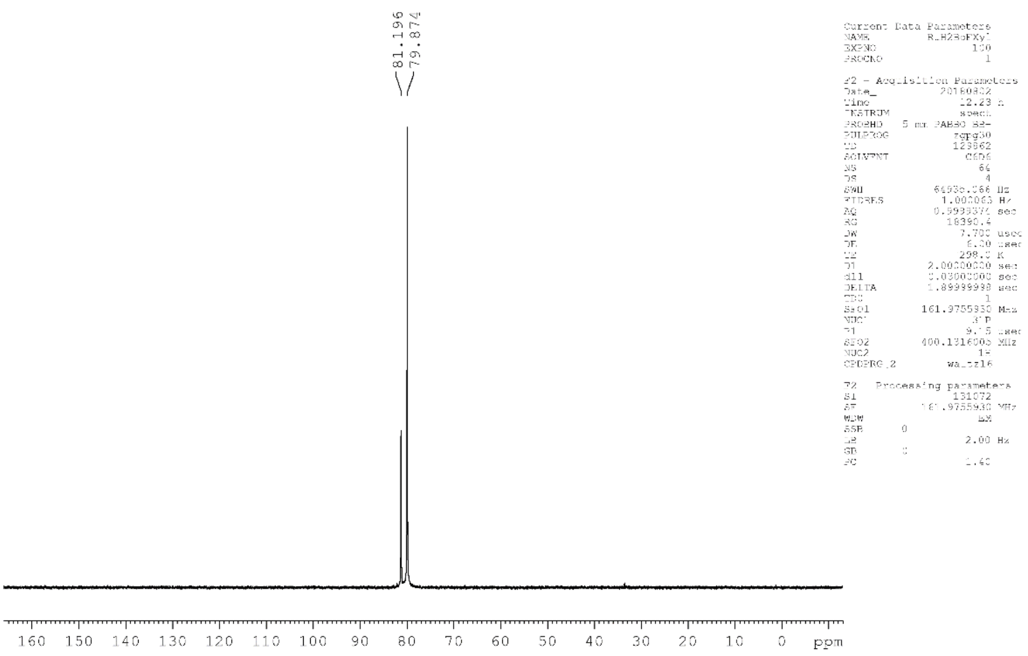

Figure S15: $^{31}P\{^{1}H\}$ NMR spectrum of **[Ru(PCy$_3$)$_2$H$_2$(σ^2-H$_2$B^{o}FXyl)] (3)** in C_6D_6.

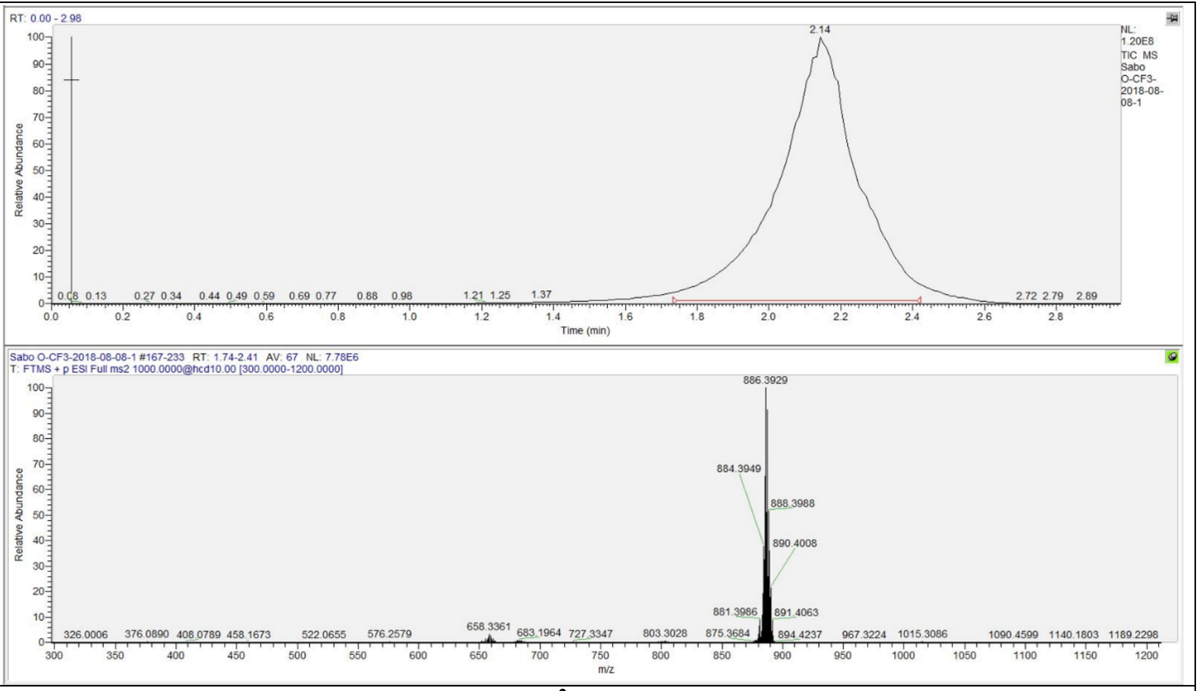

Figure S16: Mass spectrum of **[Ru(PCy$_3$)$_2$H$_2$(σ^2-H$_2$B^{o}FXyl)] (3)**

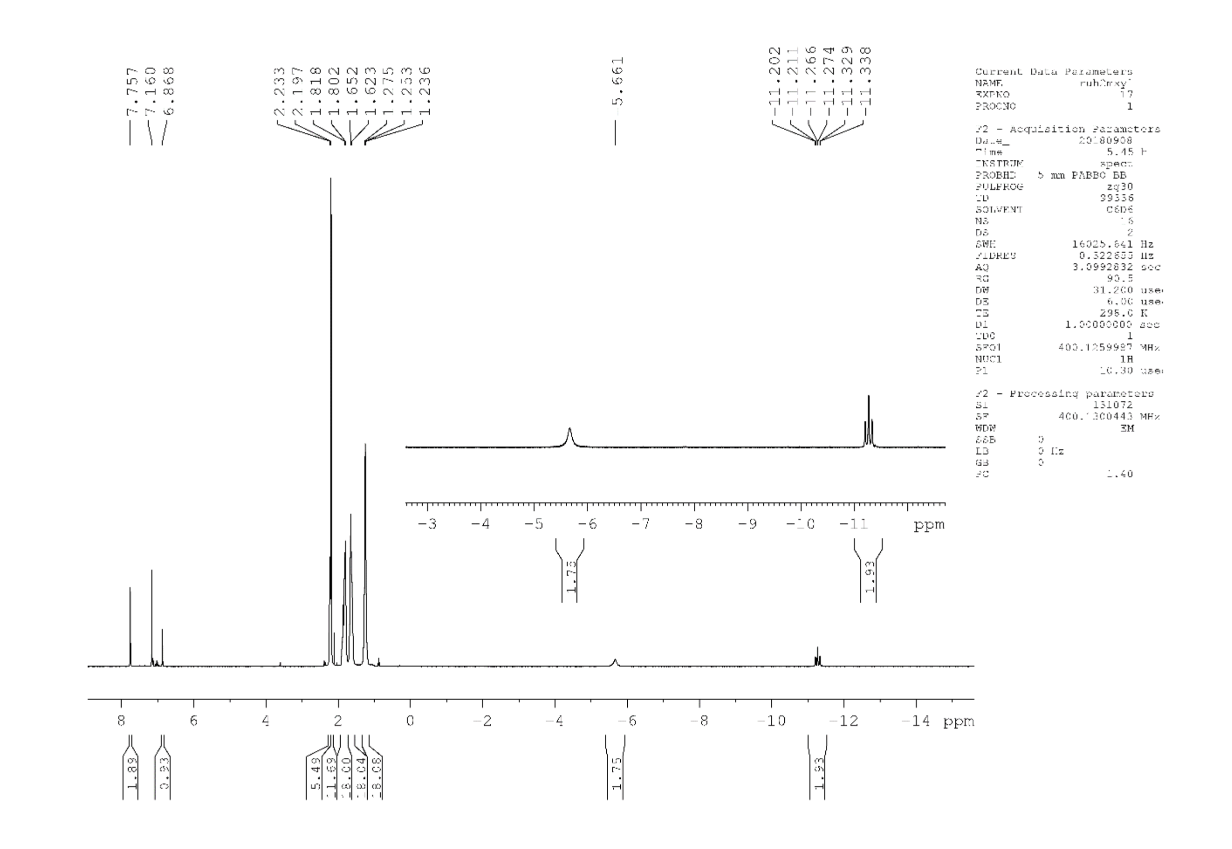

Figure S17: ^{1}H NMR spectrum of **[Ru(PCy$_3$)$_2$H$_2$(σ^2-H$_2$B^{m}Xyl)] (4)** in C_6D_6

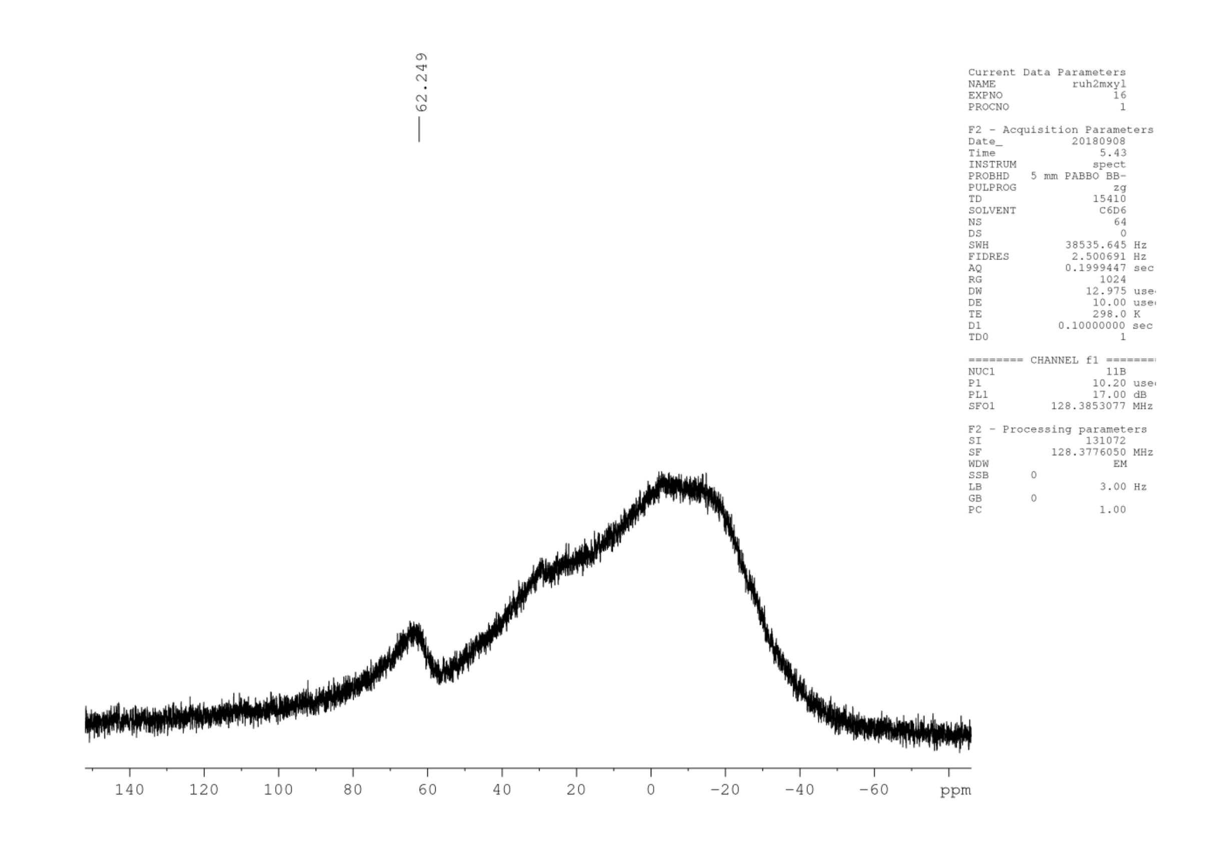

Figure S18: ^{11}B NMR spectrum of **[Ru(PCy$_3$)$_2$H$_2$(σ^2-H$_2$B‴Xyl)] (4)** in C_6D_6

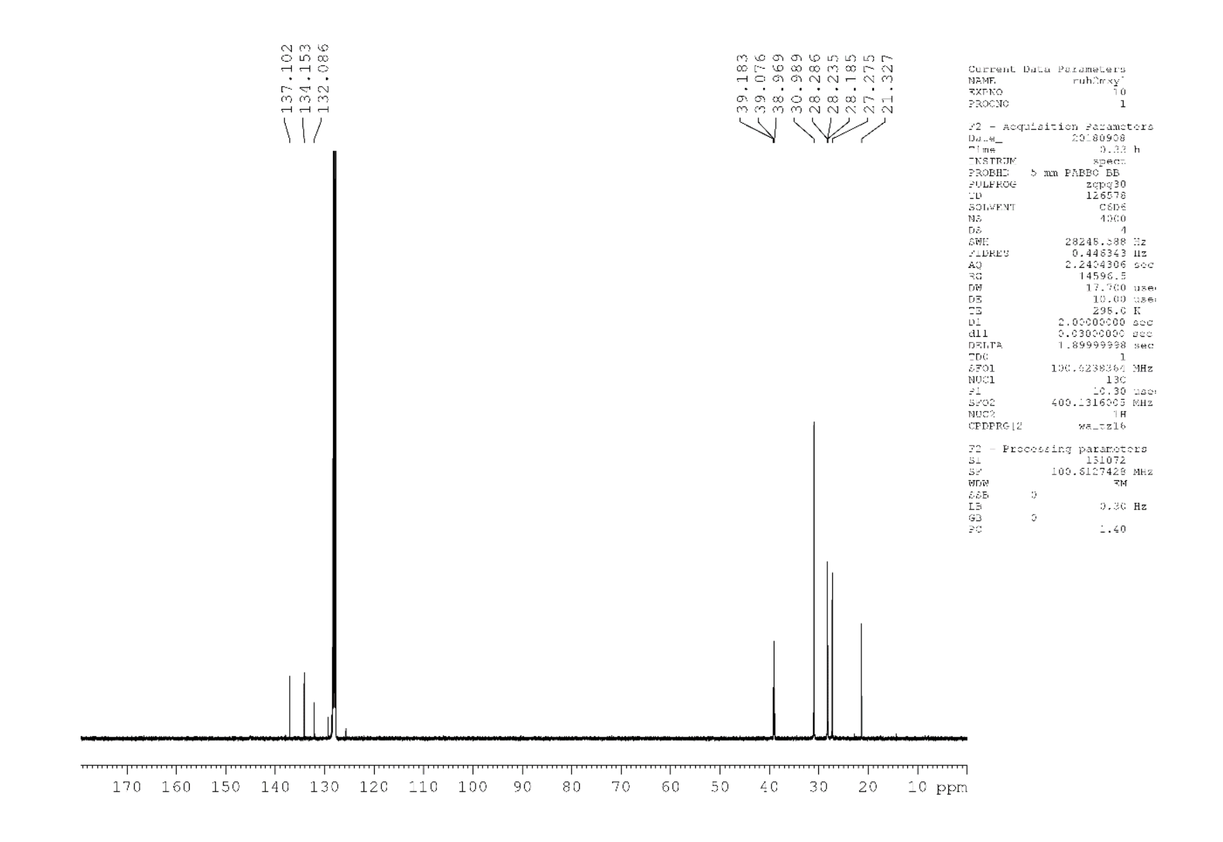

Figure S19: $^{13}C\{^{1}H\}$ NMR spectrum of **[Ru(PCy$_3$)$_2$H$_2$(σ^2-H$_2$B‴Xyl)] (4)** in C_6D_6

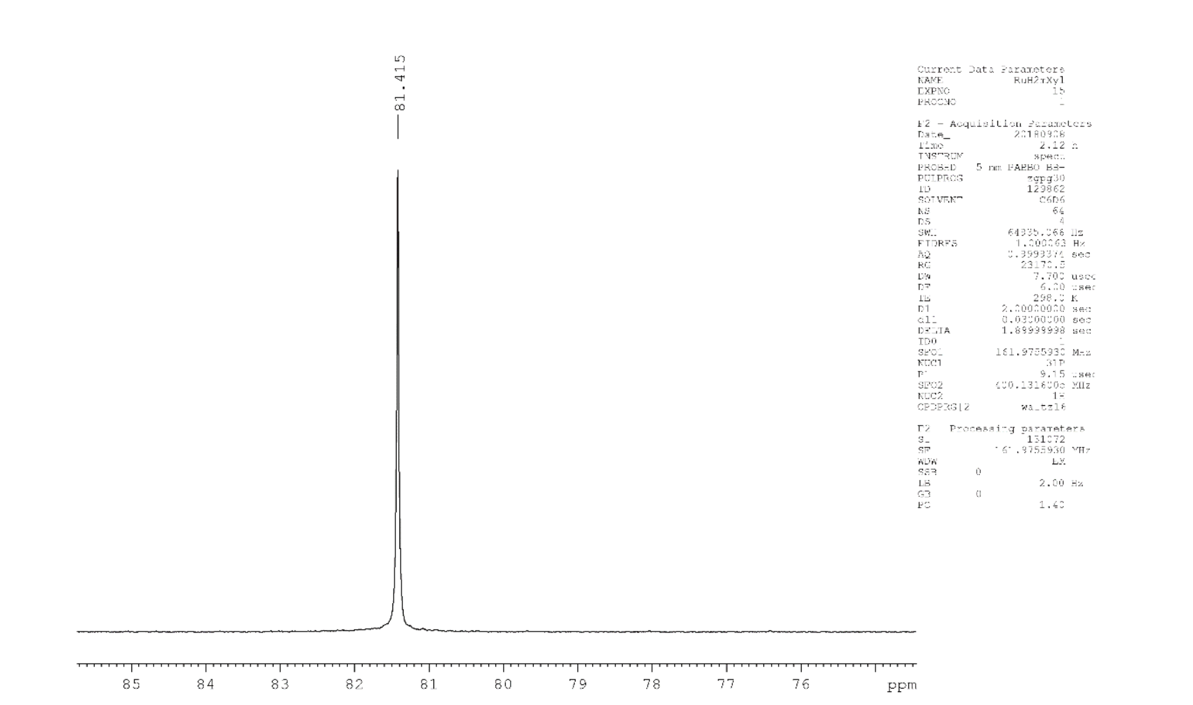

Figure S20: $^{31}P\{^{1}H\}$ NMR spectrum of **[Ru(PCy$_3$)$_2$H$_2$(σ^2-H$_2$B‴Xyl)] (4)** in C_6D_6

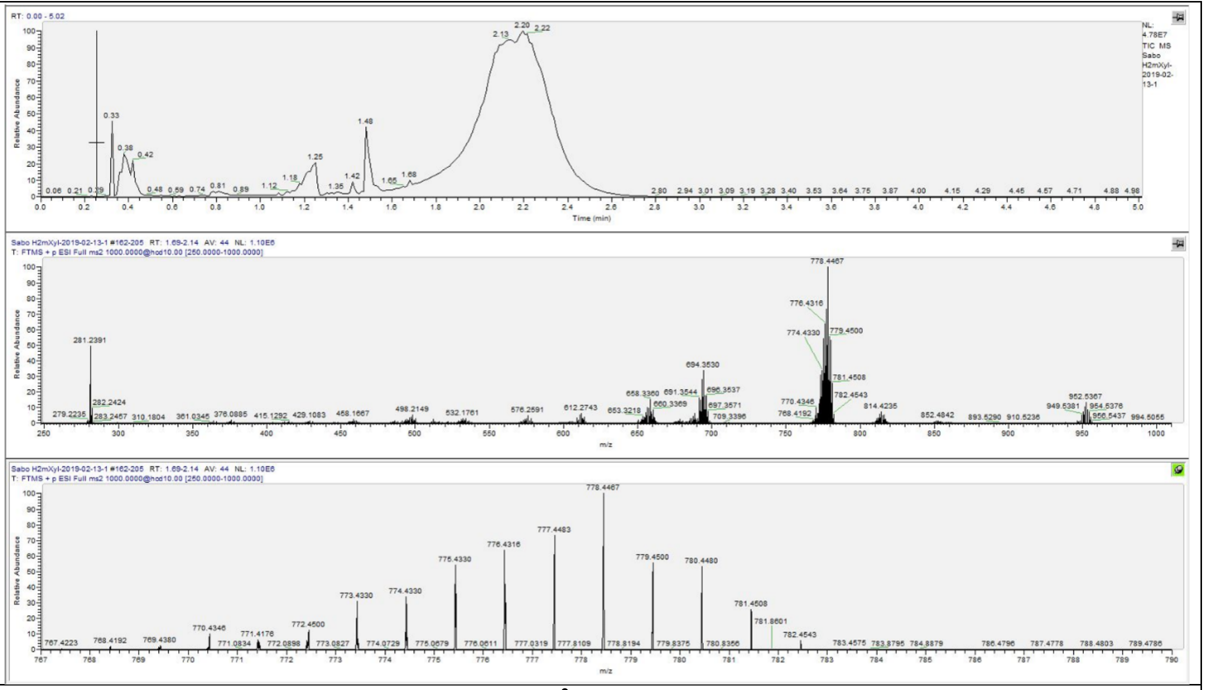

Figure S21: Mass spectrum of **[Ru(PCy$_3$)$_2$H$_2$(σ^2-H$_2$B‴Xyl)] (4)**

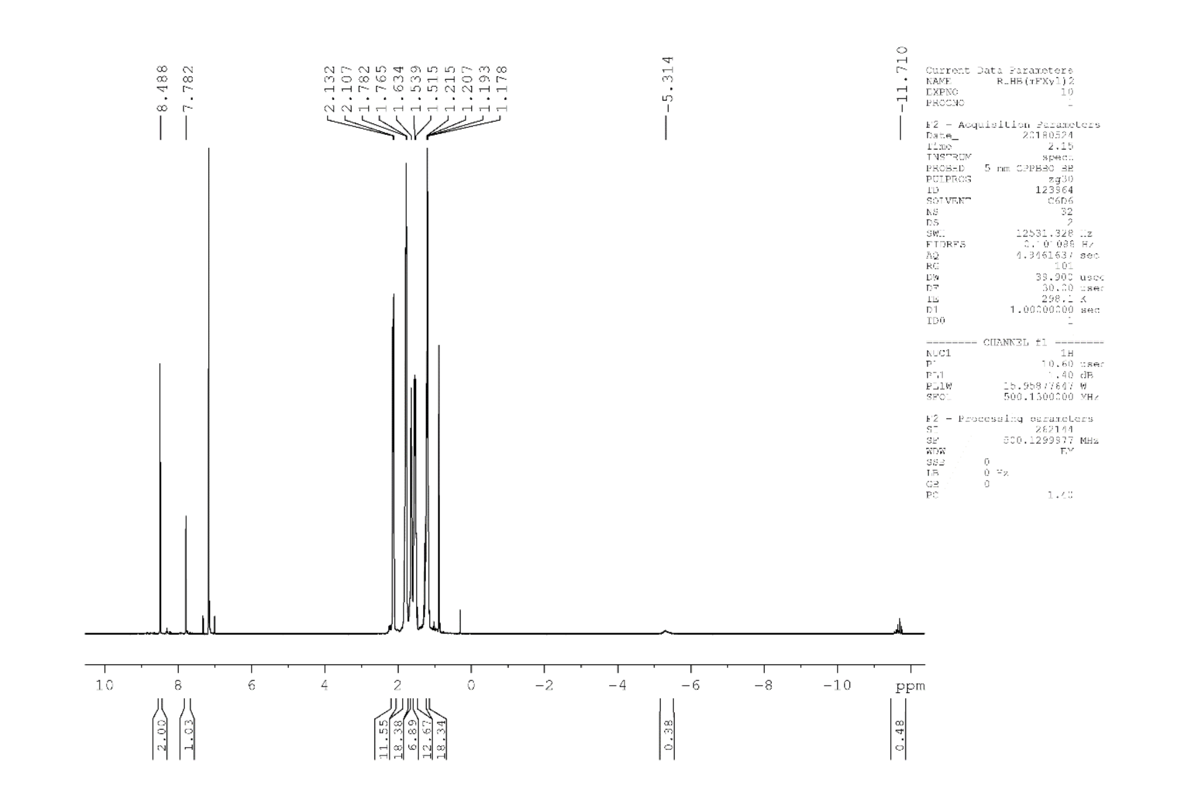

Figure S22: ^{1}H NMR spectrum of **[Ru(PCy$_3$)$_2$H$_2$(σ^2-H$_2$B‴FXyl)] (5)** in C_6D_6

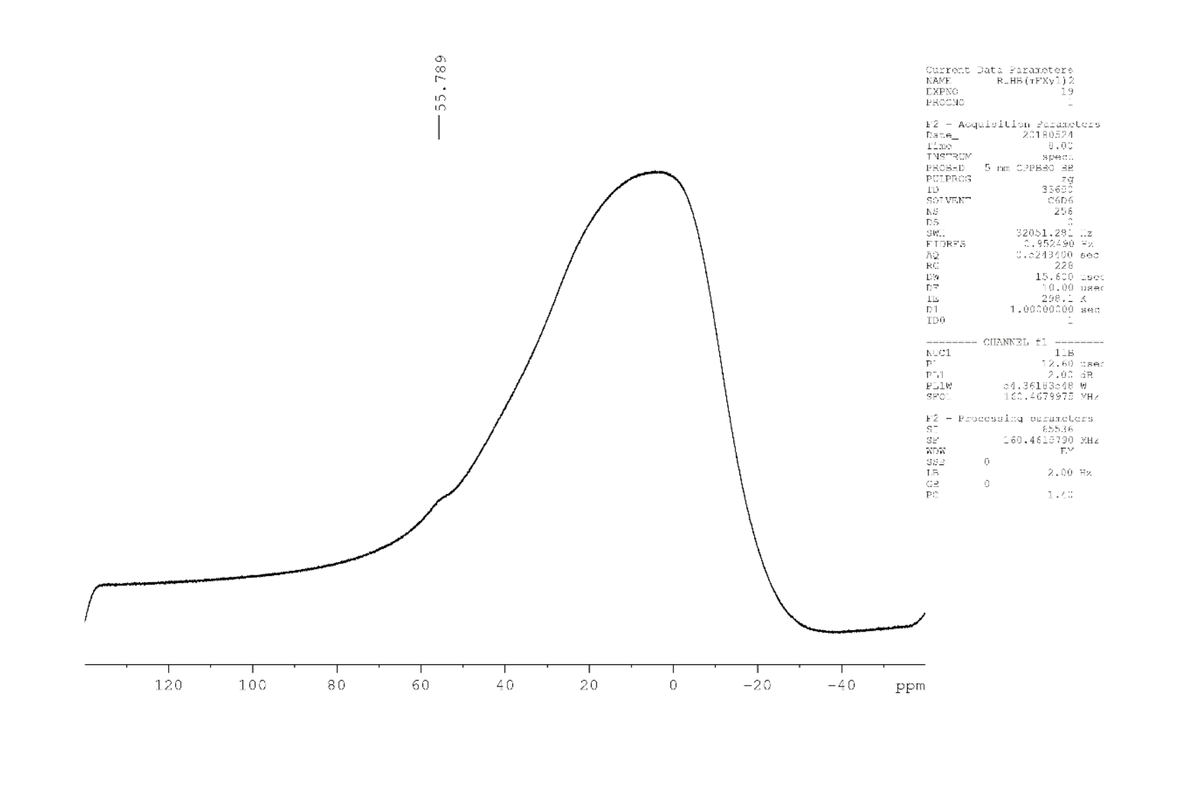

Figure S23: ^{11}B NMR spectrum of **[Ru(PCy$_3$)$_2$H$_2$(σ^2-H$_2$B‴FXyl)] (5)** in C_6D_6

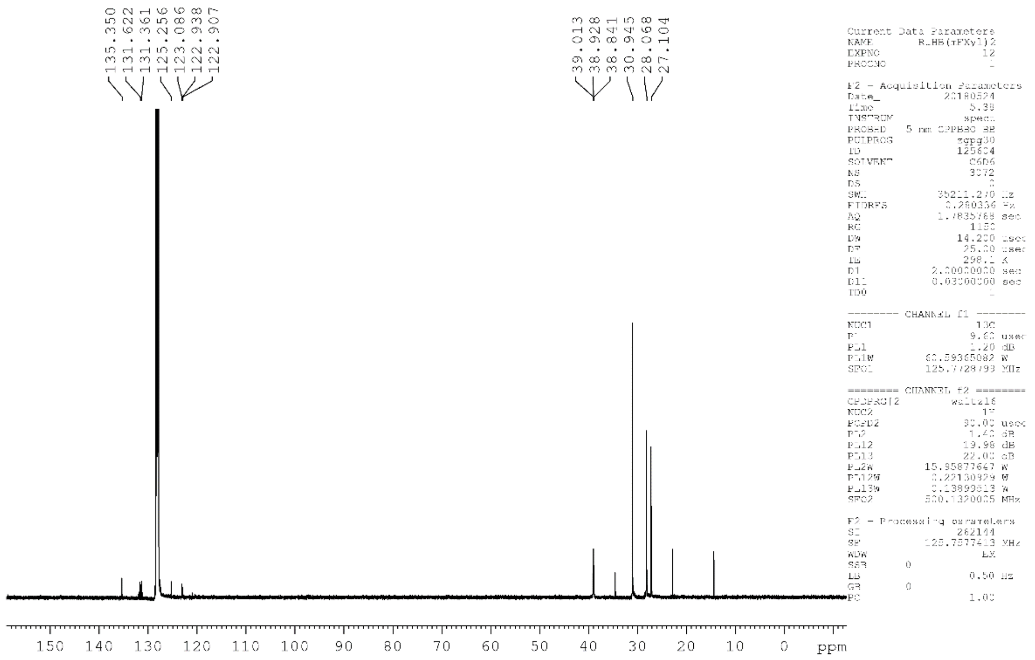

Figure S24: $^{13}C\{^{1}H\}$ NMR spectrum of **[Ru(PCy$_3$)$_2$H$_2$(σ^2-H$_2$B$'''$FXyl)] (5)** in C_6D_6

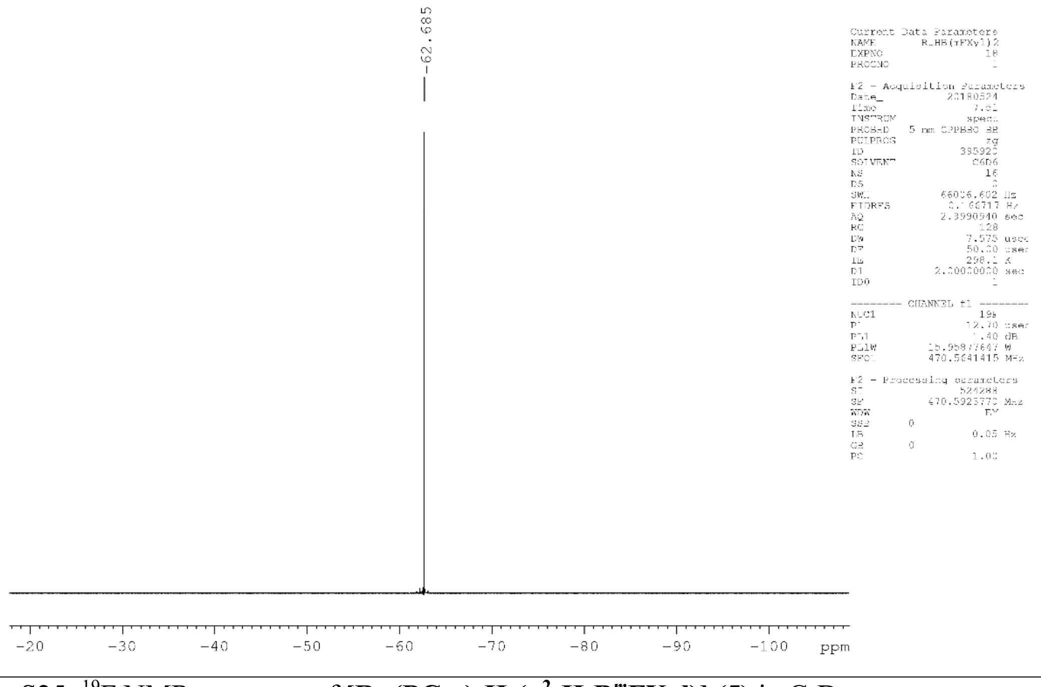

Figure S25: ^{19}F NMR spectrum of **[Ru(PCy$_3$)$_2$H$_2$(σ^2-H$_2$B$'''$FXyl)] (5)** in C_6D_6

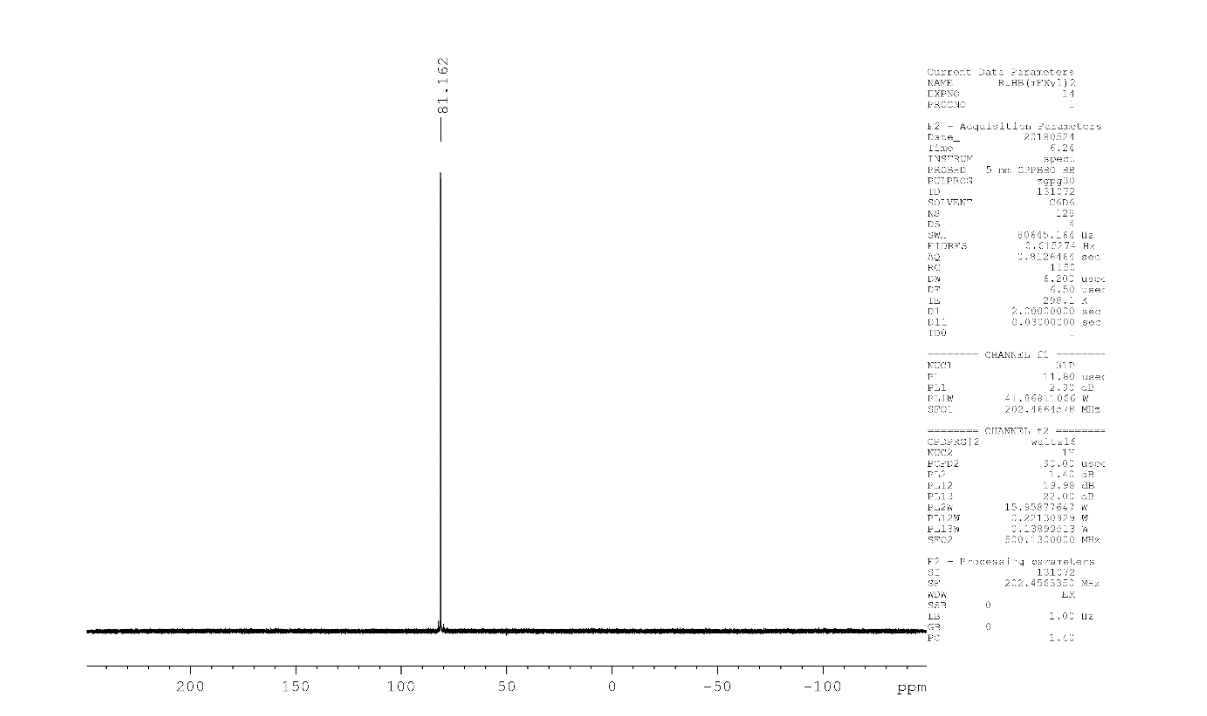

Figure S25: $^{31}P\{^1H\}$ NMR spectrum of **[Ru(PCy$_3$)$_2$H$_2$(σ^2-H$_2$B‴FXyl)] (5)** in C_6D_6

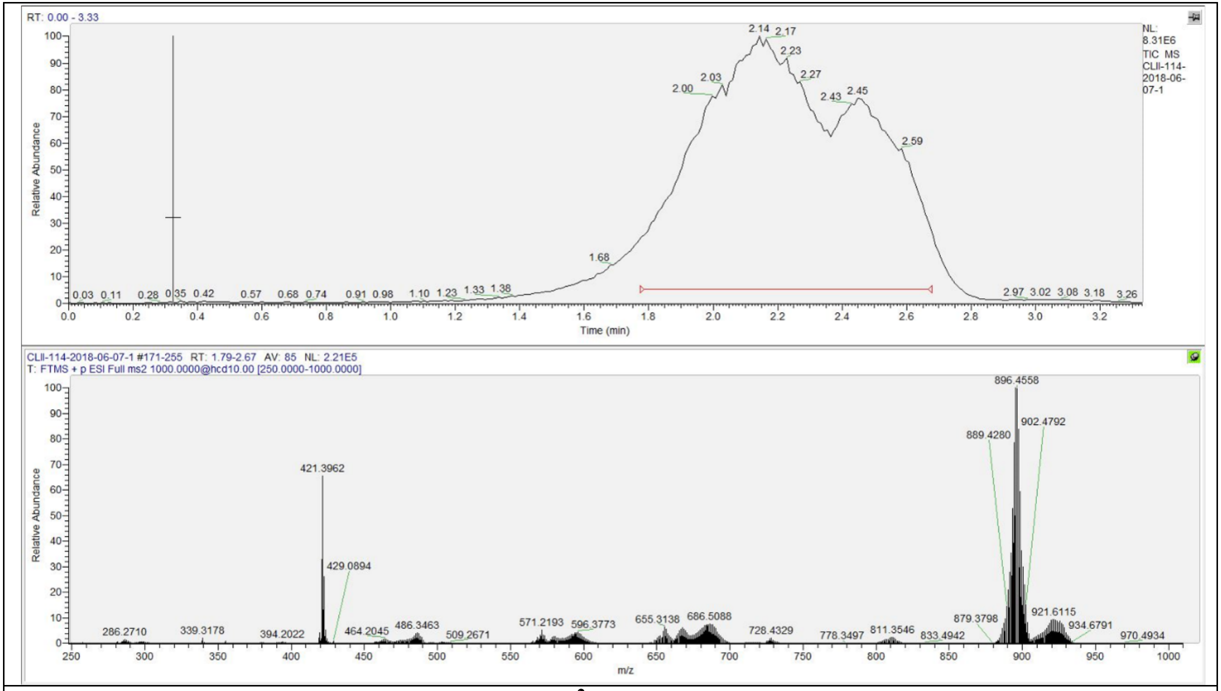

Figure S26: Mass spectrum of **[Ru(PCy$_3$)$_2$H$_2$(σ^2-H$_2$B‴FXyl)] (5)**

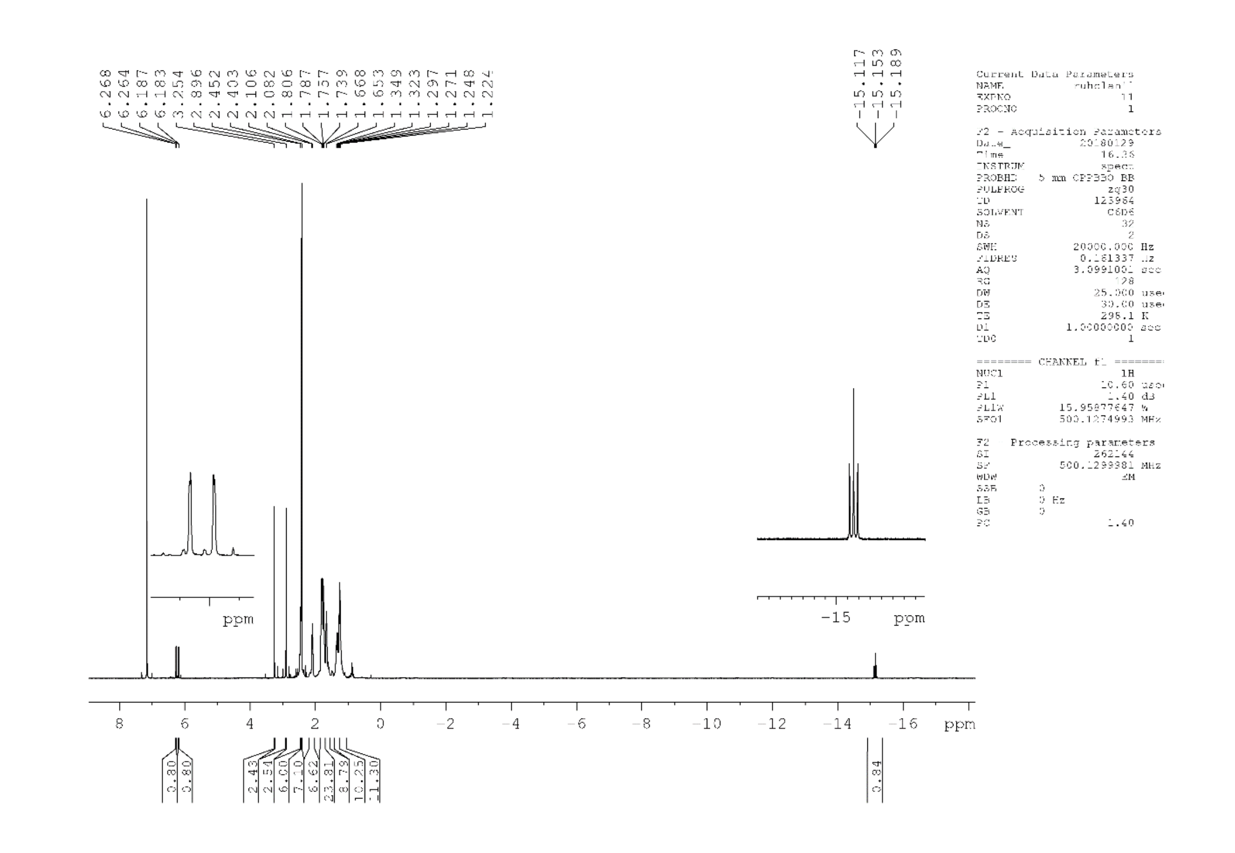

Figure S27: ^{1}H NMR spectrum of **[Ru(PCy$_3$)$_2$HCl(BAnil)] (6)** in C_6D_6

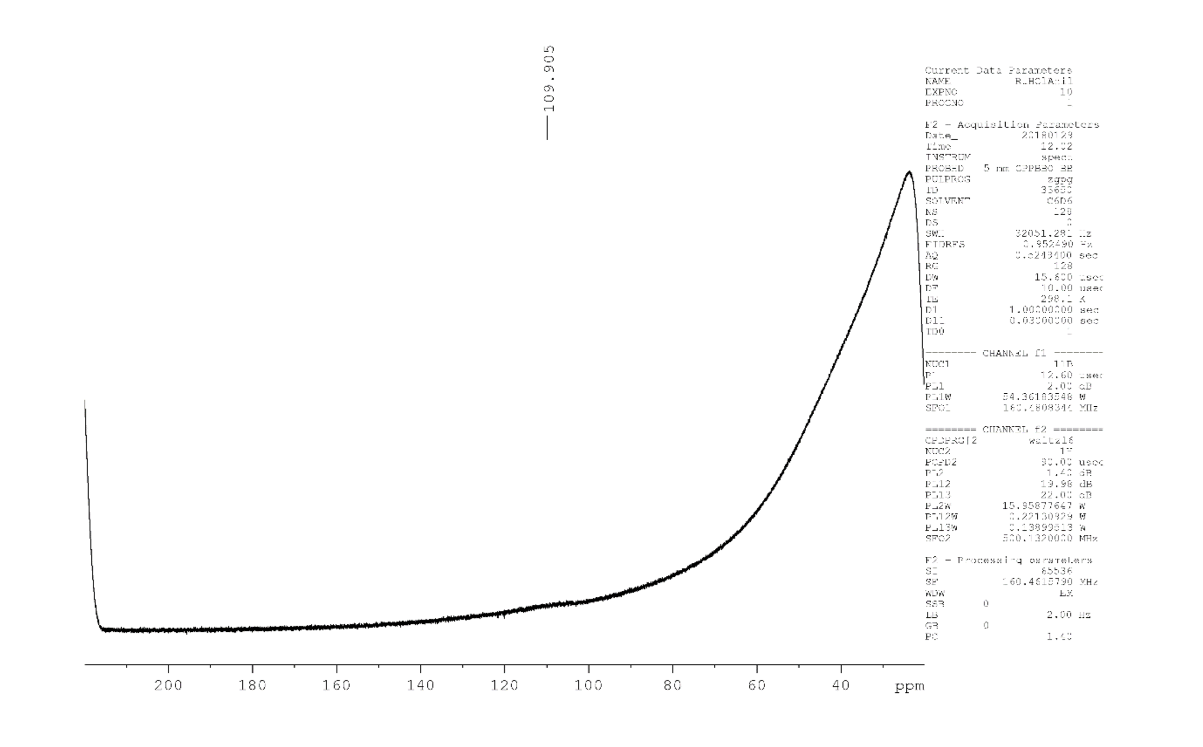

Figure S28: ^{11}B NMR spectrum of **[Ru(PCy$_3$)$_2$HCl(BAnil)] (6)** in C_6D_6

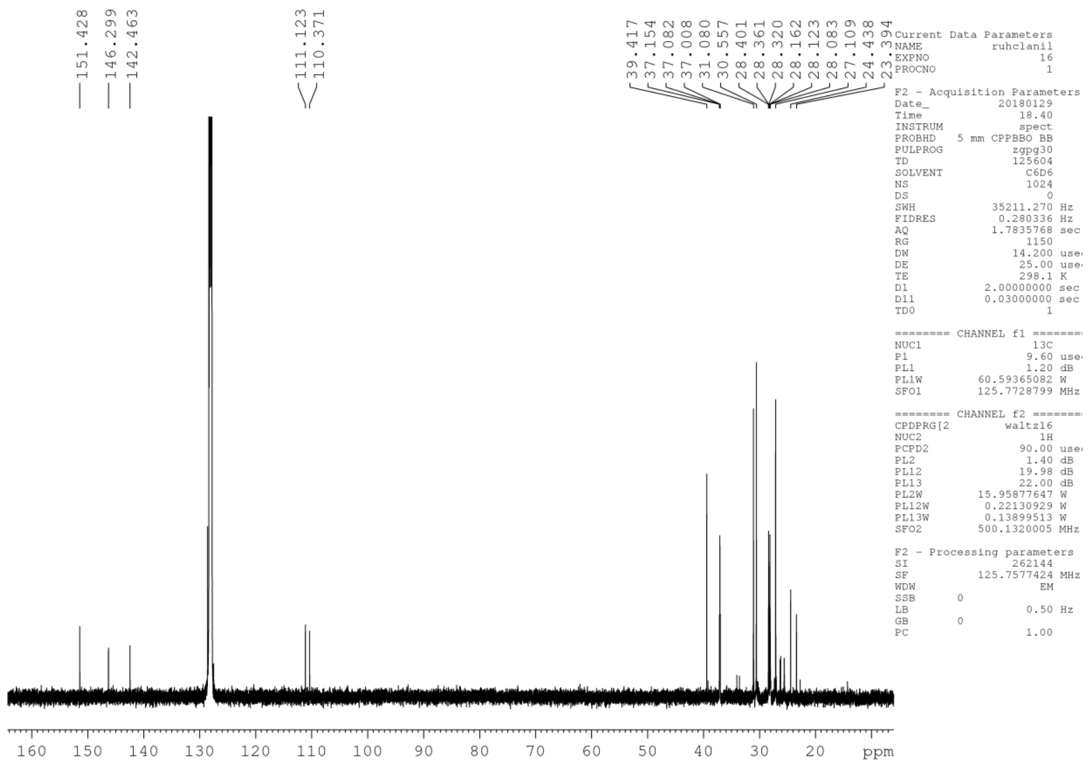

Figure S29: $^{13}C\{^{1}H\}$ NMR spectrum of **[Ru(PCy$_3$)$_2$HCl(BAnil)] (6)** in C_6D_6

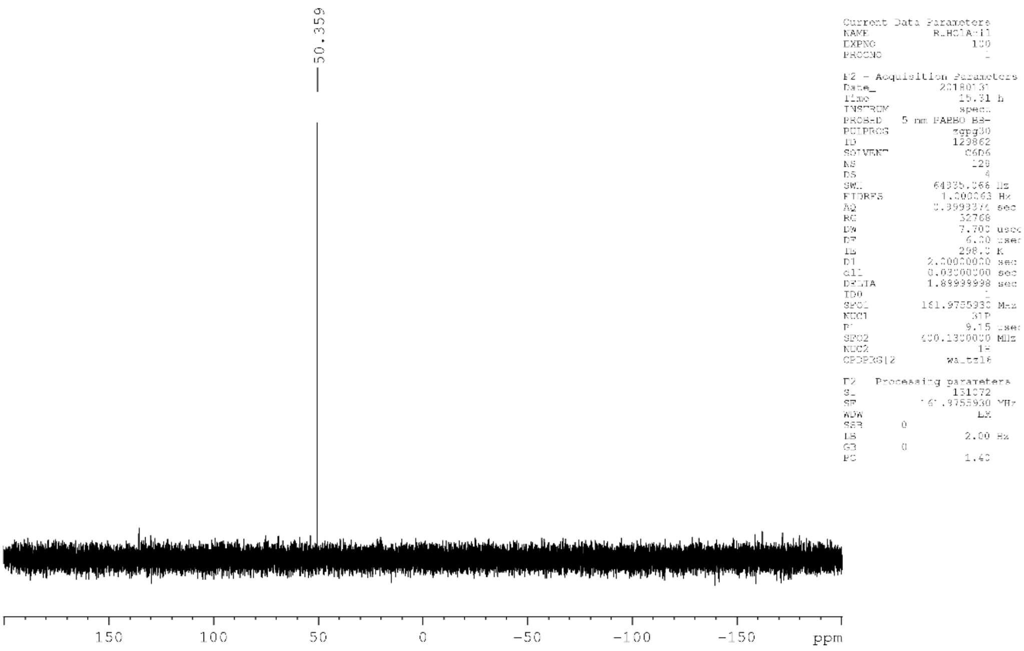

Figure S30: $^{31}P\{^{1}H\}$ NMR spectrum of **[Ru(PCy$_3$)$_2$HCl(BAnil)] (6)** in C_6D_6

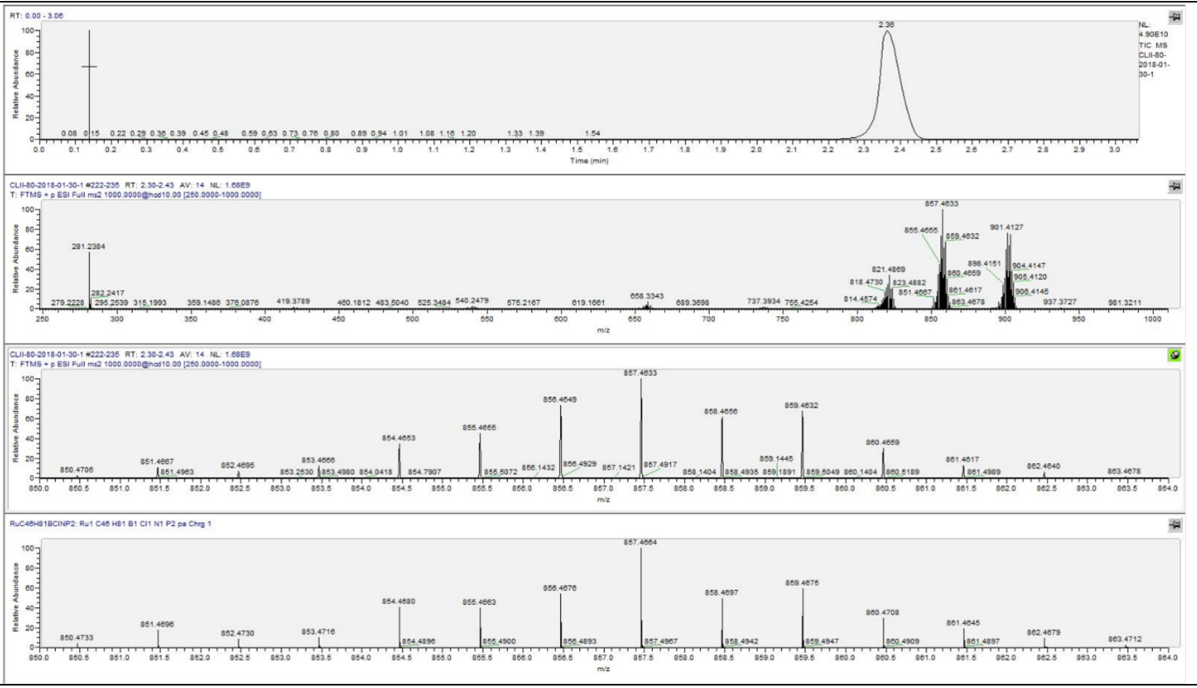

Figure S31: Mass spectrum of **[Ru(PCy$_3$)$_2$HCl(BAnil)] (6)**

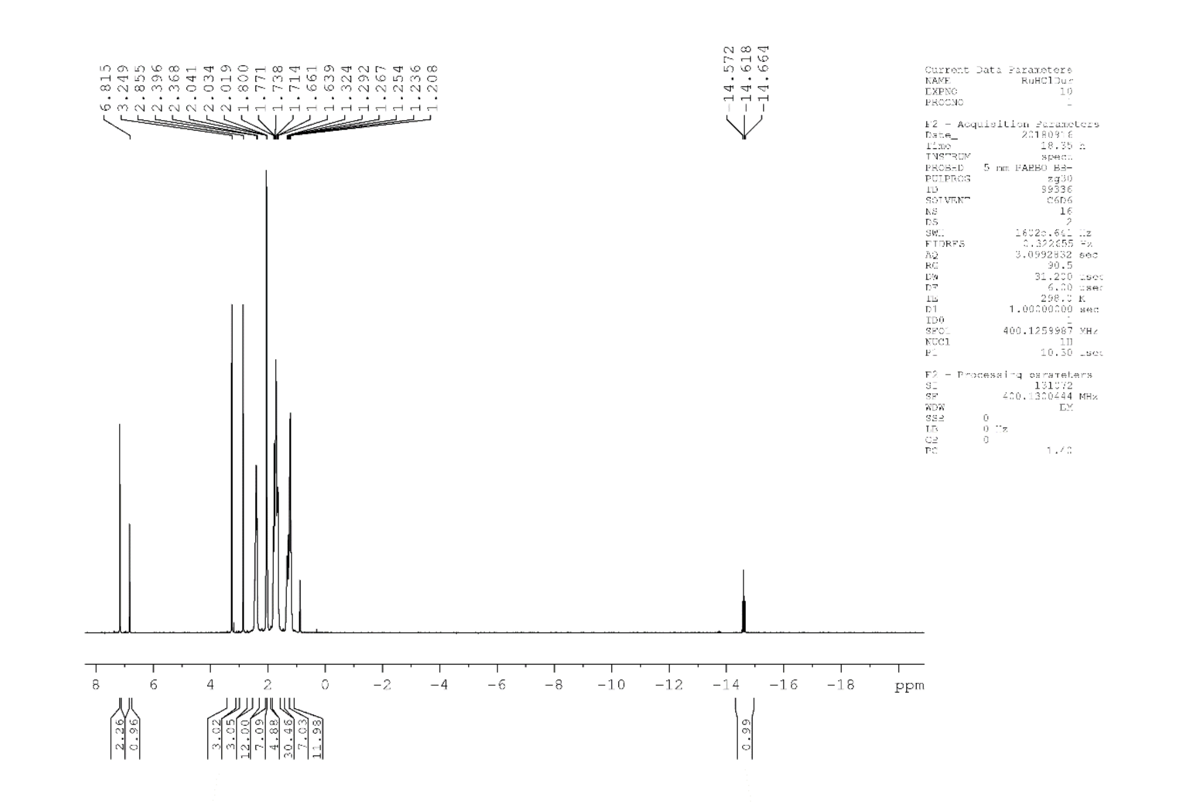

Figure S32: ^{1}H NMR spectrum of **[Ru(PCy$_3$)$_2$HCl(BDur)] (7)** in C_6D_6

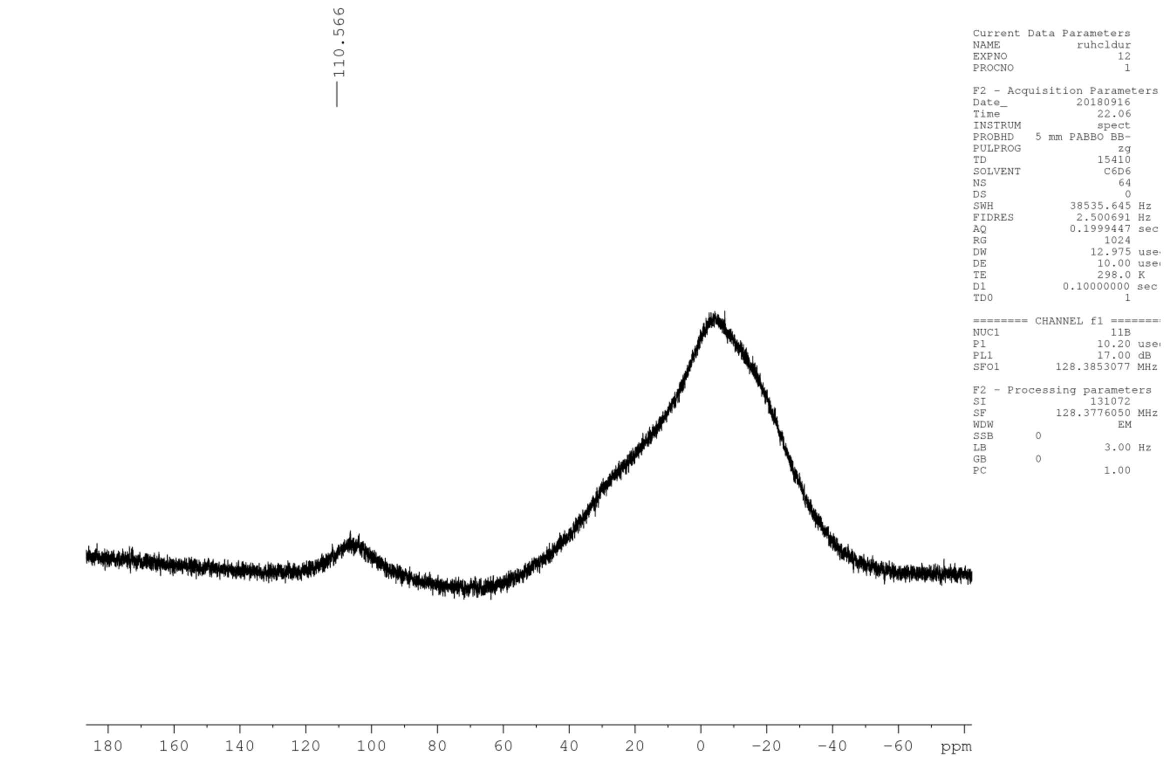

Figure S33: ^{11}B NMR spectrum of **[Ru(PCy$_3$)$_2$HCl(BDur)] (7)** in C_6D_6

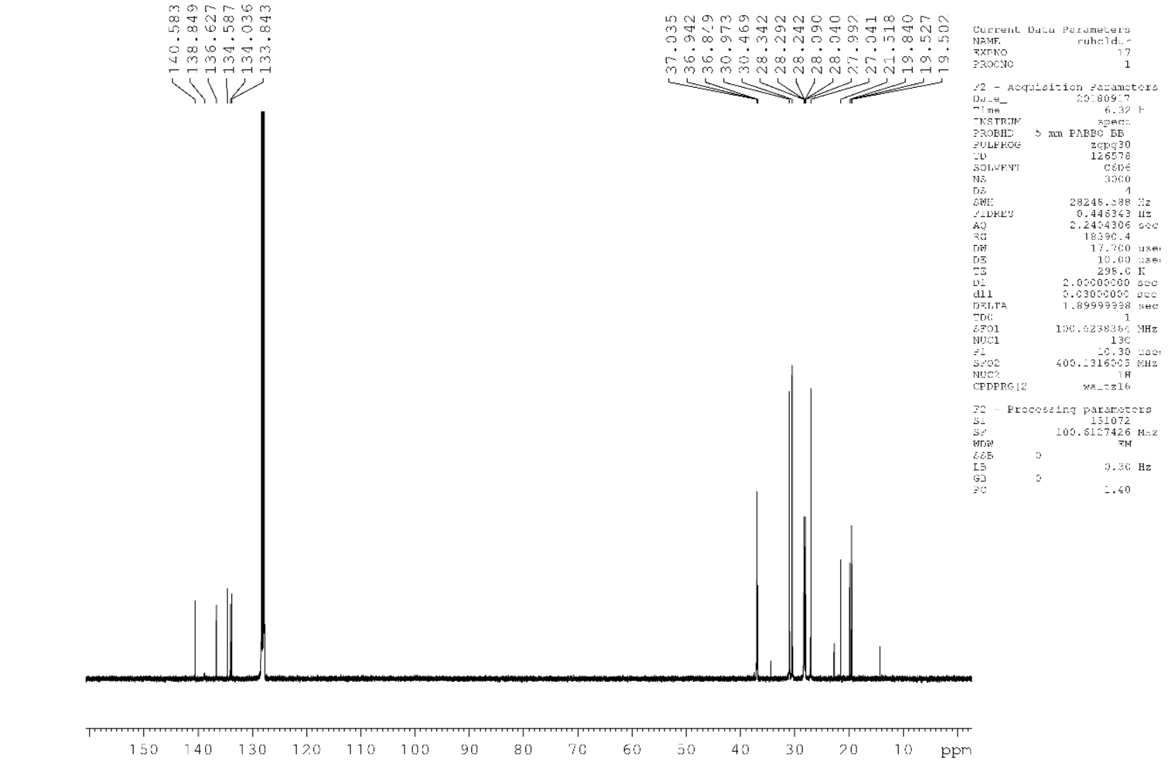

Figure S34: $^{13}C\{^{1}H\}$ NMR spectrum of **[Ru(PCy$_3$)$_2$HCl(BDur)] (7)** in C_6D_6

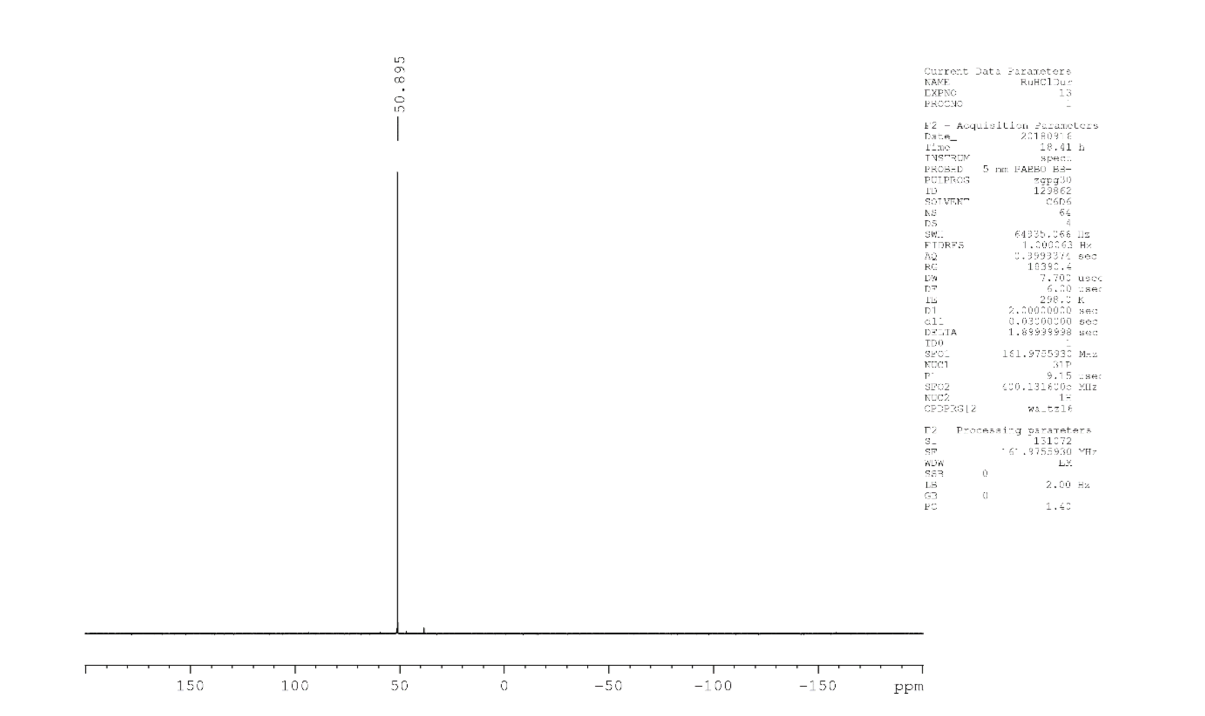

Figure S35: $^{31}P\{^{1}H\}$ NMR spectrum of **[Ru(PCy$_3$)$_2$HCl(BDur)] (7)** in C_6D_6

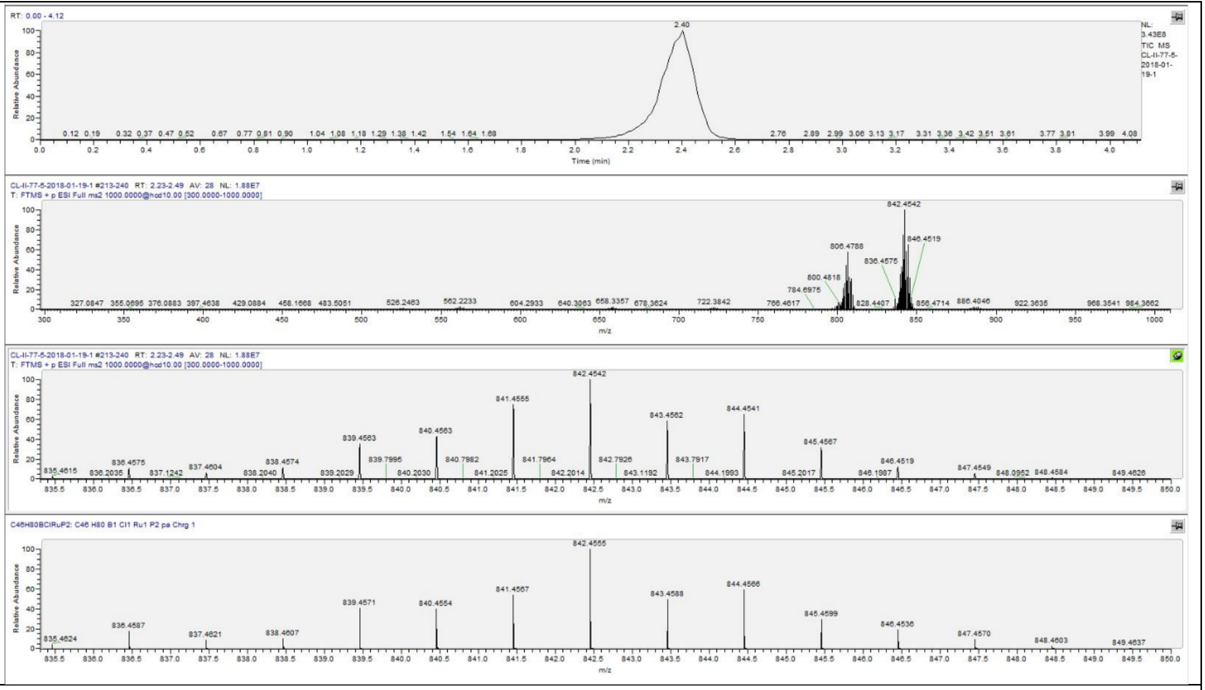

Figure S36: Mass spectrum of **[Ru(PCy$_3$)$_2$HCl(BDur)] (7)**

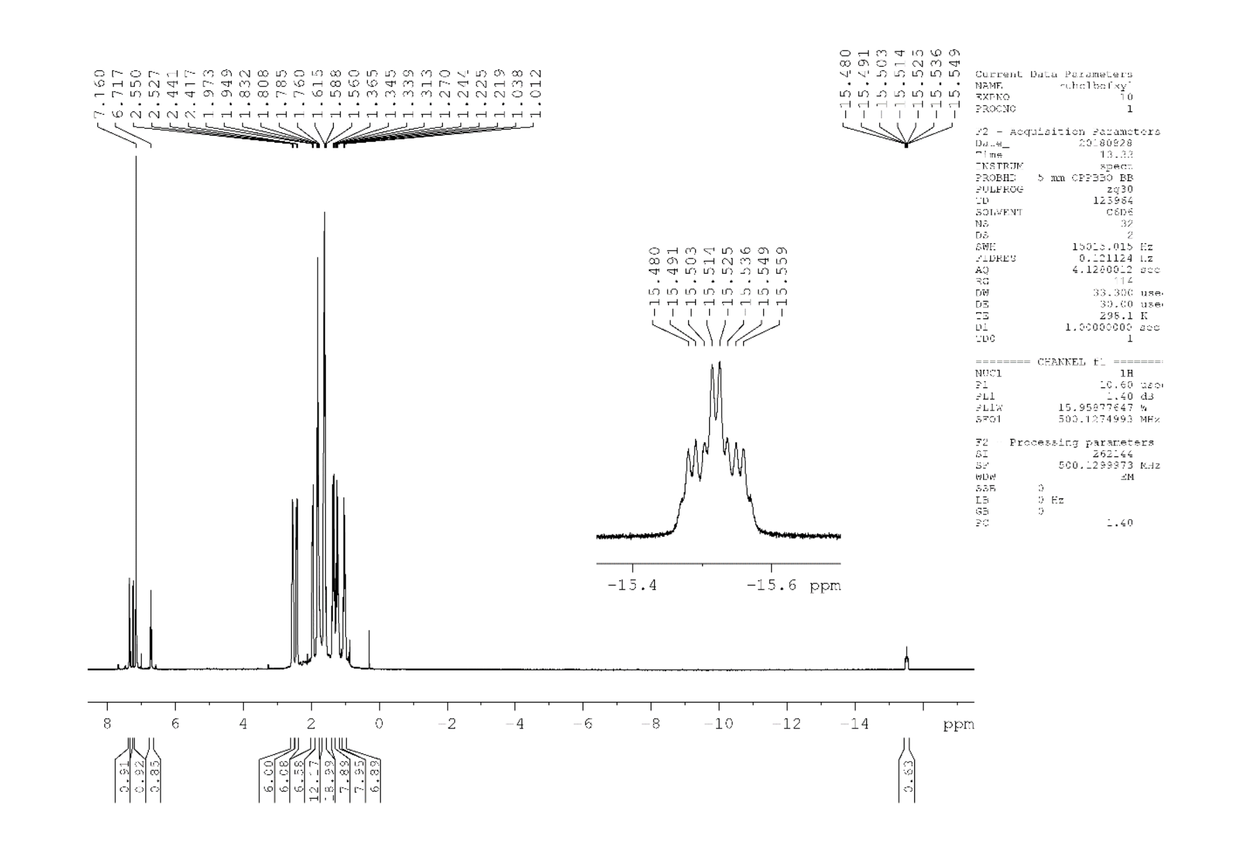

Figure S37: ^{1}H NMR spectrum of **[Ru(PCy$_3$)$_2$HCl(B^{o}FXyl)] (8)** in C_6D_6

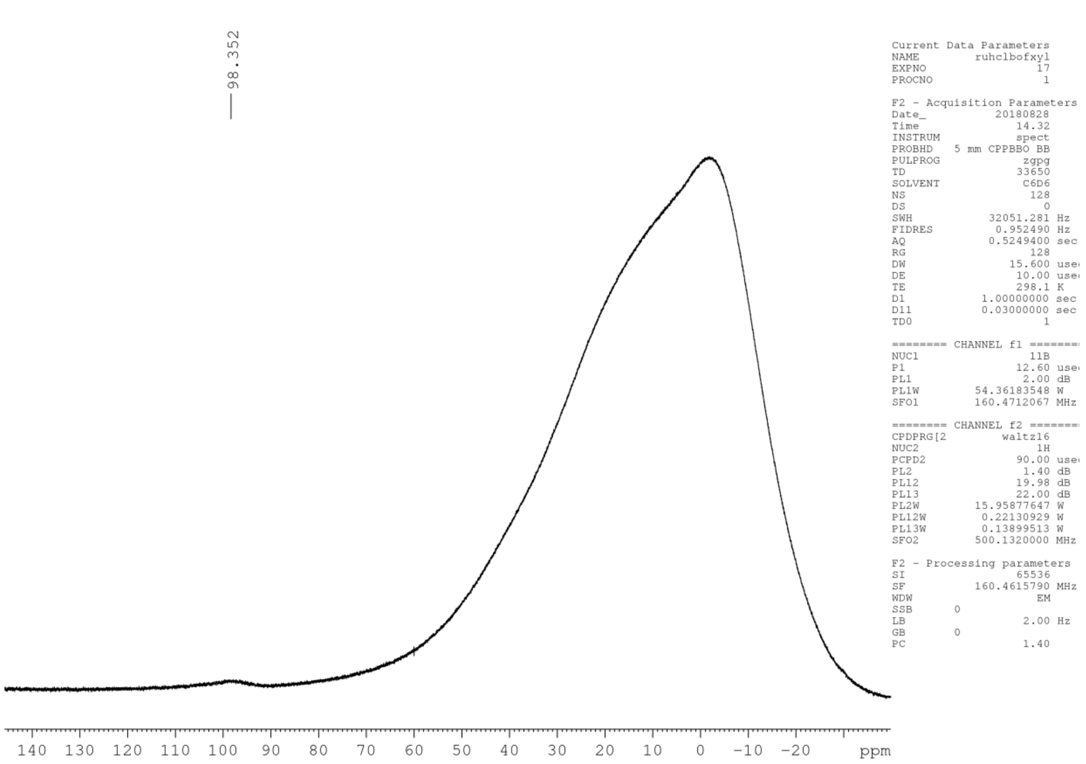

Figure S38: ^{11}B NMR spectrum of **[Ru(PCy$_3$)$_2$HCl(B^{o}FXyl)] (8)** in C_6D_6

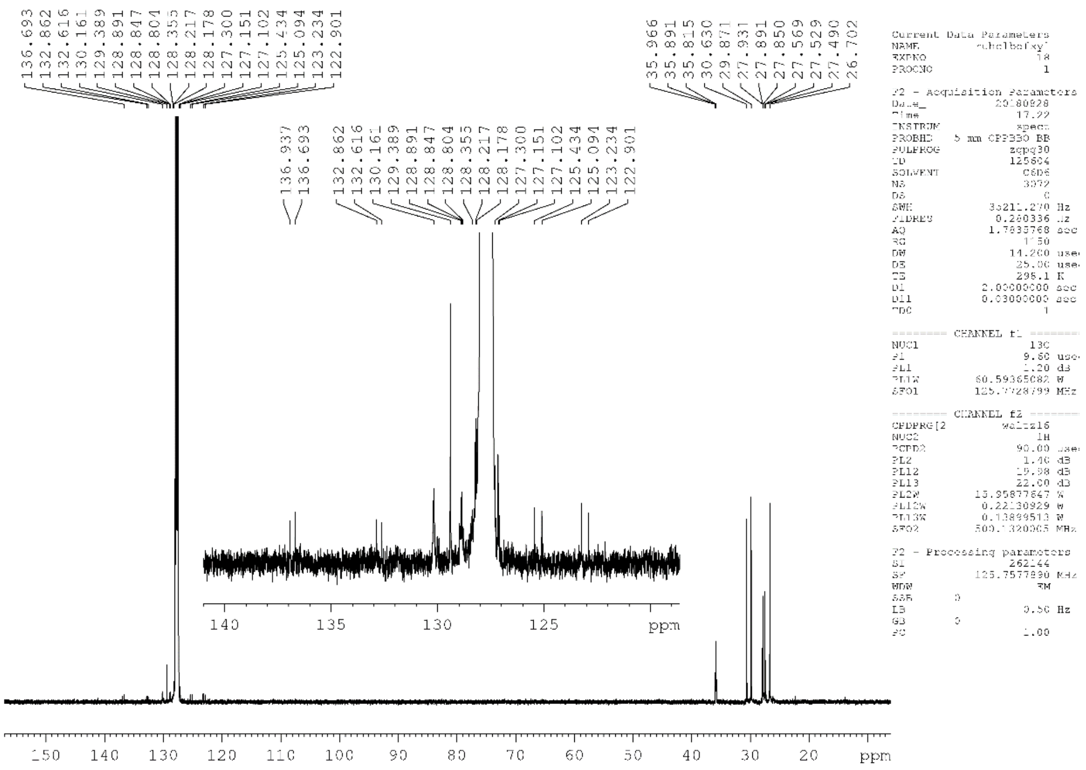

Figure S39: $^{13}C\{^{1}H\}$ NMR spectrum of **[Ru(PCy$_3$)$_2$HCl(B^{o}FXyl)] (8)** in C_6D_6

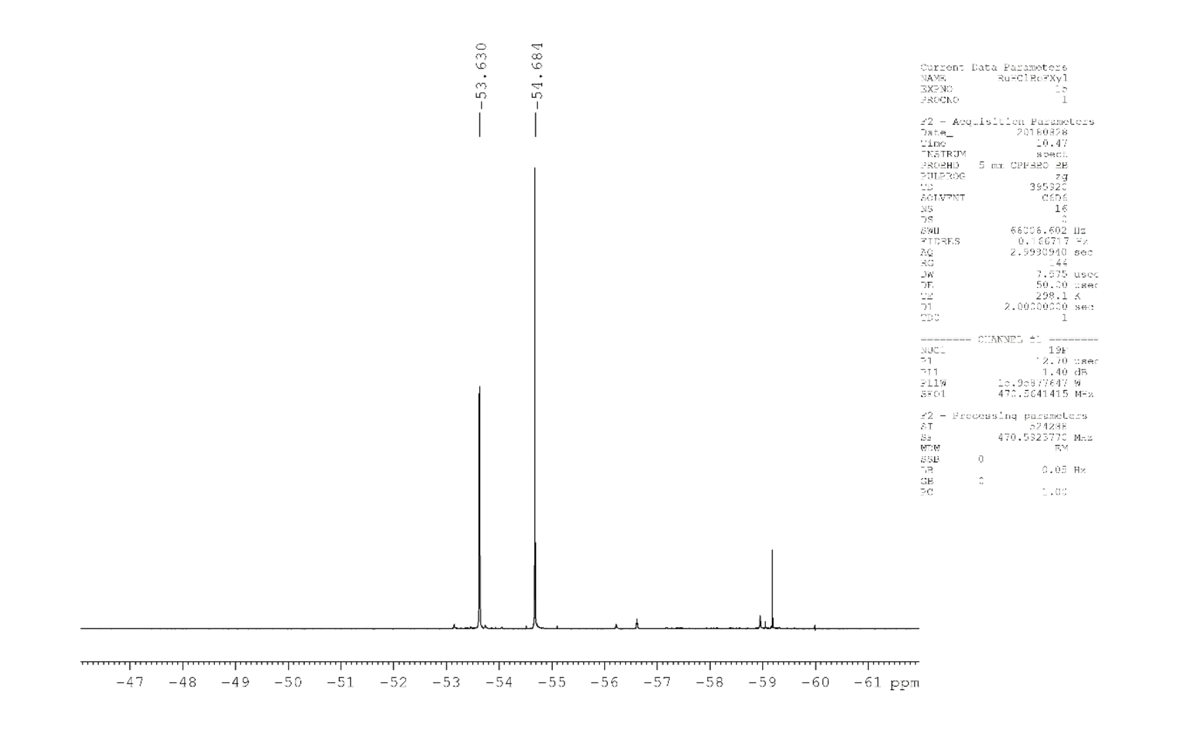

Figure S40: ^{19}F NMR spectrum of **[Ru(PCy$_3$)$_2$HCl(B^{o}FXyl)] (8)** in C_6D_6

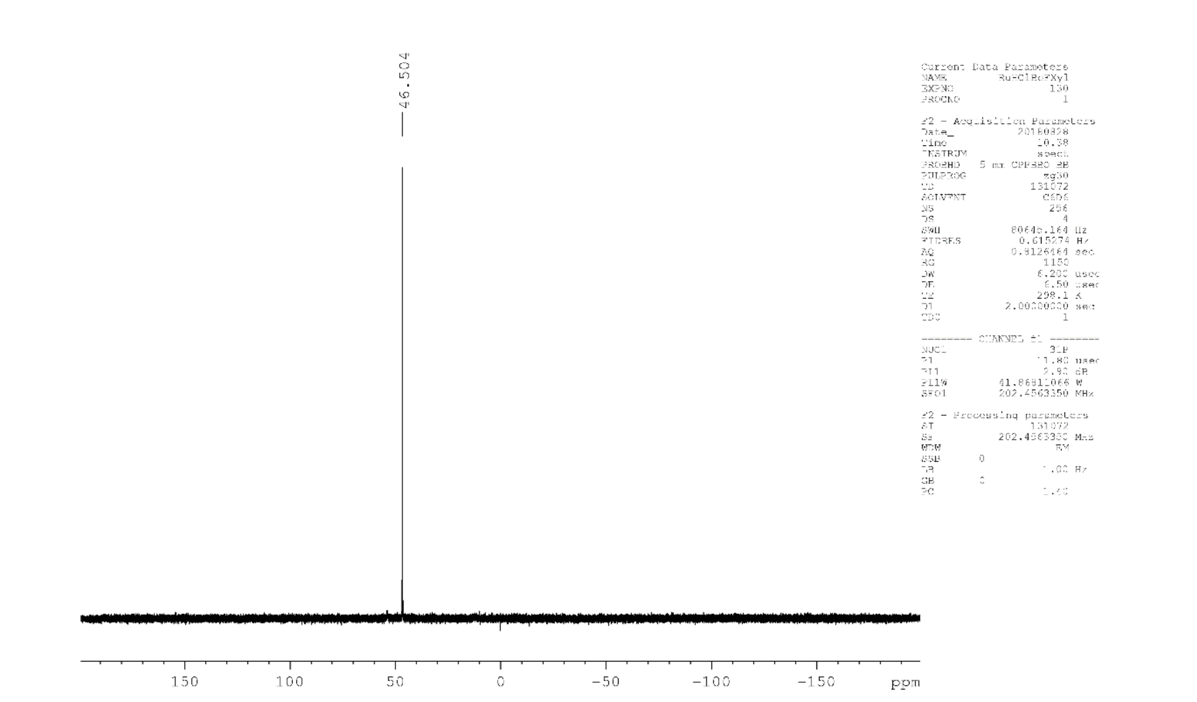

Figure S41: $^{31}P\{^{1}H\}$ NMR spectrum of **[Ru(PCy$_3$)$_2$HCl(B^{o}FXyl)] (8)** in C_6D_6

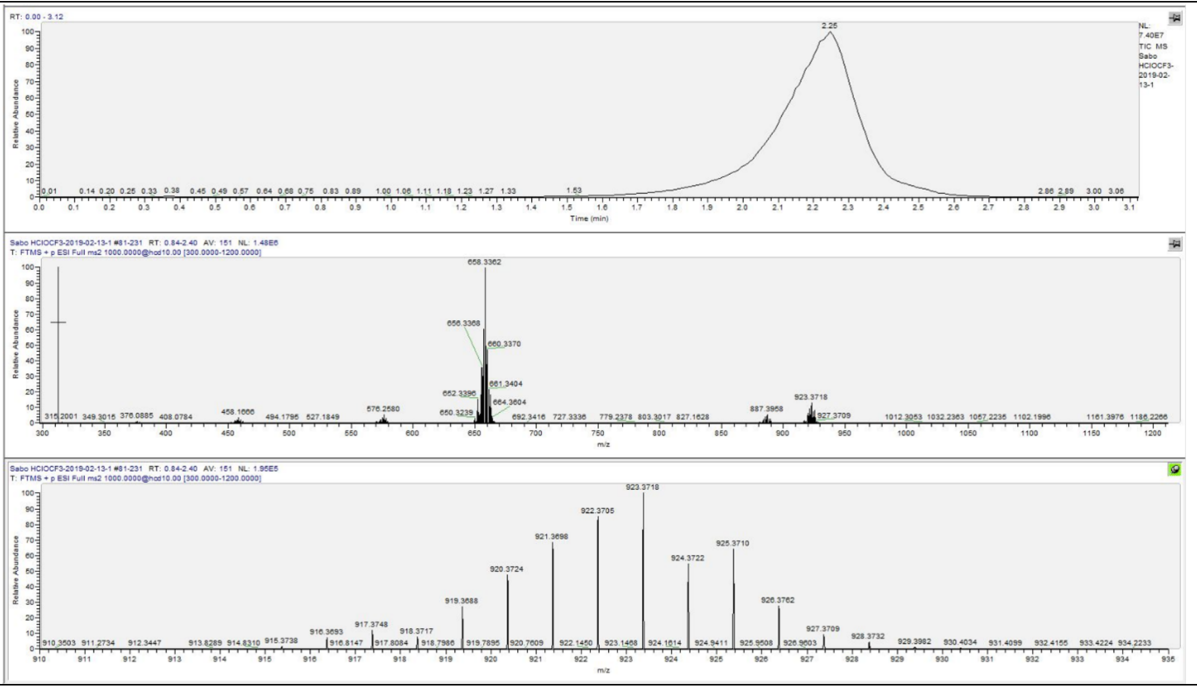

Figure S42: Mass spectrum of **[Ru(PCy$_3$)$_2$HCl(B^{o}FXyl)] (8)**

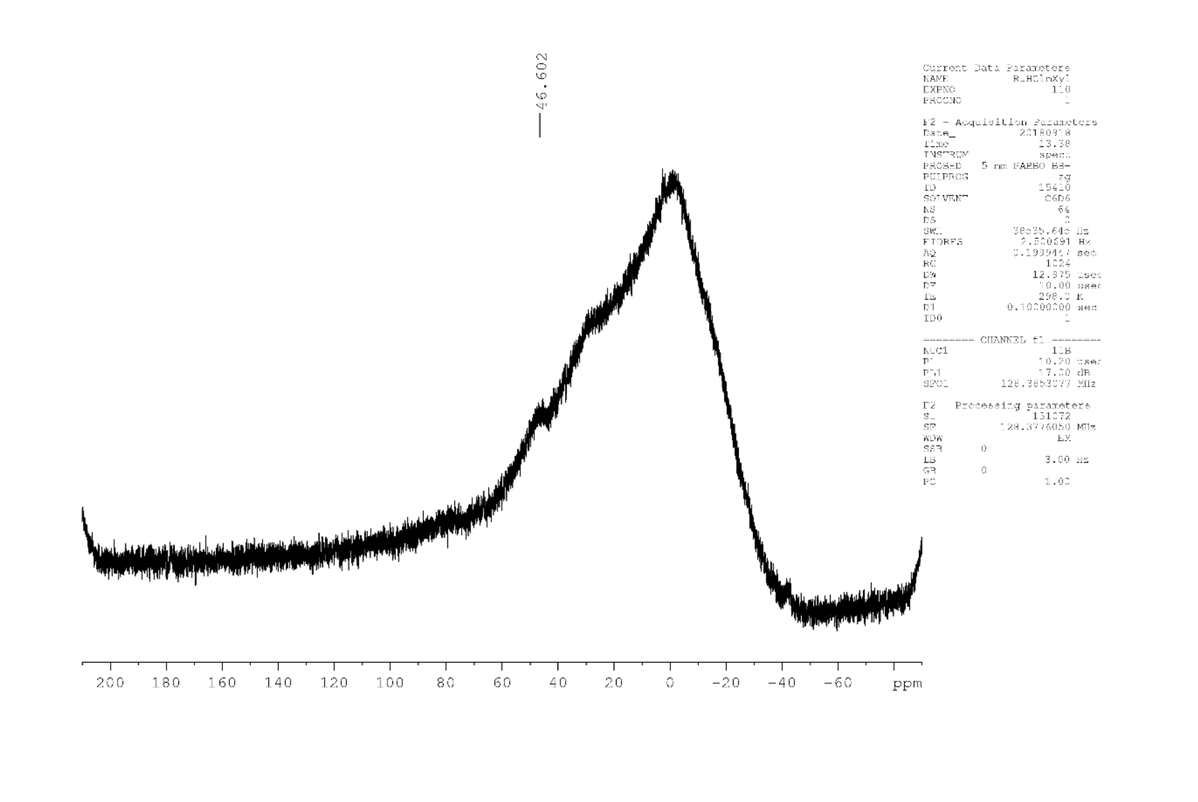

Figure S43: ^{11}B NMR spectrum of reaction mixture for synthesis of **[Ru(PCy$_3$)$_2$HCl(B$''$Xyl)]** in C_6D_6

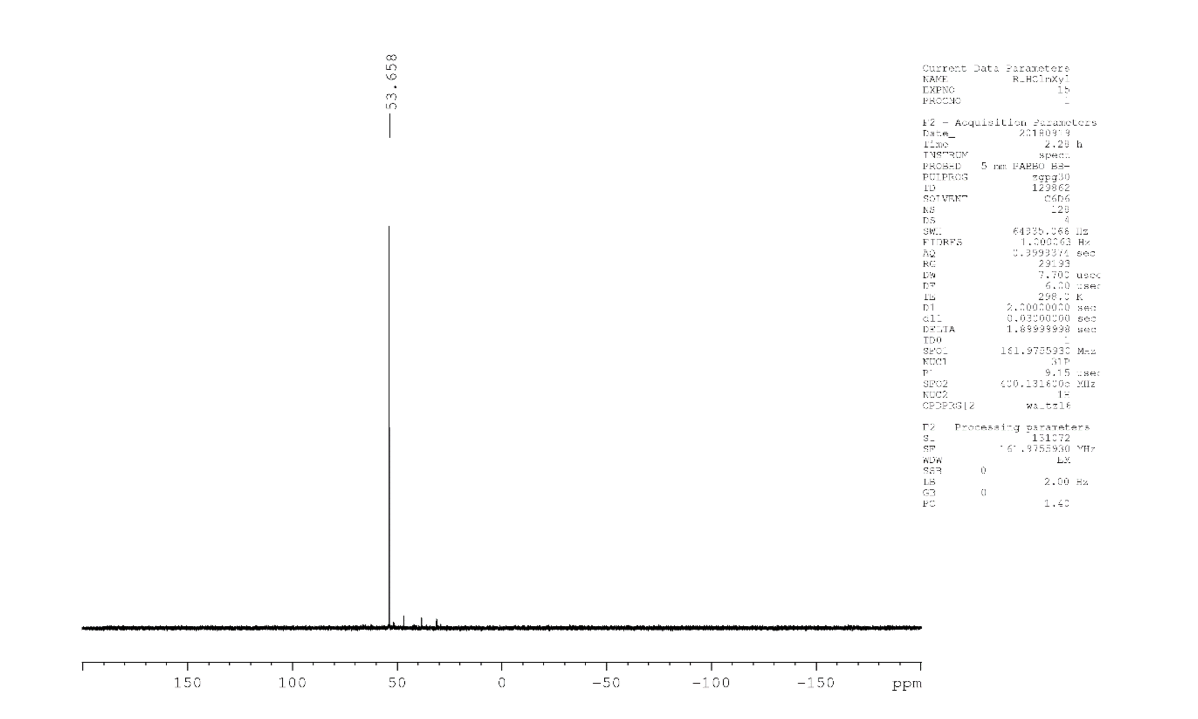

Figure S44: $^{31}P\{^{1}H\}$ NMR spectrum of reaction mixture for synthesis of **[Ru(PCy$_3$)$_2$HCl(B‴Xyl)]** in C_6D_6

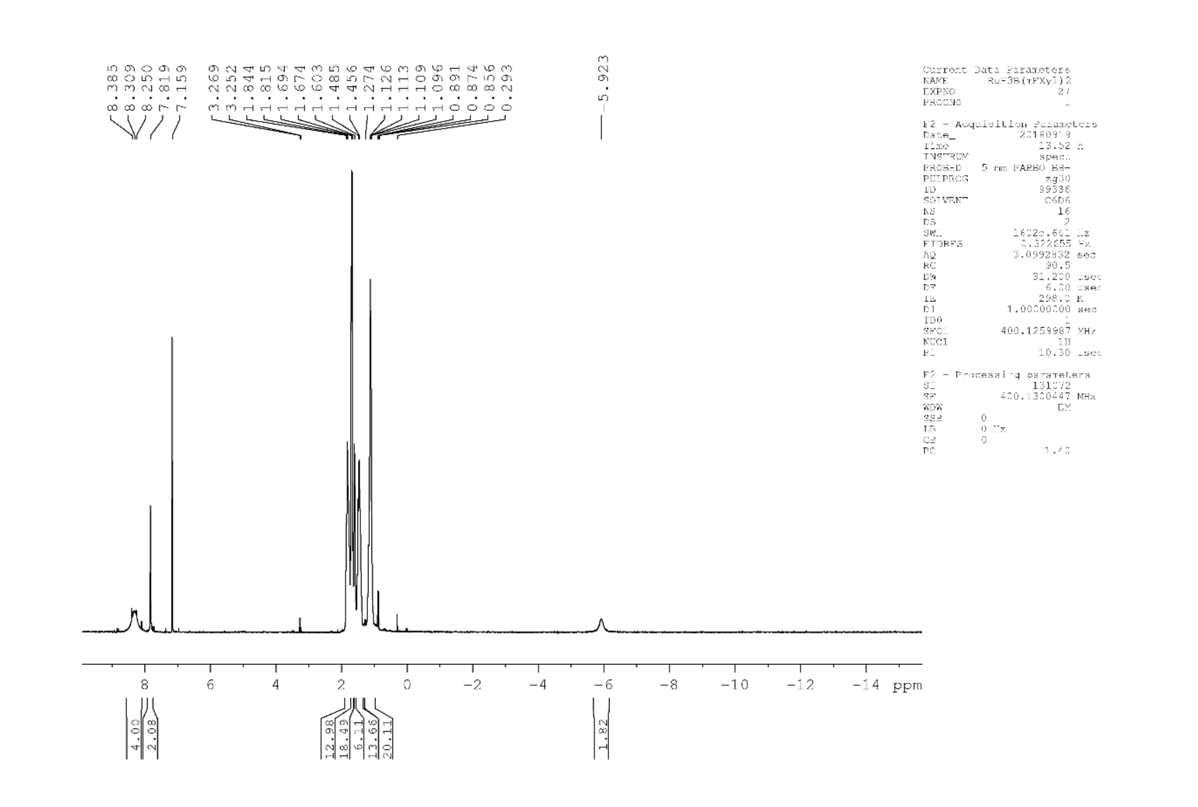

Figure S45: ^{1}H NMR spectrum of **[Ru(PCy$_3$)$_2$H(σ^2-H$_2$B{‴FXyl}$_2$)] (10)** in C_6D_6

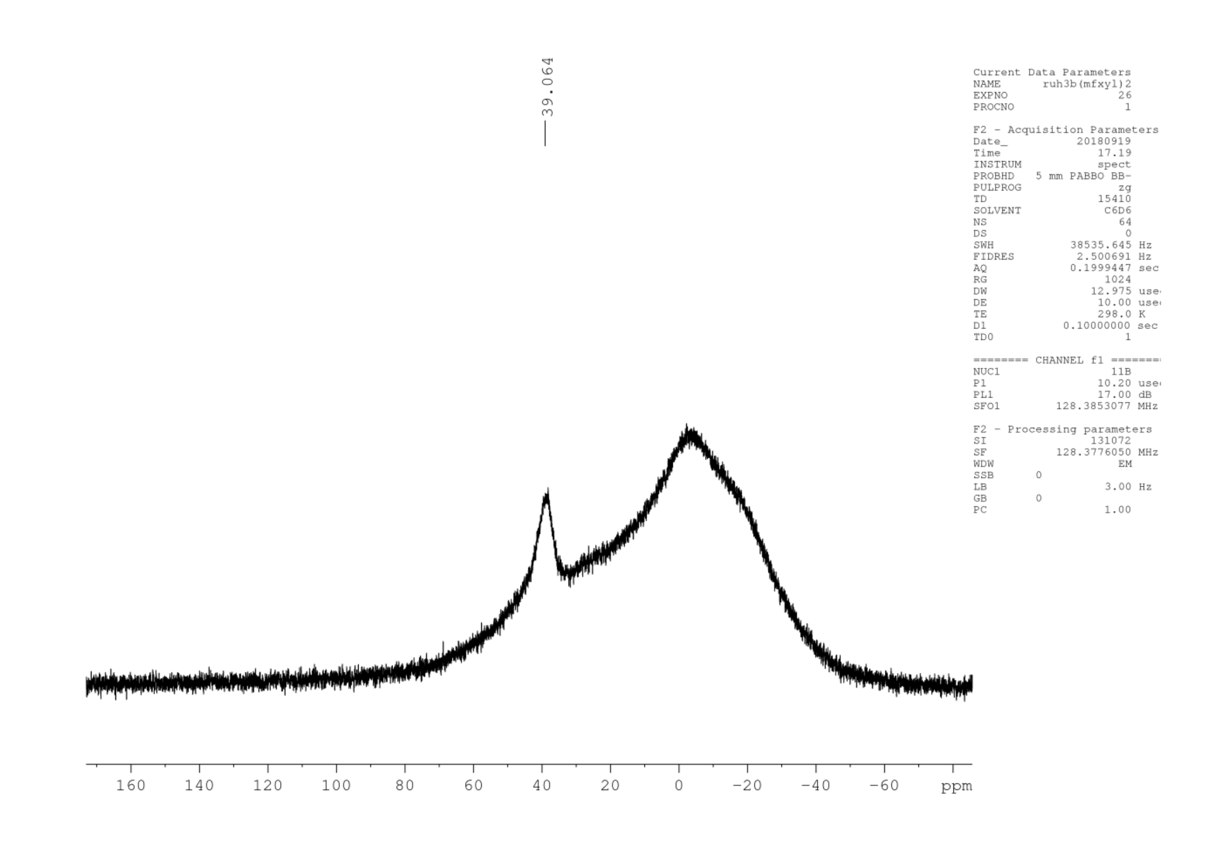

Figure S46: ^{11}B NMR spectrum of **[Ru(PCy$_3$)$_2$H(σ^2-H$_2$B{^{m}FXyl}$_2$)] (10)** in C_6D_6

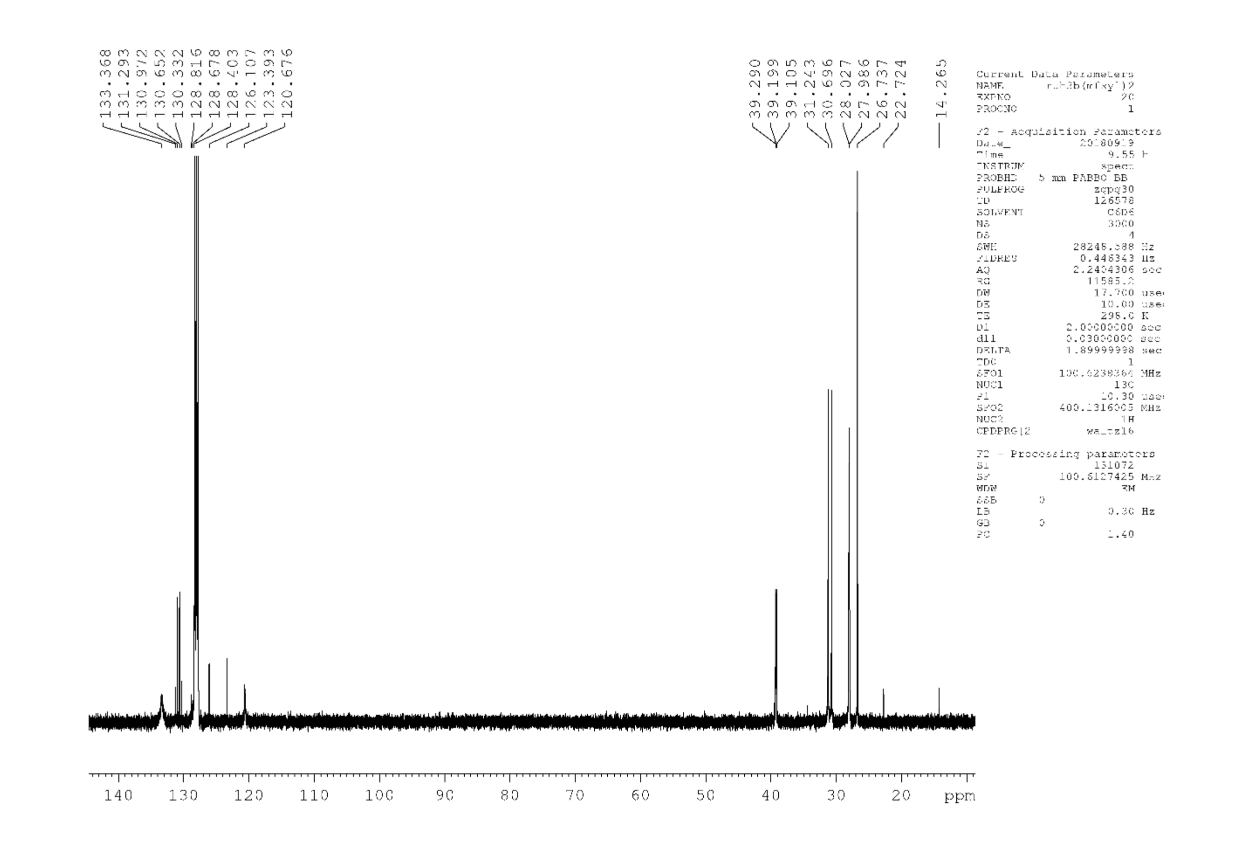

Figure S47: $^{13}C\{^1H\}$ NMR spectrum of **[Ru(PCy$_3$)$_2$H(σ^2-H$_2$B{^{m}FXyl}$_2$)] (10)** in C_6D_6

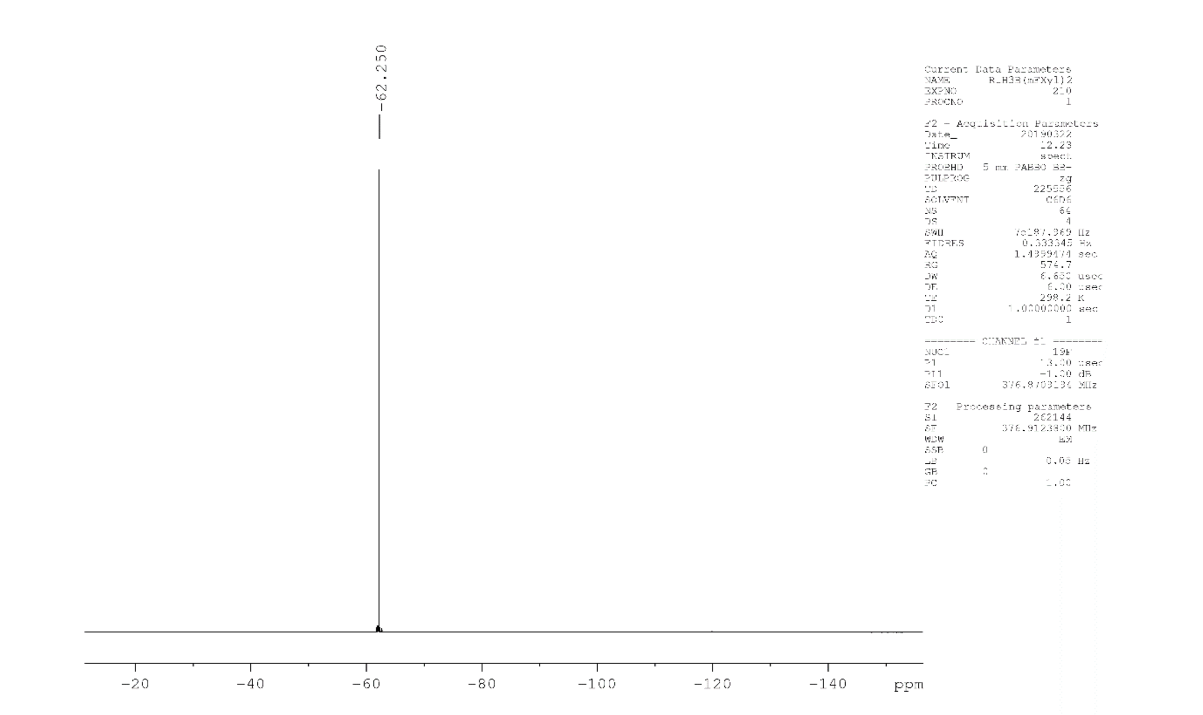

Figure S48: ^{19}F NMR spectrum of **[Ru(PCy$_3$)$_2$H(σ^2-H$_2$B{^{m}FXyl}$_2$)] (10)** in C_6D_6

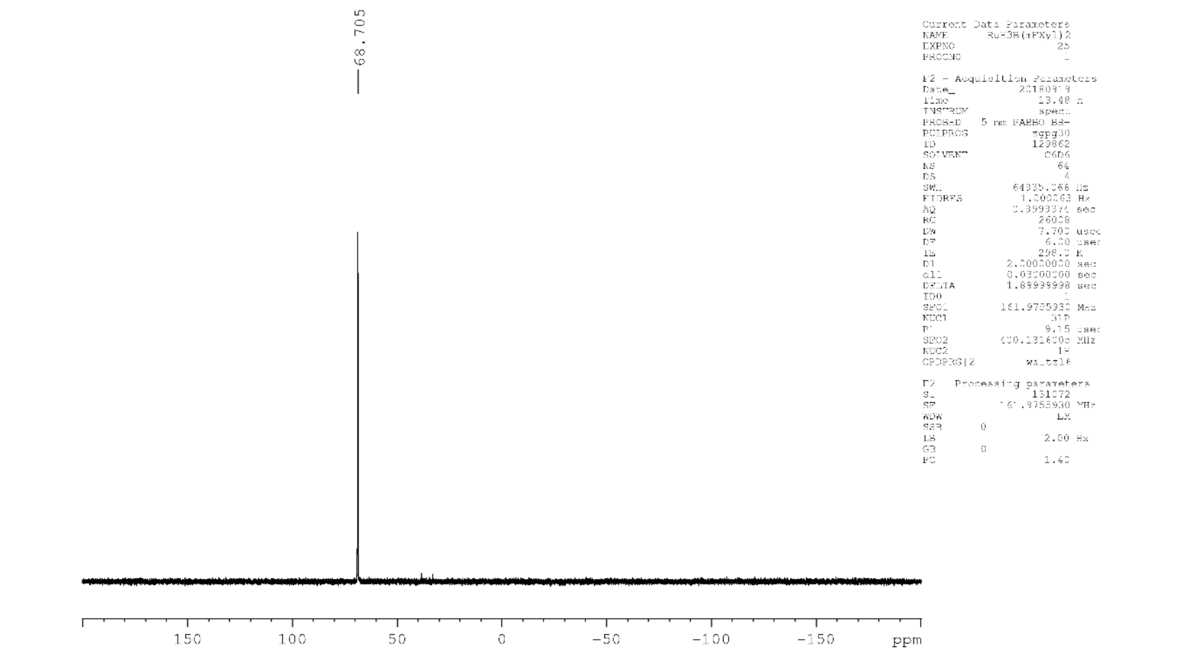

Figure S49: $^{31}P\{^{1}H\}$ NMR spectrum of **[Ru(PCy$_3$)$_2$H(σ^2-H$_2$B{‴FXyl}$_2$)] (10)** in C_6D_6

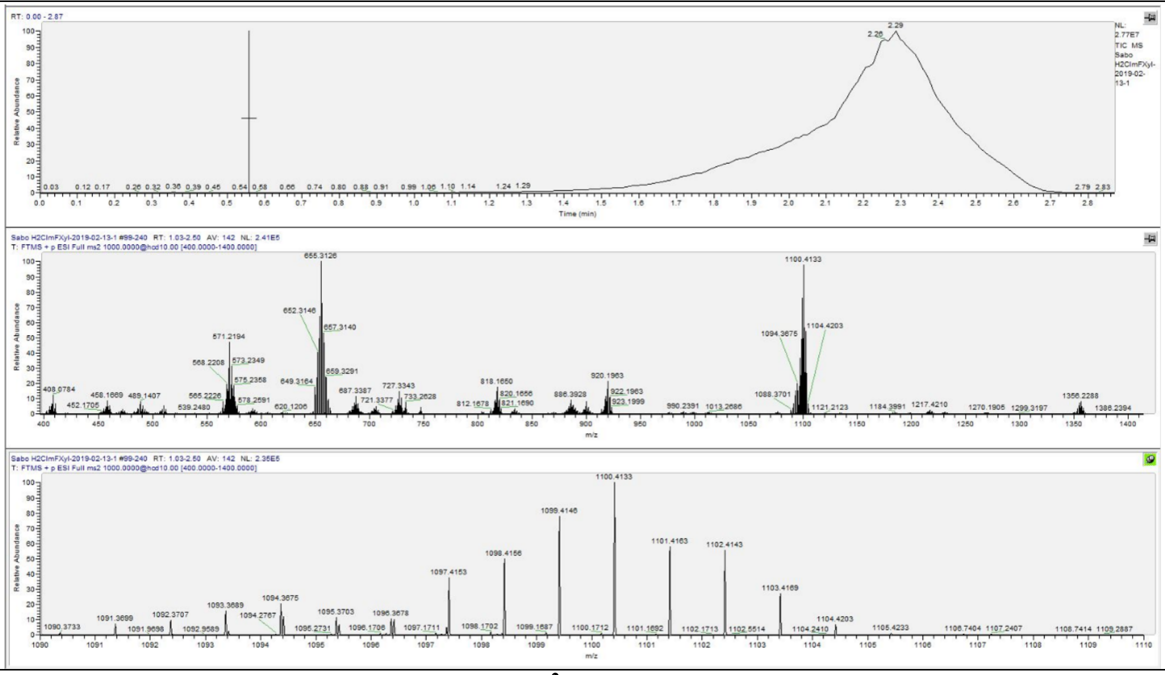

Figure S50: Mass spectrum of **[Ru(PCy$_3$)$_2$H(σ^2-H$_2$B{‴FXyl}$_2$)] (10)**

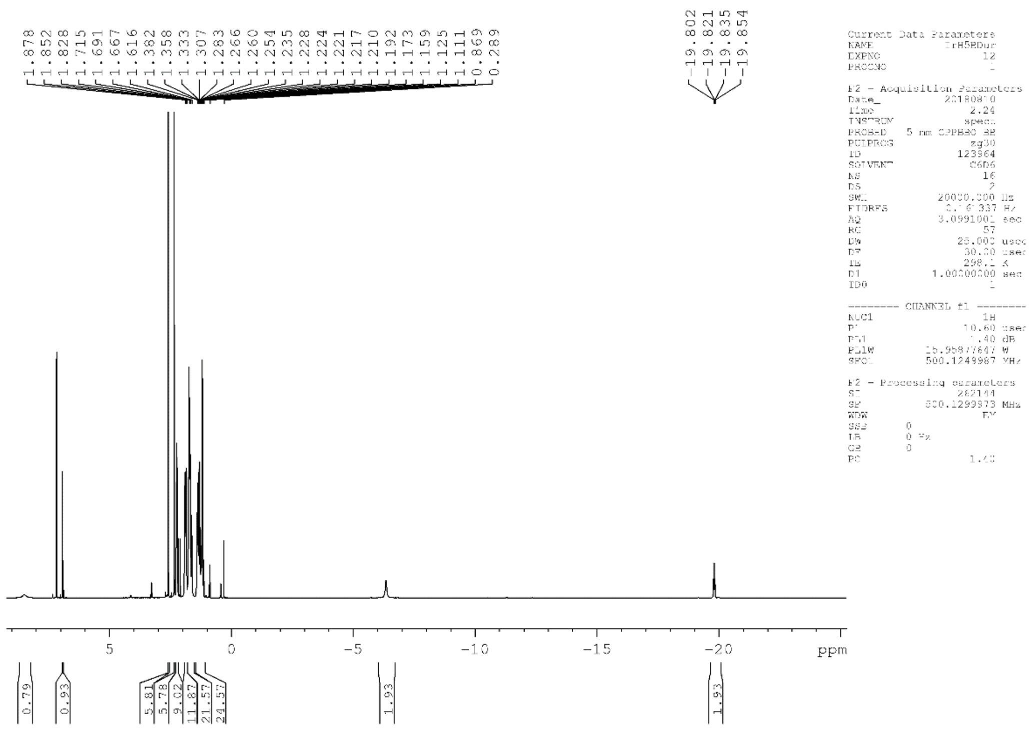

Figure S51: ^{1}H NMR spectrum of **[Ir(PCy$_3$)$_2$H$_2$(κ^2-H$_2$BDur)] (11)** in C_6D_6

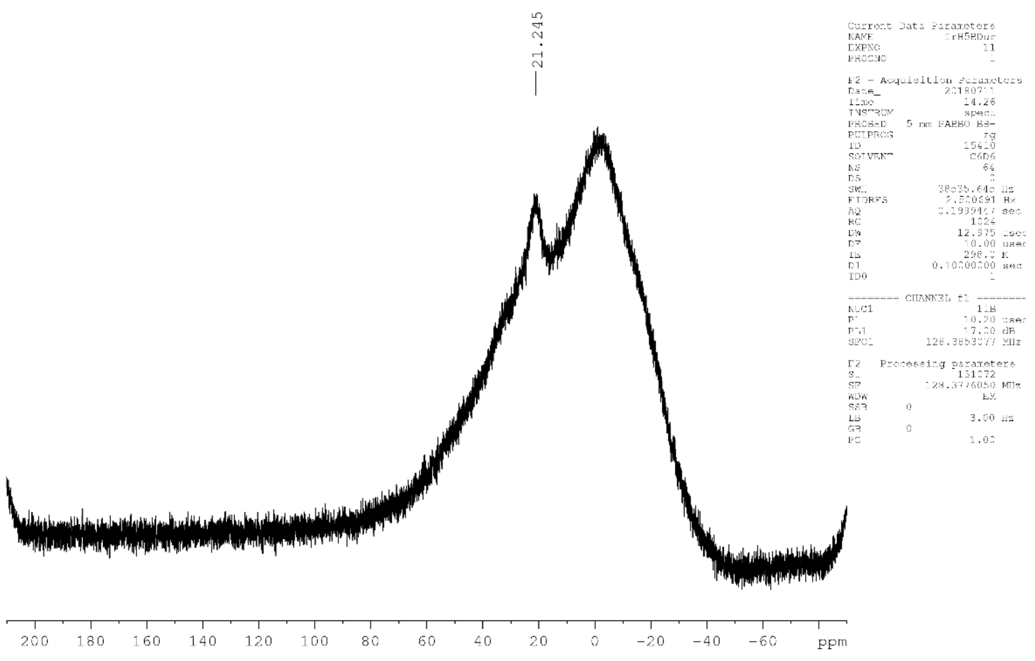

Figure S52: ^{11}B NMR spectrum of **[Ir(PCy$_3$)$_2$H$_2$(κ^2-H$_2$BDur)] (11)** in C_6D_6

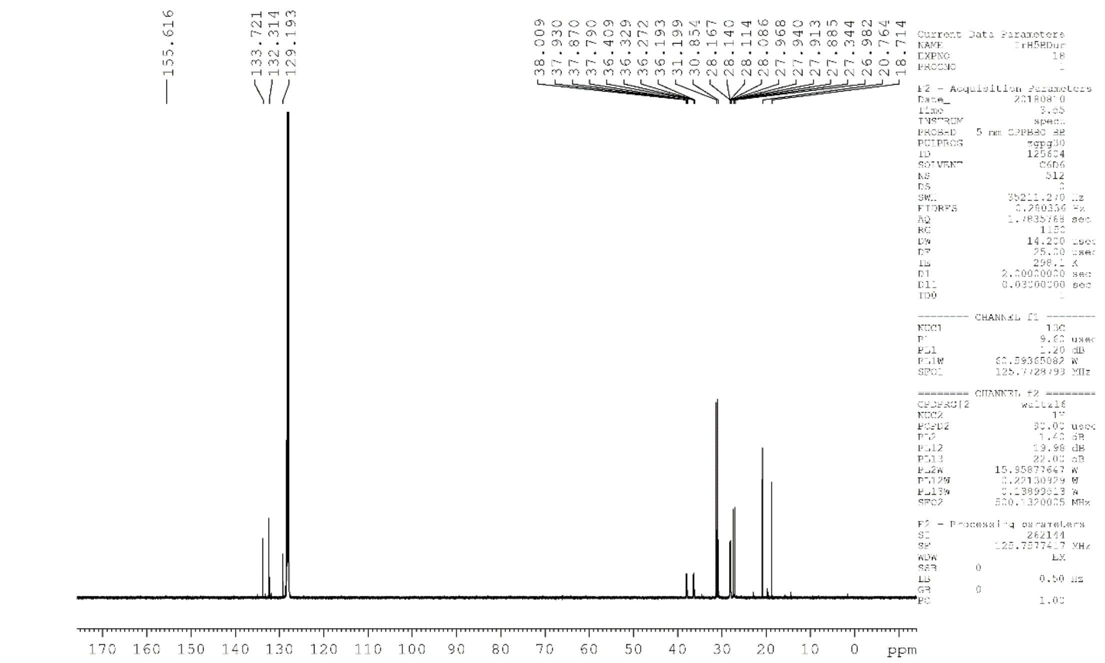

Figure S53: $^{13}C\{^{1}H\}$ NMR spectrum of **[Ir(PCy$_3$)$_2$H$_2$(κ^2-H$_2$BDur)] (11)** in C_6D_6

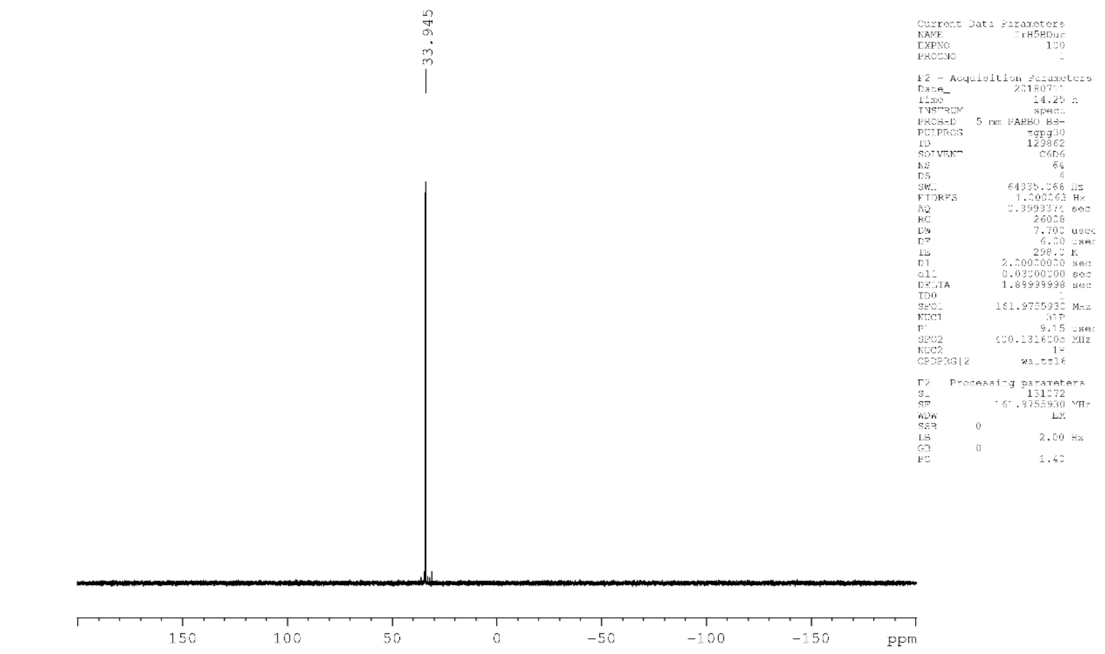

Figure S54: $^{31}P\{^{1}H\}$ NMR spectrum of **[Ir(PCy$_3$)$_2$H$_2$(κ^2-H$_2$BDur)] (11)** in C_6D_6

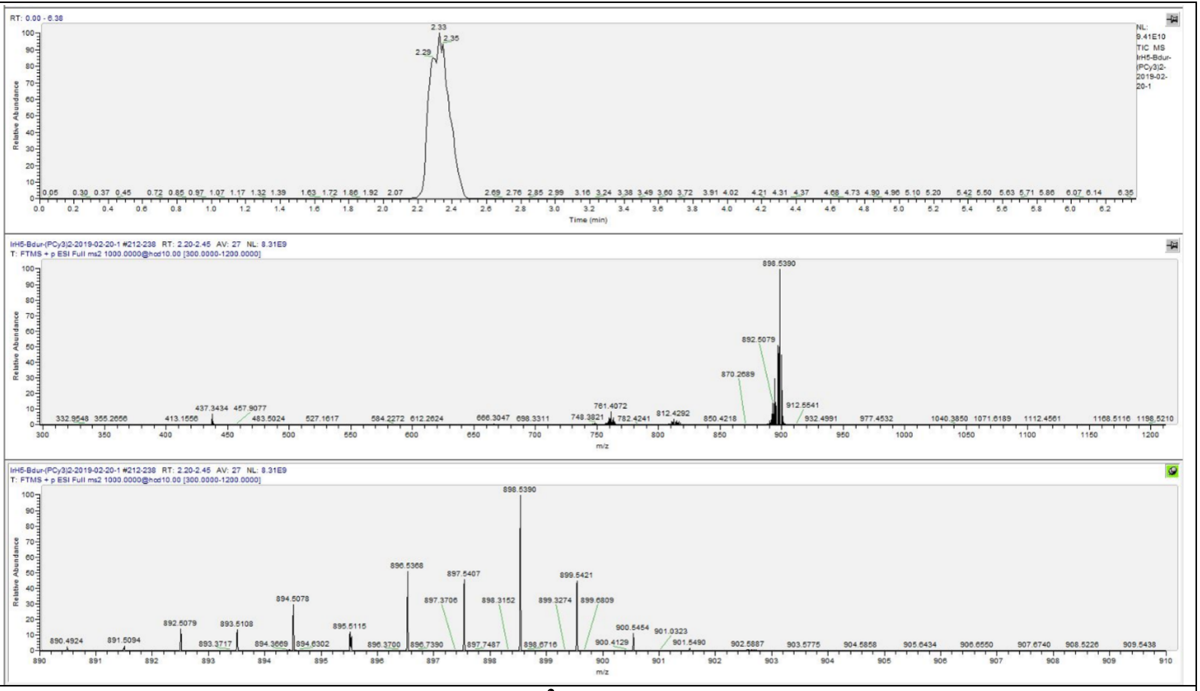

Figure S55: Mass spectrum of **[Ir(PCy$_3$)$_2$H$_2$(κ^2-H$_2$BDur)] (11)**

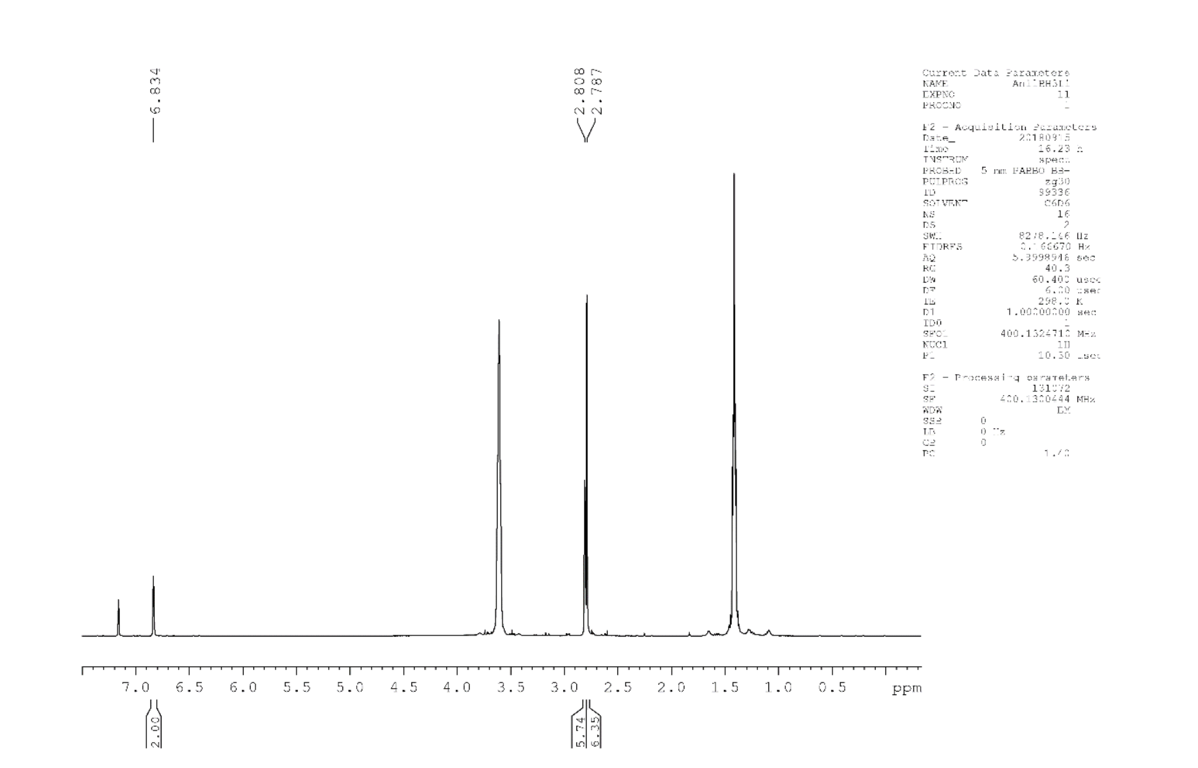

Figure S56: ^{1}H NMR spectrum of **Li[AnilBH$_3$]** in C_6D_6 / THF

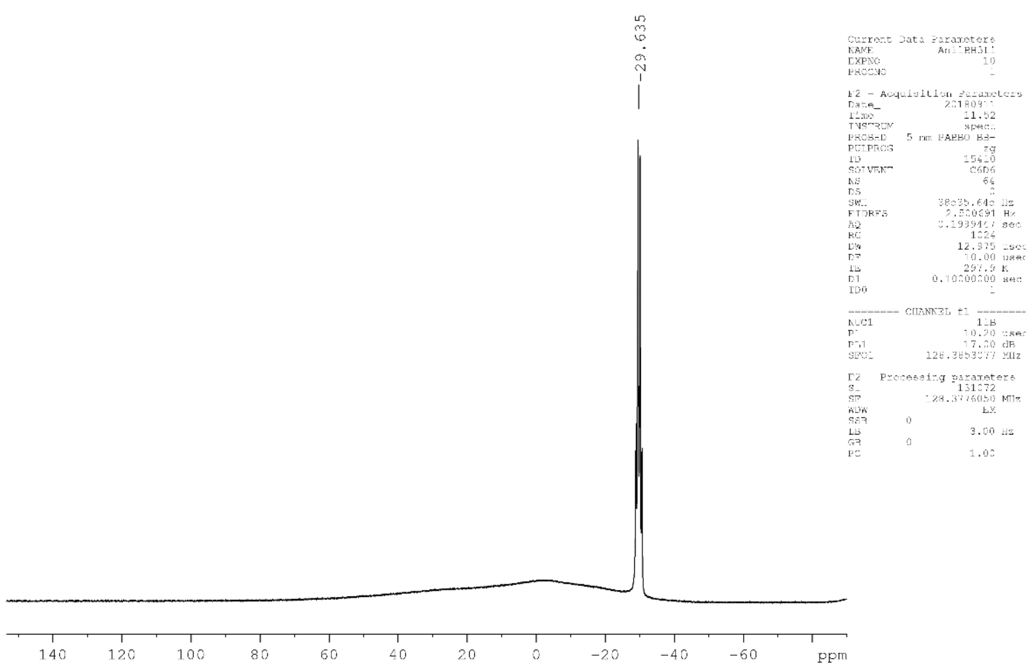

Figure S57: ^{11}B NMR spectrum of **Li[AnilBH$_3$]** in C_6D_6 / THF

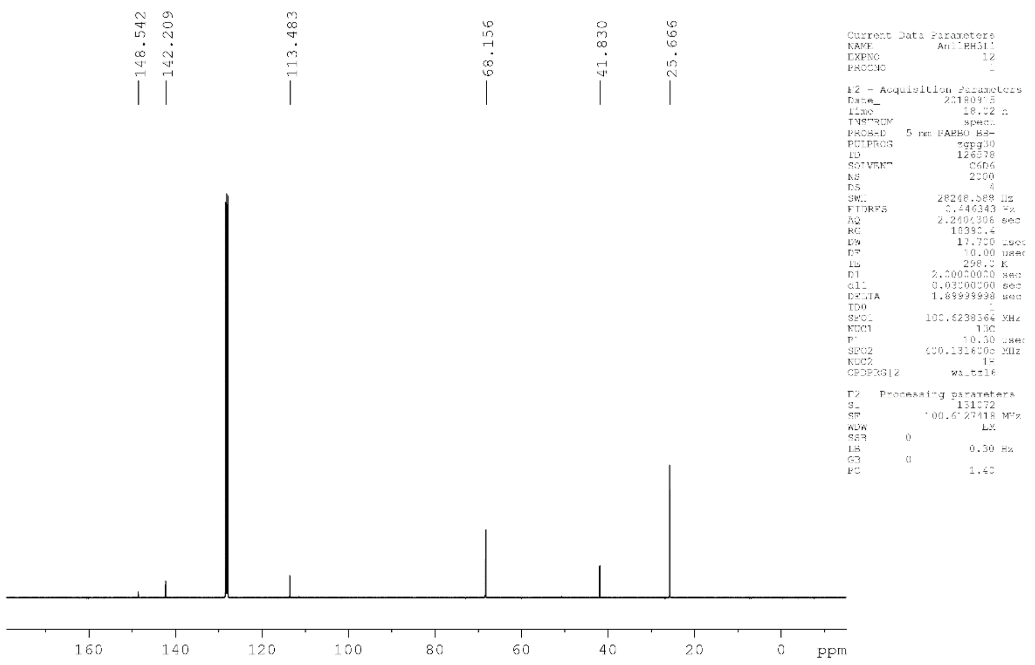

Figure S58: $^{13}C\{^1H\}$ NMR spectrum of **Li[AnilBH$_3$]** in C_6D_6 / THF

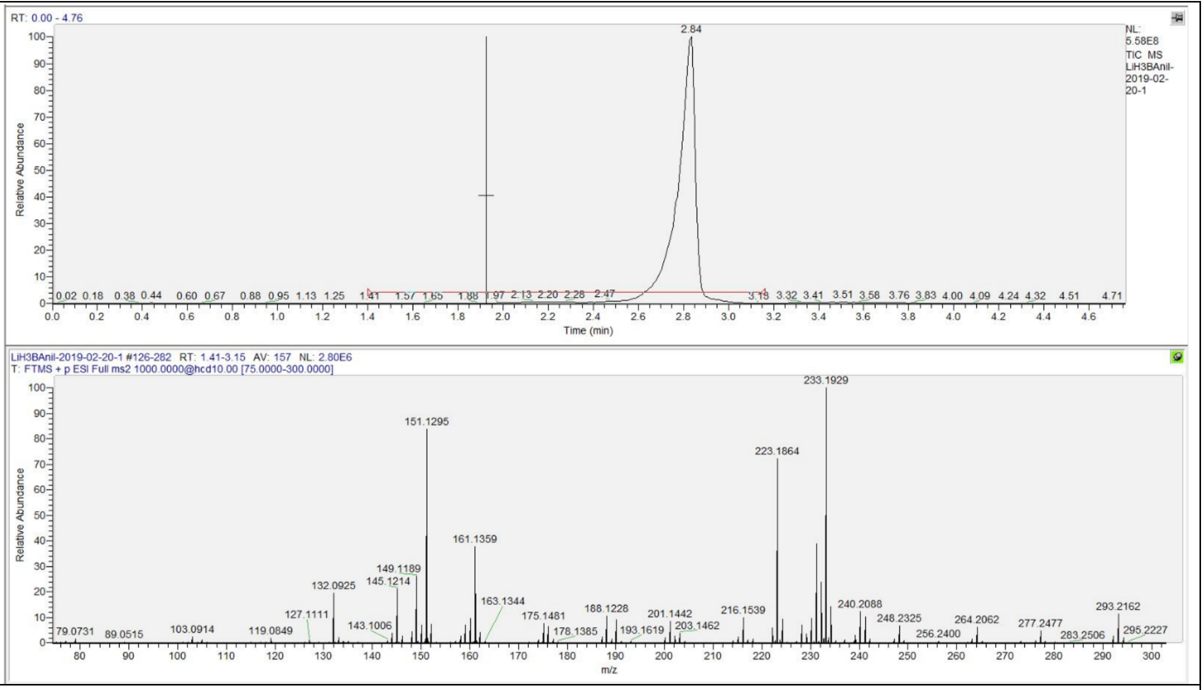

Figure S59: Mass spectrum of **Li[AnilBH$_3$]**

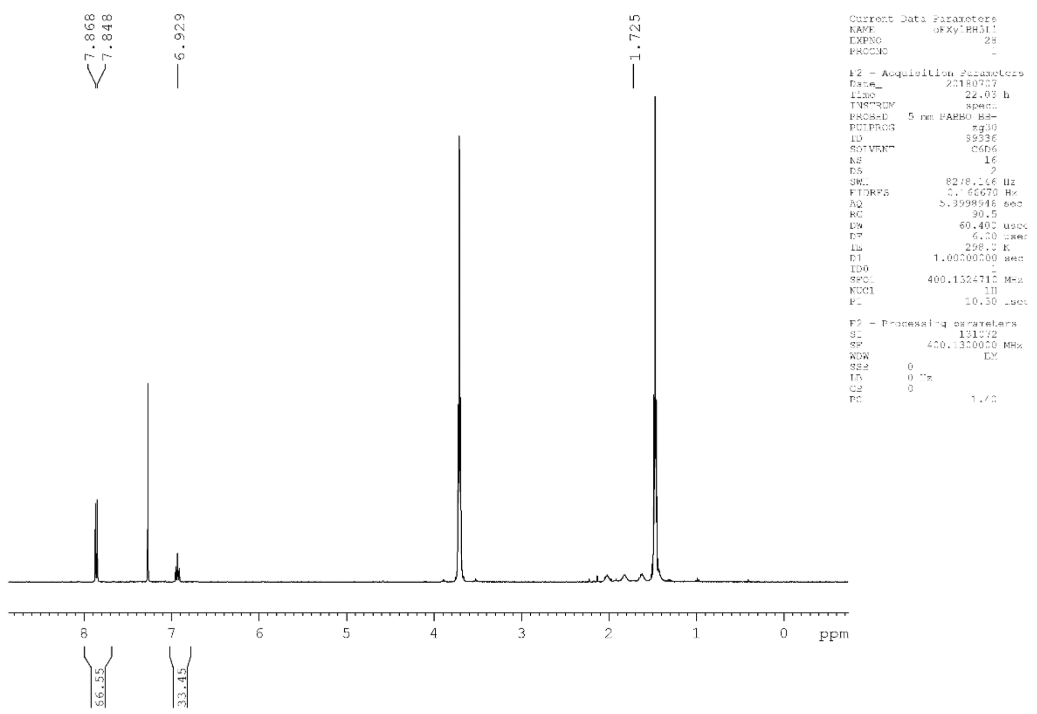

Figure S60: ^{1}H NMR spectrum of **Li[^{o}FXylBH$_3$]** in C_6D_6

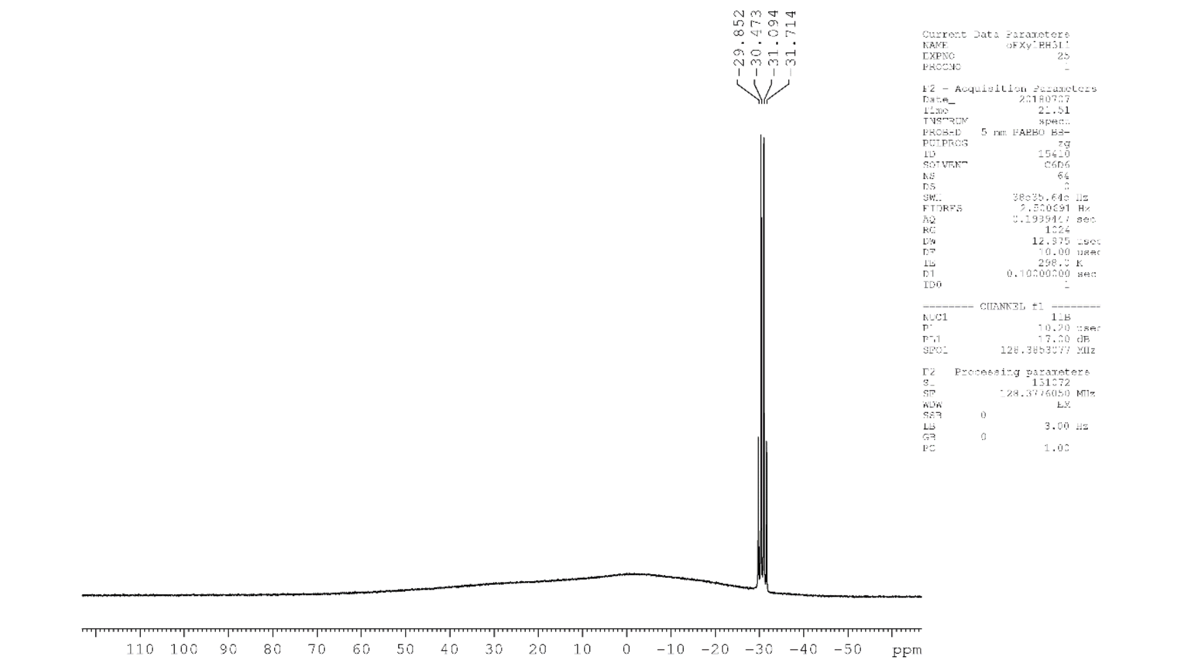

Figure S61: ^{11}B NMR spectrum of **Li[^{o}FXylBH$_3$]** in C_6D_6

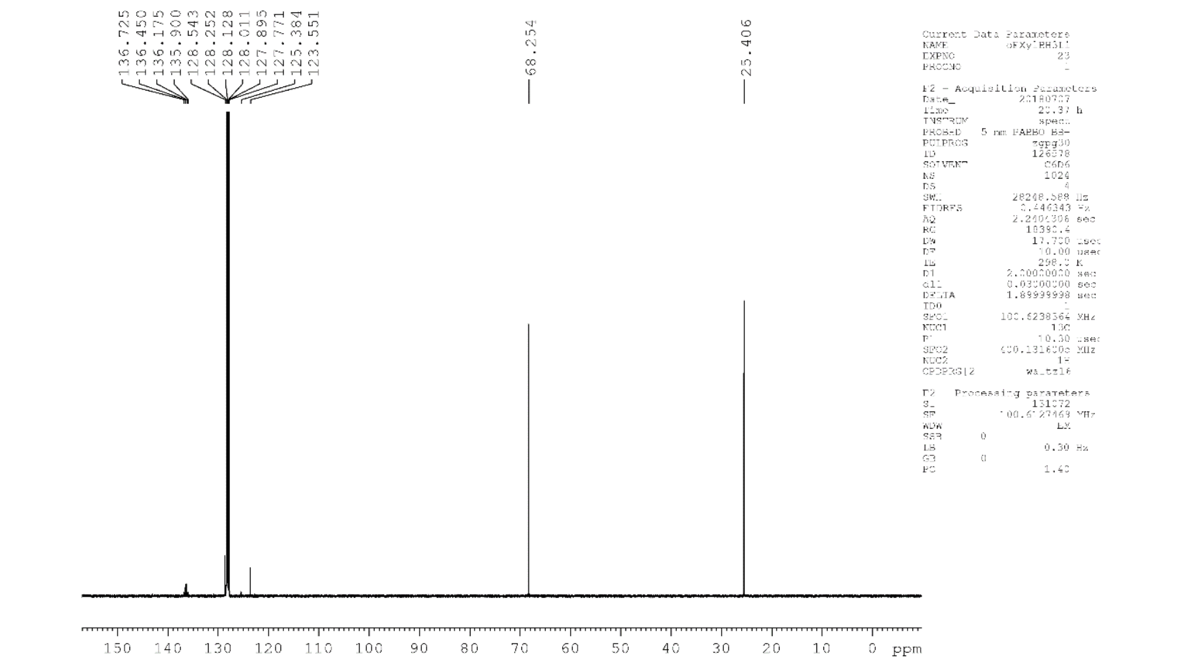

Figure S62: ^{13}C{^{1}H} NMR spectrum of **Li[^{o}FXylBH$_3$]** in C_6D_6

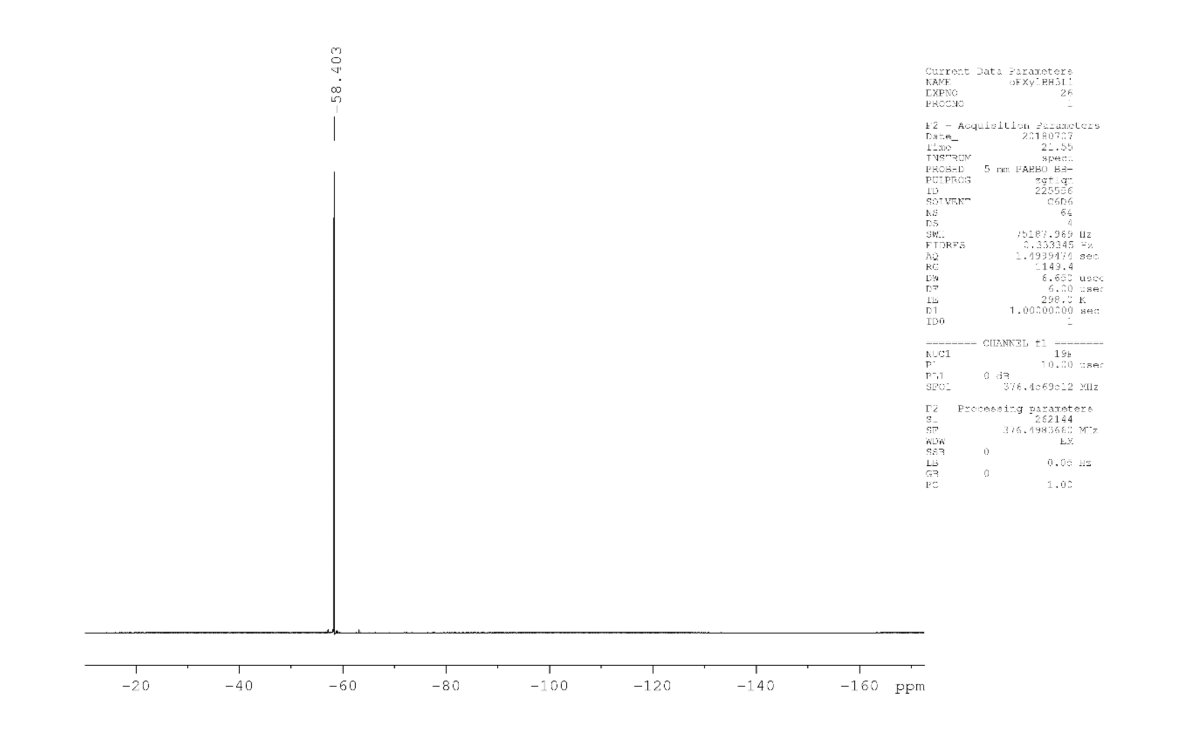

Figure S63: ^{19}F NMR spectrum of **Li[^{o}FXylBH$_3$]** in C_6D_6

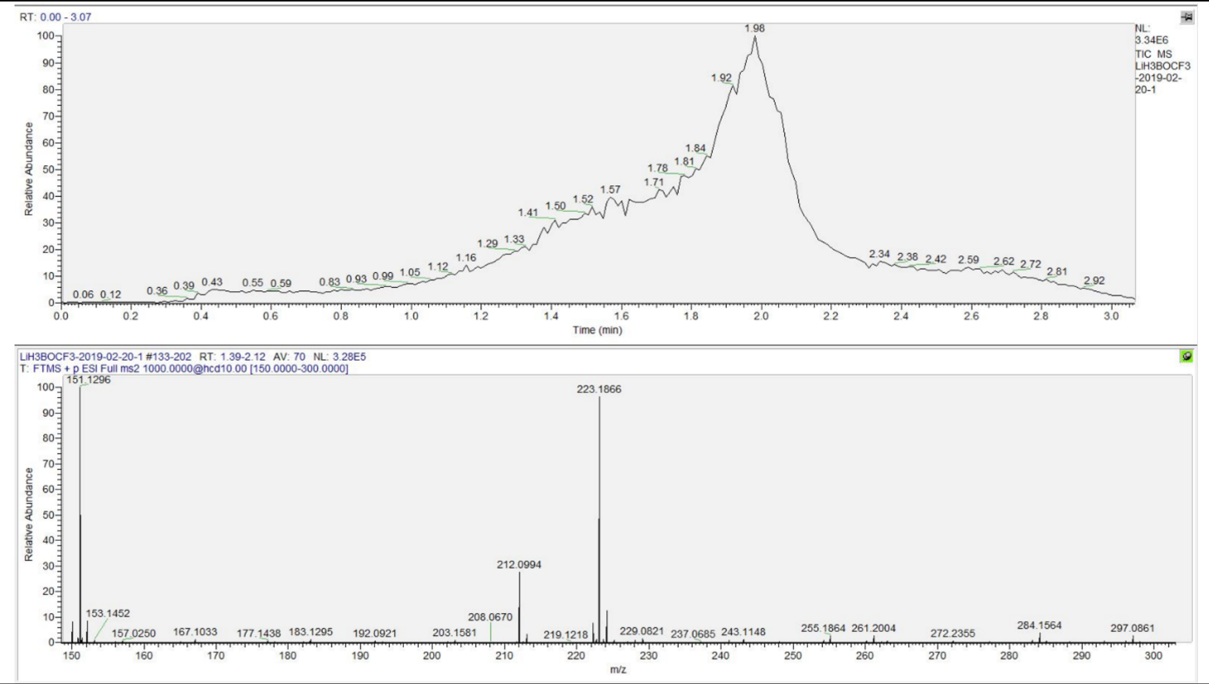

Figure S64: Mass spectrum of **Li[^{o}FXylBH$_3$]**

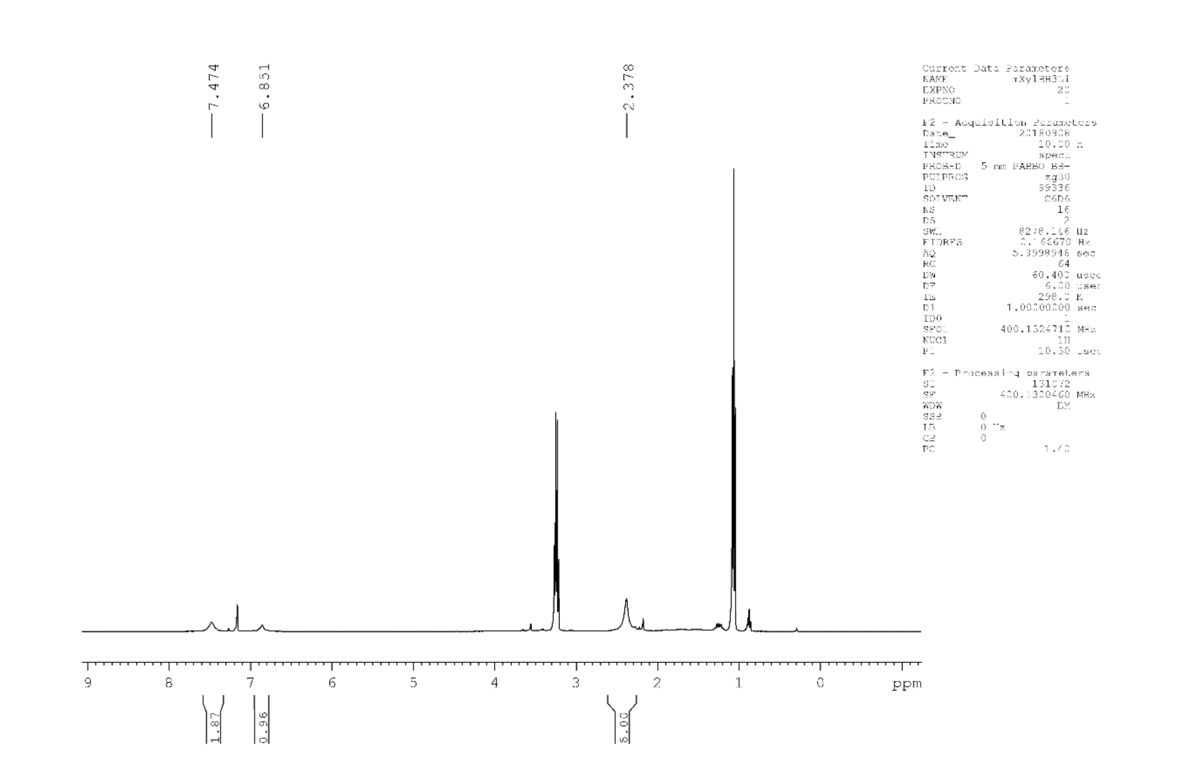

Figure S65: ^{1}H NMR spectrum of **Li[‴XylBH$_3$]** in C_6D_6 / Et_2O

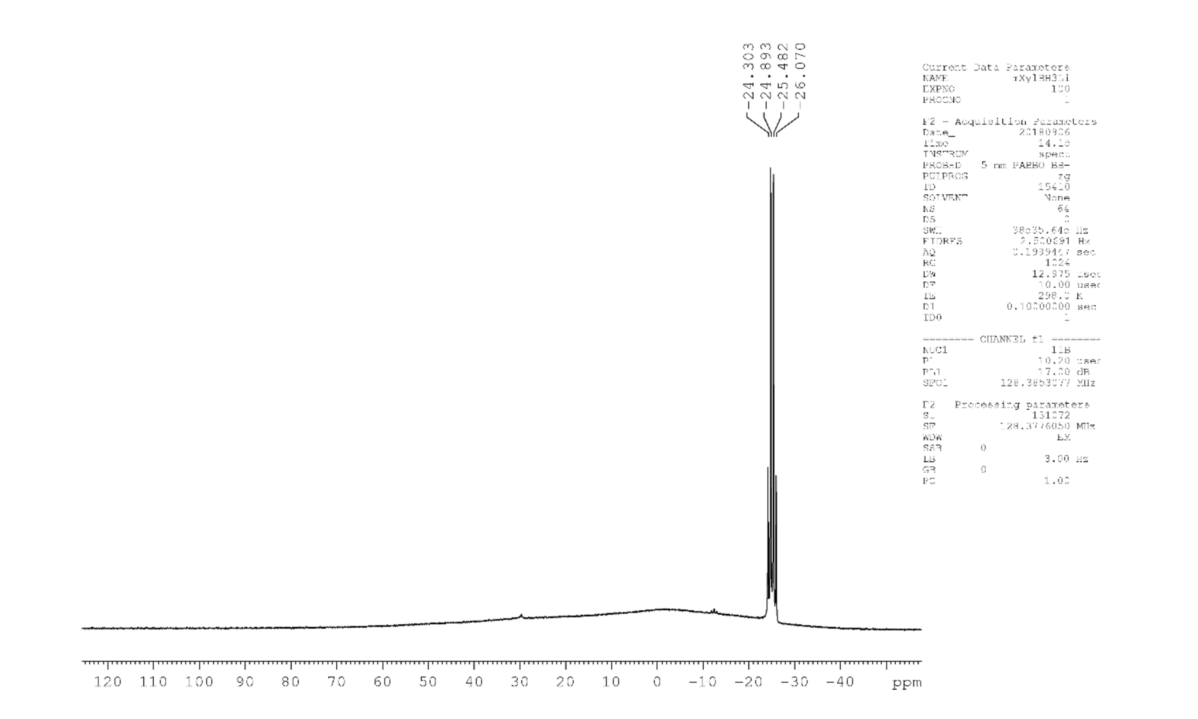

Figure S66: ^{11}B NMR spectrum of **Li[‴XylBH$_3$]** in C_6D_6 / Et_2O

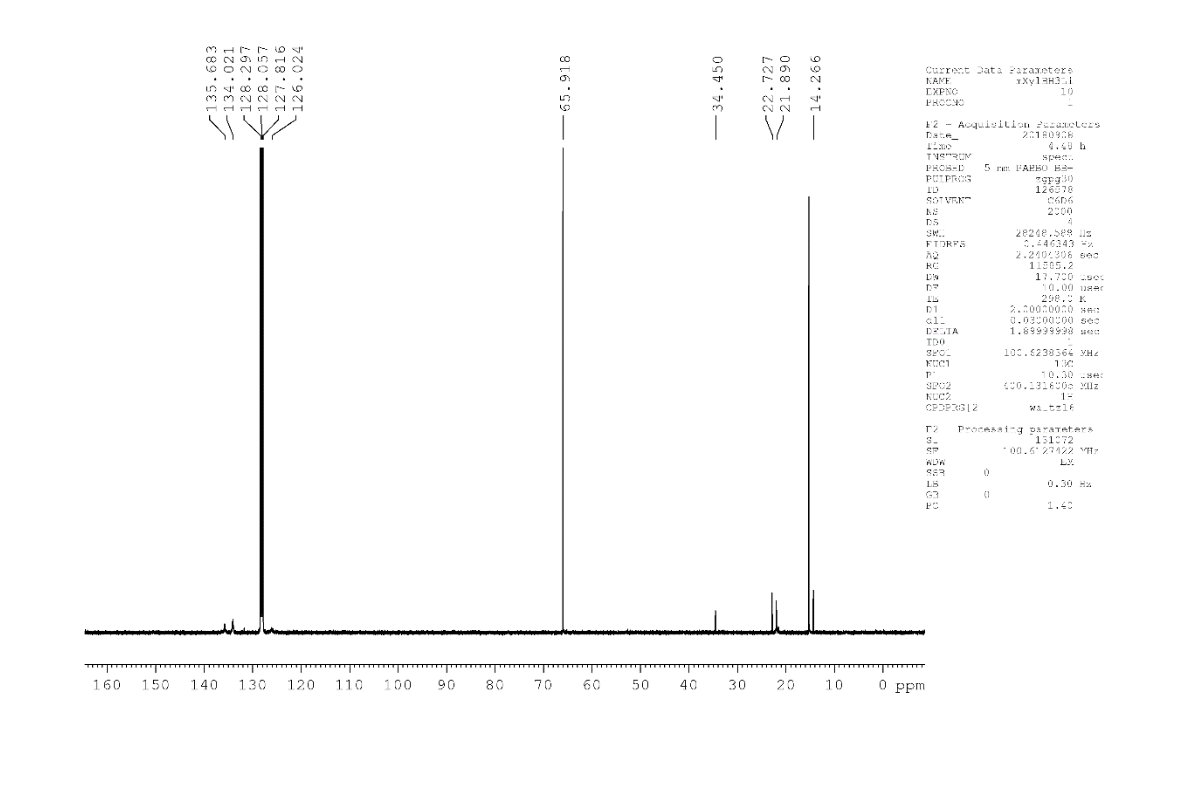

Figure S67: $^{13}C\{^{1}H\}$ NMR spectrum of **Li[‴XylBH$_3$]** in C_6D_6 / Et_2O

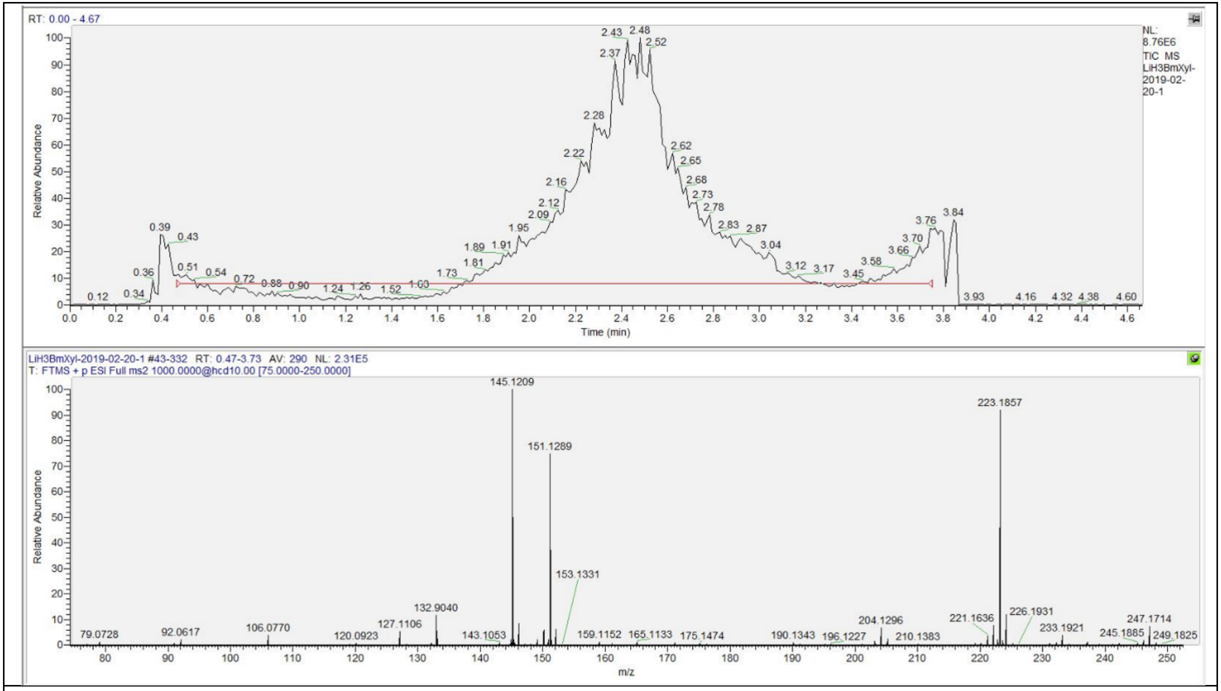

Figure S68: Mass spectrum of **Li[‴XylBH$_3$]**

Crystal structure determination

Crystallographic data have been deposited with the Cambridge Crystallographic Data Centre as supplementary publication no. CCDC 1922473-1922484. These data can be obtained free of charge from The Cambridge Crystallographic Data Centre *via* www.ccdc.cam.ac.uk/data_request/cif.

The crystal data of **[Ru(PCy$_3$)$_2$H$_2$-(σ^2-H$_2$B‴FXyl)] (5), [Ru(PCy$_3$)$_2$H$_2$-(σ^2-H$_2$B″Xyl)] (4), [Ru(PCy$_3$)$_2$HCl(BAnil)] (6), [Ru(PCy$_3$)$_2$H$_2$-(σ^2-H$_2$BAnil)] (1), [Ru(PCy$_3$)$_2$HCl(BDur)] (7), [Ru(PCy$_3$)$_2$H$_2$-(σ^2-H$_2$BDur)] (2), [Ru(PCy$_3$)$_2$H$_2$-(σ^2-H$_2$B^{o}FXyl)] (3), [Ru(PCy$_3$)$_2$HCl(B^{o}FXyl)] (8),** and **[(Cy$_3$P)$_2$Ir(H)$_2$(κ^2-H$_2$BHDur)] (11)** were collected on a BRUKER D8 QUEST diffractometer with a CMOS area detector and multi-layer mirror monochromated Mo$_{K\alpha}$ radiation. The structure was solved using intrinsic phasing method,[11] refined with the SHELXL program[12] and expanded using Fourier techniques. All non-hydrogen atoms were refined anisotropically. Hydrogen atoms were included in structure factor calculations. All hydrogen atoms except the terminal and bridging hydrogen atoms were assigned to idealized positions. The coordinates of the terminal and bridging hydrogen atoms were refined freely.

For **[Ru(PCy$_3$)$_2$H$_2$-(σ^2-H$_2$BAnil)] (1)** the displacement parameters of atoms in disordered cyclohexyl residue (3) were restrained to the same value with similarity restraint SIMU.

For **[Ru(PCy$_3$)$_2$H$_2$-(σ^2-H$_2$B‴FXyl)] (5)** the displacement parameters of two atoms (C1_7 C7_17) in disordered cyclohexyl residue 7 or 17 (part 1 and part 2) were constrained to the same value with the EADP keyword. The displacement parameters of atoms in the disordered cyclohexyl groups 4 and 7 (part 1) as well as 14 and 17 (part 2) were restrained to the same values with similarity restraint SIMU. The Uii displacement parameters were restrained with the ISOR keyword to approximate isotropic behavior.

For **[Ru(PCy$_3$)$_2$HCl(BAnil)] (6)** the displacement parameters of atoms in disordered cyclohexyl residues were restrained to the same value with similarity restraint SIMU. The atomic displacement parameters of those atoms were restrained with RIGU keyword in ShelXL input ('enhanced rigid bond' restraint for all bonds in the connectivity list. Standard values of 0.004 for both parameters s1 and s2 were used). The distances between the terminal hydrogen atom and Ru and this hydrogen atom and the boron atom were retrained to the values 1.158 (Ru-H) and 2.142 (B-H) during refinement using the DFIX restraint.

For **[Ru(PCy$_3$)$_2$HCl(BDur)] (7)** all hydrogen atoms except H22 were assigned to idealized positions. The coordinates of H22 were refined freely. The distances between atoms Ru1, H22 and H22 and B42 were retrained to the values 1.587 (Ru1-H22) and 2.142 (B42-H22) during refinement using the DFIX restraint.

Crystal data for **[Ru(PCy$_3$)$_2$H$_2$-(σ^2-H$_2$BAnil)] (1)**: $C_{46}H_{84}BNP_2Ru$, M_r = 824.96, colorless plate, 0.455×0.204×0.125 mm^3, triclinic space group $P\bar{1}$, a = 9.6335(15) Å, b = 12.607(2) Å, c = 19.960(4) Å, α = 79.589(12)°, β = 76.551(9)°, γ = 72.347(8)°, V = 2230.7(7) Å^3, Z = 2, ρ_{calcd} = 1.228 g·cm^{-3}, μ = 0.454 mm^{-1}, F(000) = 892, T = 100(2) K, R_1 = 0.0348, wR^2 = 0.0685, 8782 independent reflections [2θ≤52.042°] and 531 parameters. CCDC 1922473.

Crystal data for **[Ru(PCy$_3$)$_2$H$_2$-(σ^2-H$_2$BDur)] (2)**: $C_{46}H_{83}BP_2Ru$, M_r = 809.94, yellow block, 0.34×0.23×0.19 mm^3, monoclinic space group Pn, a = 9.560(3) Å, b = 13.615(4) Å, c = 16.967(4) Å, β = 90.551(9)°, V = 2208.4(10) Å^3, Z = 2, ρ_{calcd} = 1.218 g·cm^{-3}, μ = 0.457 mm^{-1}, F(000) = 876, T = 100(2) K, R_1 = 0.0228, wR^2 = 0.0469, 7914 independent reflections [2θ≤52.044°] and 467 parameters. CCDC 1922474.

Crystal data for **[Ru(PCy$_3$)$_2$H$_2$-(σ^2-H$_2$B^{o}FXyl)] (3)**: $C_{44}H_{73}BF_6P_2Ru$, M_r = 889.84, orange block, 0.281×0.189×0.149 mm^3, triclinic space group $P\bar{1}$, a = 10.4022(19) Å, b = 12.879(2) Å, c = 18.200(4) Å, α = 96.503(11)°, β = 96.075(15)°, γ = 113.545(7)°, V = 2190.1(7) Å^3, Z = 2, ρ_{calcd} = 1.349 g·cm^{-3}, μ = 0.486 mm^{-1}, F(000) = 940, T = 100(2) K, R_1 = 0.0258, wR^2 = 0.0528, 8592 independent reflections [2θ≤52.04°] and 499 parameters. CCDC 1922475.

Crystal data for **[Ru(PCy$_3$)$_2$H$_2$-(σ^2-H$_2$B$'''$Xyl)] (4)**: $C_{44}H_{79}BP_2Ru$, M_r = 781.89, colorless plate, 0.168×0.151×0.107 mm^3, triclinic space group $P\bar{1}$, a = 12.453(3) Å, b = 13.336(2) Å, c = 14.969(2) Å, α = 66.157(6)°, β = 72.914(4)°, γ = 67.116(7)°, V = 2067.1(7) Å^3, Z = 2, ρ_{calcd} = 1.256 g·cm^{-3}, μ = 0.486 mm^{-1}, F(000) = 844, T = 100(2) K, R_1 = 0.0400, wR^2 = 0.0751, 8144 independent reflections [2θ≤52.042°] and 447 parameters. CCDC 1922476.

Crystal data for **[Ru(PCy$_3$)$_2$H$_2$-(σ^2-H$_2$B$'''$FXyl)] (5)**: $C_{44}H_{73}BF_6P_2Ru$, M_r = 889.84, yellow plate, 0.17×0.14×0.10 mm^3, monoclinic space group $C2/c$, a = 43.702(11) Å, b = 11.707(4) Å, c = 17.081(5) Å, β = 93.093(15)°, V = 8727(5) Å^3, Z = 8, ρ_{calcd} = 1.355 g·cm^{-3}, μ = 0.488 mm^{-1}, F(000) = 3760, T = 100(2) K, R_1 = 0.0393, wR^2 = 0.0876, 8602 independent reflections [2θ≤52.036°] and 603 parameters. CCDC 1922477.

Crystal data for **[Ru(PCy$_3$)$_2$HCl(BAnil)] (6)**: $C_{92}H_{162}B_2Cl_2N_2P_4Ru_2$, M_r = 1714.77, yellow plate, 0.22×0.10×0.07 mm^3, monoclinic space group $P2_1/c$, a = 21.139(4) Å, b = 21.509(5) Å, c = 19.849(5) Å, β = 93.006(13)°, V = 9013(4) Å^3, Z = 4, ρ_{calcd} = 1.264 g·cm^{-3}, μ = 0.510 mm^{-1}, F(000) = 3680, T = 100(2) K, R_1 = 0.0538, wR^2 = 0.1239, 17773 independent reflections [2θ≤52.044°] and 1063 parameters. CCDC 1922478.

Crystal data for **[Ru(PCy$_3$)$_2$HCl(BDur)] (7)**: $C_{46}H_{80}BClP_2Ru$, M_r = 842.37, yellow plate, 0.10×0.10×0.05 mm^3, monoclinic space group $P2_1/c$, a = 15.321(4) Å, b = 14.947(3) Å, c = 20.1812(19) Å, β = 99.326(7)°, V = 4560.6(15) Å^3, Z = 4, ρ_{calcd} = 1.227 g·cm^{-3}, μ = 0.502 mm^{-1}, F(000) = 1808, T = 100(2) K, R_1 = 0.0273, wR^2 = 0.0550, 8984 independent reflections [2θ≤52.044°] and 468 parameters. CCDC 1922479.

Crystal data for **[Ru(PCy$_3$)$_2$HCl(B^{o}FXyl)] (8)**: $C_{50}H_{76}BClF_6P_2Ru$, M_r = 1000.37, orange block, 0.339×0.282×0.18 mm^3, monoclinic space group $P2_1/n$, a = 17.679(4) Å, b = 15.004(5) Å, c = 19.256(5) Å, β = 107.793(17)°, V = 4864(2) Å^3, Z = 4, ρ_{calcd} = 1.366 g·cm^{-3}, μ = 0.500 mm^{-1}, F(000) = 2104, T = 100(2) K, R_1 = 0.0283, wR^2 = 0.0723, 9582 independent reflections [2θ≤52.038°] and 553 parameters. CCDC 1922480.

Crystal data for **[(Cy$_3$P)$_2$Ru(H)$_2$(κ^2-H$_2$B(^{o}FXyl)$_2$)] (10):** $C_{52}H_{76}BF_{12}P_2Ru$, M_r = 1102.94, orange block, 0.362×0.333×0.316 mm^3, monoclinic space group $C2/c$, a = 22.640(11) Å, b = 15.981(6) Å, c = 16.143(8) Å, β = 116.000(14)°, V = 5250(4) Å^3, Z = 8, ρ_{calcd} = 1.396 g·cm^{-3}, μ = 0.437 mm^{-1}, F(000) = 2300, T = 100(2) K, R_1 = 0.0437, wR^2 = 0.1052, 5166 independent reflections [2θ≤52.044°] and 396 parameters. CCDC 1922481.

Crystal data for **[(Cy$_3$P)$_2$Ir(H)$_2$(κ^2-H$_2$BHDur)] (11):** $C_{46}H_{83}BIrP_2$, M_r = 901.07, colorless block, 0.809×0.186×0.122 mm^3, monoclinic space group P21/n $C2/c$, a = 9.8860(14) Å, b = 28.106(5) Å, c = 16.3445(19) Å, β = 100.796(9)°, V = 4461.1(11) Å^3, Z = 4, ρ_{calcd} = 1.342 g·cm^{-3}, μ = 3.094 mm^{-1}, F(000) = 1884, T = 100(2) K, R_1 = 0.0227, wR^2 = 0.0520, 8774 independent reflections [2θ≤52.04°] and 470 parameters. CCDC 1922482

Computational Details

All calculations were carried out using the Amsterdam Density Functional (ADF) program.[13] The numerical integration was performed by using a procedure developed by Becke *et al.*[14] The molecular orbitals (MOs) were expanded in a large uncontracted set of Slater-type orbitals (STOs, no Gaussian functions are involved) containing diffuse functions: A triple-ζ quality basis set was used for all atoms,[15] augmented with two sets of polarization functions for H (2p, 3d), B, C, N, O, F, P, Si, Cl (3d, 4f) and Ru (5p, 4f). An auxiliary set of s, p, d, f and g STOs was used to fit the molecular density and to represent the Coulomb and exchange potentials accurately in each self-consistent field (SCF) cycle. All electrons were included in the variational treatment (no frozen-core approximation was used). The generalized gradient approximation (GGA) at the BLYP level was used; exchange is described by Slater's Xα potential,[16] with nonlocal corrections due to Becke[17] added self-consistently, and correlation was treated by using the Lee-Yang-Parr gradient-corrected functional.[18] Relativistic effects were included with the scalar-zero-order-regular-approximation (ZORA).[19] In addition, the D3(BJ) dispersion correction was used.[20] Energy minima have been verified through vibrational analysis.[21] For the thermochemistry calculations of the stepwise coordination reactions we used a standard approach as described by Swart and Bickelhaupt in Ref.[22] Geometries were optimized and the vibrational frequencies were obtained through numerical differentiation of the analytical gradient.[13c] Enthalpies at 298.15 K and 1 atmosphere (ΔH) were calculated from electronic bond energies and vibrational frequencies using a standard thermochemistry relation for an ideal gas (Eq. 1).[23]

$$\Delta H = \Delta E_{\mathrm{trans},298} + \Delta E_{\mathrm{rot},298} + \Delta E_{\mathrm{vib},0} + \Delta(\Delta E_{\mathrm{vib},298}) + \Delta(pV) \quad (1)$$

$\Delta E_{\mathrm{trans},298}$, $\Delta E_{\mathrm{rot},298}$ and $\Delta E_{\mathrm{vib},0}$ are the differences between the reactants (TM-H_2) and the products (TM + H_2) in translational, rotational and zero-point vibrational energy, respectively, while Δ ($\Delta E_{\mathrm{vib},298}$) takes the vibrational energy change upon going from 0 to 298.15 K into account. The vibrational energy corrections and entropic term $T\Delta S°$ are based on our frequency calculations. Thermal corrections for the electronic term are neglected and $\Delta(pV) \approx \Delta(\mathrm{nRT})$. The change of Gibbs free energy (ΔG) in the gas phase was then calculated for 298.15 K and 1 atm (ΔG) (Eq. 2).

$$\Delta G = \Delta H - T\Delta S \quad (2)$$

XYZ Coordinates

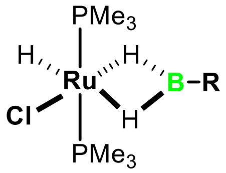

TM-H_2

**R** =	
m-Xylyl	**A**
m-FXylyl	**B**
2,6-dimethyl-4-*N*,*N*-dimethylaniline	**C**
Duryl	**D**
o-FXylyl	**E**

**A**		**NOIMAG**	**[-5925.29]**	**B**		**NOIMAG**	**[-5975.88]**	**C**		**NOIMAG**	**[-6913.31]**
Ru	5.469831	7.614309	4.775632	Ru	5.521895	7.617783	4.784968	Ru	5.436925	7.639168	4.755971
H	5.490394	7.387535	3.187906	H	5.675187	7.346364	3.21884	H	5.367253	7.323779	3.175321
P	5.266924	9.904498	4.264784	P	5.313124	9.893218	4.186485	P	5.081363	9.878964	4.137832
H	5.811376	7.881753	6.732454	H	5.77312	7.957079	6.798201	H	5.823628	8.030558	6.601587
H	7.031623	7.613962	4.369024	H	7.098094	7.625688	4.455681	H	7.012647	7.730849	4.38863
P	5.350142	5.272703	4.857963	P	5.416896	5.270878	4.922389	P	5.446165	5.308612	4.962707
Cl	2.97715	7.589478	4.880953	Cl	3.040195	7.560602	4.746235	Cl	2.947478	7.478646	4.928181
B	6.872522	7.673129	6.076334	B	6.834944	7.745652	6.16668	B	6.91636	7.807403	5.977775
C	4.29579	4.527433	3.546103	C	4.463184	4.490371	3.556728	C	4.401271	4.430785	3.727114
H	3.312436	5.003647	3.594554	H	3.474682	4.956923	3.525542	H	3.39562	4.857924	3.776719
C	4.620477	4.589543	6.407243	C	4.58603	4.62431	6.434492	C	4.798916	4.667351	6.567377
C	6.932831	4.337046	4.697564	C	7.014726	4.349478	4.89433	C	7.073104	4.446909	4.810499
H	7.608508	4.631856	5.506418	H	7.623393	4.653431	5.751512	H	7.754556	4.832912	5.575307
C	6.823763	10.832818	3.915922	C	6.874617	10.831771	3.896889	C	6.571762	10.878786	3.69699
H	7.47595	10.778073	4.793436	H	7.47216	10.825058	4.814061	H	7.249014	10.905985	4.556825
C	4.232493	10.253069	2.782896	C	4.361106	10.170371	2.637339	C	3.989695	10.099655	2.67227
H	3.264599	9.76476	2.927926	H	3.392511	9.673881	2.743757	H	3.057146	9.562571	2.868034
C	4.461568	10.921039	5.575554	C	4.418559	10.9312	5.418447	C	4.256818	10.920331	5.418409
H	4.320731	11.959514	5.253059	H	4.280242	11.95635	5.055403	H	4.045681	11.930565	5.047712
H	7.338503	10.356333	3.07576	H	7.447854	10.333256	3.109116	H	7.089752	10.392015	2.864633
H	6.621812	11.883004	3.673936	H	6.671379	11.867743	3.601536	H	6.303187	11.902762	3.411046
H	4.097184	11.331076	2.636848	H	4.221001	11.240532	2.446308	H	3.786432	11.159958	2.481318
H	4.715097	9.817472	1.902796	H	4.899408	9.7129	1.801795	H	4.473827	9.650708	1.799812
H	3.493961	10.464335	5.804493	H	3.446548	10.465794	5.607034	H	3.32505	10.421701	5.702094

H	5.082323	10.896886	6.477025	H	4.987338	10.94696	6.353725	H	4.904182	10.979553	6.299398
H	6.764713	3.254305	4.734204	H	6.851982	3.266123	4.929813	H	6.967344	3.361668	4.925073
H	7.403432	4.600964	3.745194	H	7.557388	4.605659	3.979098	H	7.498065	4.667603	3.82613
H	5.265745	4.854092	7.251114	H	5.168452	4.914491	7.314829	H	5.460997	4.996917	7.374503
H	3.643009	5.059429	6.552353	H	3.597264	5.088174	6.499542	H	3.80796	5.103384	6.725789
H	4.509081	3.50001	6.355992	H	4.486838	3.533061	6.403065	H	4.732788	3.572853	6.571324
H	4.740132	4.742336	2.569558	H	4.973408	4.68673	2.608963	H	4.808132	4.613149	2.727932
H	4.202209	3.443525	3.680862	H	4.369573	3.409106	3.709815	H	4.369442	3.352969	3.925412
C	8.271292	7.53804	6.68848	C	8.229204	7.65706	6.821271	C	8.278757	7.732008	6.657166
C	8.455032	7.613184	8.085514	C	8.391391	7.838481	8.209544	C	8.35617	7.669844	8.084066
C	9.406097	7.30653	5.87996	C	9.377589	7.364224	6.058666	C	9.509253	7.68045	5.932317
C	9.719581	7.460899	8.66983	C	9.650816	7.731744	8.808285	C	9.583993	7.562876	8.731363
C	10.681605	7.155203	6.431342	H	7.52305	8.06404	8.823795	C	10.727515	7.578468	6.599273
C	10.818267	7.235293	7.827966	C	10.63343	7.253629	6.659642	C	10.803705	7.525902	8.012559
H	11.808482	7.120092	8.27002	H	9.277928	7.220545	4.985709	H	9.068291	8.64817	4.037833
C	11.894781	6.920295	5.555666	C	10.779806	7.436181	8.039011	H	11.634768	7.540613	6.006317
H	12.442003	6.021635	5.867692	H	11.754901	7.351539	8.504112	H	9.590291	7.50687	9.814351
H	11.608524	6.797191	4.506037	C	9.766001	7.932873	10.303077	H	7.363584	7.630376	10.005761
H	12.595434	7.763604	5.617587	C	11.827103	6.911008	5.796393	N	12.022179	7.449346	8.665482
C	9.895655	7.509615	10.173386	F	11.720794	5.643808	5.272165	C	12.063406	7.233118	10.110976
H	9.09495	8.085999	10.64928	F	13.008273	6.961484	6.480431	C	13.250065	7.243936	7.89877
H	9.873924	6.49816	10.602546	F	11.942033	7.763793	4.726896	H	13.103878	7.241339	10.44143
H	10.855781	7.960663	10.449143	F	11.053098	7.844862	10.752056	H	11.533814	8.033241	10.642602
H	7.591407	7.792894	8.724489	F	9.03341	6.998848	10.995249	H	11.612077	6.270784	10.402273
H	9.278968	7.24541	4.800208	F	9.288053	9.162136	10.685842	H	14.100359	7.246089	8.583472
								H	13.245214	6.287631	7.350993
								H	13.399318	8.052413	7.172434
								C	9.542741	7.735859	4.416821
								H	10.574718	7.71027	4.051788
								H	9.001205	6.893536	3.971766
								C	7.105705	7.703457	8.944197
								H	6.539474	8.629693	8.7922
								H	6.425371	6.879479	8.700258

**D**		**NOIMAG**	**[-6658.33]**	**E**		**NOIMAG**	**[-5969.32]**
Ru	5.477798	7.383739	4.80208	Ru	5.462185	7.583658	4.882628
H	5.383488	7.545565	3.201973	H	5.460721	6.779711	3.503791
P	5.547751	9.727312	4.732982	P	4.879527	9.449324	3.543731

H	5.846765	7.143469	6.683479	H	5.961631	8.649935	6.492911
H	6.978812	7.271558	4.255972	H	7.065737	7.74749	4.552749
P	4.984991	5.10631	4.407966	P	5.570898	5.398748	5.749515
Cl	2.99499	7.655218	5.043191	Cl	3.012428	7.278331	5.071467
B	6.934756	7.300706	6.041085	B	6.974775	8.164081	5.941106
C	3.819278	4.842433	3.007649	C	4.806191	4.158553	4.623489
H	2.938106	5.465693	3.182822	H	3.786471	4.487349	4.40464
C	4.139725	4.262333	5.81329	C	4.678603	5.102228	7.333658
C	6.356321	3.934646	4.007828	C	7.235982	4.672497	6.078677
H	7.053113	3.887195	4.850065	H	7.732617	5.25214	6.862861
C	7.207648	10.524038	4.605447	C	6.253411	10.418062	2.784659
H	7.809716	10.244331	5.475434	H	6.884962	10.83185	3.576477
C	4.63648	10.433343	3.297308	C	3.82786	9.025724	2.095267
H	3.616747	10.038379	3.323219	H	2.955409	8.4767	2.459457
C	4.77281	10.606749	6.155914	C	3.895533	10.739754	4.41764
H	4.740687	11.688656	5.981581	H	3.587822	11.540006	3.734222
H	7.710612	10.148173	3.708917	H	6.866426	9.752029	2.170855
H	7.125494	11.615701	4.548584	H	5.86315	11.233724	2.164735
H	4.619914	11.528828	3.336745	H	3.513775	9.929617	1.560592
H	5.117154	10.101731	2.372277	H	4.396563	8.376453	1.422857
H	3.758742	10.214784	6.277028	H	3.015543	10.255972	4.851089
H	5.341272	10.400773	7.066304	H	4.499296	11.160716	5.228058
H	5.967058	2.930204	3.803645	H	7.15401	3.626904	6.397393
H	6.898197	4.298783	3.129507	H	7.841427	4.732991	5.17007
H	4.816797	4.237594	6.673333	H	5.157182	5.672147	8.133228
H	3.258745	4.854848	6.076969	H	3.651389	5.457098	7.213378
H	3.841904	3.240455	5.550074	H	4.681697	4.035696	7.587574
H	4.30029	5.167883	2.080272	H	5.373949	4.126267	3.689007
H	3.531263	3.787809	2.926366	H	4.79255	3.164198	5.084849
C	8.338051	7.473622	6.653845	C	8.3886	8.243665	6.615999
C	8.475446	8.258854	7.837246	C	8.504837	8.43544	8.024762
C	9.500183	6.931774	6.033278	C	9.620687	8.089137	5.916093
C	9.755663	8.521108	8.364971	C	9.741797	8.434369	8.677672
C	10.774694	7.192961	6.574345	C	10.8551	8.0912	6.574526
C	10.875401	7.98645	7.721235	C	10.918263	8.25427	7.955633
C	9.41237	6.050513	4.804897	H	11.878566	8.251253	8.464221
H	8.383672	5.871721	4.499986	H	9.781942	8.576786	9.751536
H	9.939911	6.501367	3.952821	H	11.768134	7.969388	6.002926

H	9.88936	5.078015	4.98785	F	8.984739	8.924804	3.753004
C	7.275976	8.853277	8.549058	F	9.132802	6.732168	3.98649
H	6.330077	8.519956	8.122895	F	10.944201	7.960784	3.912541
H	7.279016	8.585216	9.613302	F	6.603239	9.804901	8.568541
H	7.297456	9.951495	8.506444	F	6.381015	7.619534	8.811965
H	11.864036	8.191949	8.13163	F	7.594773	8.77116	10.225183
C	12.02853	6.632081	5.937677	C	9.664224	7.926409	4.402914
H	12.02229	5.533599	5.930683	C	7.274388	8.657289	8.896379
H	12.130107	6.95196	4.892072				
H	12.920963	6.960126	6.479968				
C	9.936602	9.368503	9.606677				
H	9.51169	10.373033	9.478051				
H	9.435011	8.924498	10.477099				
H	10.997908	9.479423	9.850399				

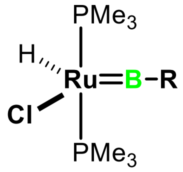

TM

**R** =	
m-Xylyl	**A'**
m-^{F}Xylyl	**B'**
2,6-dimethyl-4-*N*,*N*-dimethylaniline	**C'**
Duryl	**D'**
o-^{F}Xylyl	**E'**

**A'**		**NOIMAG**	**[-5760.97]**	**B'**		**NOIMAG**	**[-5810.88]**	**C'**		**NOIMAG**	**[-6752.92]**
C	-3.40661	-0.228344	0.975561	C	-3.397004	-0.127701	1.039272	C	-3.377797	-0.020547	1.130035
C	-5.923398	-0.108939	1.288939	C	-2.123807	-0.56893	1.450212	C	-2.100697	-0.60291	1.369951
C	-2.115259	-0.607989	1.402072	C	-4.532762	-0.427033	1.797831	C	-4.456823	-0.332301	1.950954
C	-4.538144	-0.514767	1.747197	C	-2.023152	-1.317318	2.641352	C	-1.959417	-1.510062	2.456739

C	-1.979791	-1.289147	2.629838	C	-4.424037	-1.170089	2.976518	C	-4.321974	-1.221947	3.048736
C	-4.362477	-1.192158	2.965132	C	-3.163082	-1.613154	3.392119	C	-3.051322	-1.807571	3.269805
C	-3.096178	-1.5884	3.421112	H	-0.429205	0.911889	-1.220425	H	-0.342761	0.928376	-1.353545
C	-2.946267	-2.343115	4.725439	B	-0.882264	-0.241987	0.613044	B	-0.904837	-0.258513	0.505379
H	-0.410007	0.894126	-1.275015	P	0.318526	-1.815897	-1.670201	P	0.374752	-1.809002	-1.727717
H	-6.552733	-0.990254	1.107521	P	1.115129	2.01575	0.924322	P	1.007855	1.982606	0.932806
H	-5.880986	0.471502	0.361603	Cl	2.910946	-0.696064	0.128884	Cl	2.846759	-0.773549	0.317317
H	-6.429853	0.49952	2.048953	Ru	0.621118	0.080518	-0.314621	Ru	0.633468	0.081981	-0.377405
H	-5.237306	-1.414013	3.577045	C	1.472323	-1.867379	-3.109283	C	1.636799	-1.945792	-3.070844
H	-3.139183	-3.415139	4.580629	H	2.495202	-1.810746	-2.725287	H	2.626816	-1.922804	-2.605105
H	-3.655801	-1.982691	5.479189	H	1.3402	-2.786687	-3.691895	H	1.514477	-2.871727	-3.645519
H	-1.933245	-2.242304	5.129286	H	1.289067	-0.999638	-3.750998	H	1.545244	-1.085419	-3.741682
B	-0.887791	-0.275359	0.558126	C	-1.333447	-2.076639	-2.448225	C	-1.219148	-2.029069	-2.633274
P	0.366023	-1.826323	-1.69962	H	-1.346713	-2.973766	-3.078109	H	-1.222655	-2.944265	-3.237383
P	1.03676	1.983349	0.942224	H	-2.0858	-2.176212	-1.659171	H	-2.032516	-2.070756	-1.901523
Cl	2.914402	-0.69737	0.178656	H	-1.583844	-1.199032	-3.051813	H	-1.382258	-1.160268	-3.278179
Ru	0.630114	0.070348	-0.348651	C	0.637708	-3.427146	-0.833621	C	0.557249	-3.424212	-0.853371
C	1.56832	-1.91588	-3.098808	H	-0.101351	-3.567887	-0.038356	H	-0.263965	-3.530933	-0.138101
H	2.577589	-1.860485	-2.679798	H	0.581558	-4.265536	-1.537738	H	0.546445	-4.268609	-1.552735
H	1.44925	-2.84395	-3.670594	H	1.632306	-3.380223	-0.380608	H	1.501002	-3.402023	-0.300513
H	1.416094	-1.057528	-3.761138	C	-0.222711	3.26264	1.16355	C	-0.331829	3.247279	1.061975
C	-1.259992	-2.087357	-2.53292	H	-0.586329	3.58495	0.183194	H	-0.598027	3.586889	0.056533
H	-1.26149	-2.996819	-3.145452	H	-1.053669	2.792903	1.699617	H	-1.211981	2.777034	1.511788
H	-2.03943	-2.1606	-1.767644	H	0.131074	4.131808	1.730261	H	-0.024904	4.104122	1.673536
H	-1.478204	-1.218669	-3.161564	C	2.488096	3.008391	0.193197	C	2.468356	2.985602	0.407535
C	0.639134	-3.432203	-0.834173	H	2.728819	3.875677	0.819221	H	2.642533	3.829005	1.086452
H	-0.12619	-3.550831	-0.060469	H	3.363336	2.358569	0.100339	H	3.34237	2.327291	0.396206
H	0.595337	-4.280094	-1.528068	H	2.195791	3.349365	-0.805147	H	2.300701	3.362921	-0.606532
H	1.619527	-3.389503	-0.350498	C	1.704487	1.708834	2.643742	C	1.383108	1.62936	2.70484
C	-0.322151	3.217856	1.129505	H	2.541644	1.006554	2.592405	H	2.218779	0.924057	2.73643
H	-0.619662	3.569163	0.136769	H	2.018803	2.637346	3.134552	H	1.635956	2.541894	3.257762
H	-1.184094	2.724711	1.590242	H	0.896354	1.24702	3.220052	H	0.508012	1.154837	3.160422
H	-0.014354	4.070438	1.746478	C	-5.889432	0.024371	1.302989	H	0.208472	-1.617116	2.324118
C	2.448166	3.008617	0.336052	C	-3.029448	-2.372189	4.694424	H	-2.907811	-2.50606	4.087397
H	2.640721	3.858441	1.001762	F	-5.854544	1.304259	0.814305	H	-3.114218	0.582535	-0.936119
H	3.332404	2.366435	0.281747	F	-6.339479	-0.774135	0.277303	H	-5.416679	0.124032	1.734547
H	2.220456	3.377475	-0.669316	F	-6.843787	-0.010181	2.2804	N	-5.395538	-1.500495	3.880704
C	1.513003	1.640109	2.691242	F	-2.78481	-1.520211	5.746639	C	-6.729756	-1.011169	3.538235

H	2.359189	0.946798	2.67854	F	-1.996698	-3.268743	4.666009	C	-5.287895	-2.570252	4.871593
H	1.783312	2.558183	3.226393	F	-4.164263	-3.069354	5.008207	H	-6.735529	0.082432	3.454489
H	0.671739	1.155315	3.19699	H	-1.05055	-1.669499	2.972457	H	-7.100272	-1.429874	2.587977
H	-3.519499	0.302861	0.032288	H	-3.493399	0.452177	0.125634	H	-7.424295	-1.290099	4.3332
H	-0.987505	-1.583328	2.967117	H	-5.306925	-1.402105	3.561624	H	-4.473108	-2.370829	5.578398
								H	-6.219062	-2.623281	5.439138
								H	-5.105234	-3.553013	4.406841
								C	-3.567029	0.957168	-0.01189
								H	-4.628651	1.157164	-0.19143
								H	-3.073612	1.912959	0.206287
								C	-0.632501	-2.192033	2.724689
								H	-0.477252	-2.350438	3.797881
								H	-0.602313	-3.178384	2.241668
**D'**		**NOIMAG**	**[-6500.54]**	**E'**		**NOIMAG**	**[-5808.48]**	**H_2**		**NOIMAG**	**[-153.64]**
C	-3.644385	0.529392	-0.334035	C	-3.518128	-0.687018	0.693668	H	0	0	0.373105
C	-3.416906	-0.231061	0.955311	C	-2.214315	-1.038896	1.134205	H	0	0	-0.373105
C	-5.929501	-0.248028	1.357	C	-4.663256	-1.207079	1.303839				
C	-2.103789	-0.585067	1.364712	C	-2.13664	-1.94478	2.22579				
C	-4.512996	-0.598623	1.758755	C	-4.546898	-2.094362	2.373026				
C	-1.884083	-1.306868	2.570063	C	-3.283273	-2.462676	2.833633				
C	-4.272628	-1.300765	2.946356	H	-0.392084	0.901474	-1.163931				
C	-0.485126	-1.697546	2.996178	B	-0.939949	-0.471261	0.467822				
C	-2.987499	-1.661912	3.369794	P	0.793738	-1.599101	-1.815734				
C	-2.800813	-2.421927	4.665173	P	0.755395	1.994419	1.196568				
H	-4.268447	-0.048135	-1.030148	Cl	2.992998	-0.317721	0.332991				
H	-0.304341	0.978873	-1.372514	Ru	0.623629	0.109249	-0.20684				
H	-2.699858	0.759106	-0.833784	C	2.000684	-1.145509	-3.137095				
H	-4.173846	1.47412	-0.150357	H	2.963107	-0.937581	-2.660524				
H	-6.192494	-0.685845	0.384581	H	2.110629	-1.955836	-3.867537				
H	-6.064643	0.837555	1.25948	H	1.656009	-0.23953	-3.645549				
H	-6.648359	-0.613045	2.097385	C	-0.704383	-2.078015	-2.776462				
H	0.26511	-1.385218	2.264416	H	-0.465465	-2.834212	-3.533346				
H	-0.399842	-2.784898	3.124436	H	-1.45825	-2.474704	-2.089887				
H	-5.123611	-1.580382	3.567571	H	-1.118875	-1.188707	-3.259113				
H	-0.223717	-1.248354	3.964095	C	1.436133	-3.221541	-1.223843				
H	-2.316492	-3.393082	4.496554	H	0.707688	-3.668431	-0.541737				
H	-3.762633	-2.603753	5.155142	H	1.623568	-3.903402	-2.061651				

H	-2.160866	-1.870217	5.366721	H	2.362132	-3.036882	-0.671887
B	-0.900673	-0.199308	0.507269	C	-0.762656	2.996252	1.494589
P	0.307994	-1.801542	-1.679861	H	-1.179056	3.309552	0.533328
P	1.068541	2.030705	0.900785	H	-1.50727	2.375065	2.001401
Cl	2.812162	-0.801392	0.347272	H	-0.542617	3.877072	2.109052
Ru	0.635963	0.115566	-0.376285	C	1.94287	3.238171	0.524318
C	1.579529	-2.058267	-2.994502	H	2.034078	4.100602	1.195272
H	2.563021	-2.06572	-2.514851	H	2.915059	2.750453	0.407498
H	1.417018	-3.00021	-3.531839	H	1.596634	3.574415	-0.458062
H	1.540993	-1.223857	-3.702309	C	1.39623	1.708416	2.900476
C	-1.283208	-1.970421	-2.600224	H	2.330924	1.146235	2.820049
H	-1.322902	-2.902034	-3.177066	H	1.567733	2.656659	3.42338
H	-2.107414	-1.951325	-1.880183	H	0.674592	1.106022	3.458875
H	-1.396521	-1.114599	-3.272543	H	-5.643389	-0.916816	0.941525
C	0.38871	-3.380261	-0.728692	H	-3.185689	-3.152231	3.665056
H	-0.430387	-3.39479	-0.002557	F	-0.890904	-3.248151	3.811458
H	0.314221	-4.255611	-1.384687	F	-0.049623	-1.30939	3.218786
H	1.337424	-3.395201	-0.184103	F	-0.026871	-3.004209	1.808498
C	-0.23261	3.337258	0.978091	F	-3.121405	-0.18704	-1.618136
H	-0.448672	3.681789	-0.037731	F	-3.150664	1.505573	-0.204862
H	-1.14565	2.89639	1.391102	F	-5.02053	0.497962	-0.757513
H	0.07811	4.186006	1.59891	H	-5.438314	-2.497564	2.845927
C	2.570446	2.965833	0.370154	C	-3.698177	0.278202	-0.466991
H	2.765621	3.819907	1.029562	C	-0.779975	-2.373569	2.761994
H	3.420105	2.276504	0.389156				
H	2.430911	3.321961	-0.655695				
C	1.413282	1.703811	2.68363				
H	2.213675	0.9602	2.740155				
H	1.706639	2.617191	3.214355				
H	0.514213	1.284386	3.14559				

References

[1] V. K. Issleib, A. Brack, *Z. anorg. allg. Chem.* **1954**, *277*, 258-270.

[2] E. Zysman-Colman, K. Arias, J. S. Siegel, *Can. J. Chem.* **2009**, *87*, 440-447.

[3] A. Castelló-Micó, S. A. Herbert, T. León, T. Bein, P. Knochel, *Angew. Chem. Int. Ed.* **2016**, *55*, 401–404.

[4] N. Arnold, S. Mozo, U. Paul, U. Radius, H. Braunschweig, *Organometallics*, **2015**, *34*, 5709-5715.

[5] K. Samigullin, M. Bolte, H.-W. Lerner, M. Wagner, *Organometallics*, **2014**, *33*, 3564-3569.

[6] a) L. Porri, M. C: Gallzzi, A. Colombo, G. Allegra, *Tetrahedron Lett.* **1965**, *47*, 4187-4189; b) D. N. Cox, R. Roulet, *Inorg. Chem.* **1990**, *29*, 1360-1365.

[7] H. Werner, W. Stüer, S. Jung, B. Weberndörfer, J. Wolf, *Eur. J. Inorg. Chem.* **2002**, 1076-1080

[8] H. L. M. van Gaal, F. L. A. Van den Bekerom, *J. Organomet. Chem.* **1977**, *134*, 237-248.

[9] a) L. J. Sewell, A. B. Chaplin, J. A. B. Abdalla, A. S. Weller, *Dalton Trans.* **2010**, *39*, 7437-7439; b) B. R. James, M. Preece, S. D. Robinson, *Inorg. Chim. Acta* **1979**, *34*, L219-L221.

[10] S. Brinkmann, R. H. Morris, R. Ramachandran, S.-H. Park, R. H. Crabtree, B. P. Patel, B. J. Pistorio, *Inorganic Synthesis*, Vol. 32, John Wiley & Sons, Inc., Hoboken, NJ, USA, **1990**.

[11] G. Sheldrick, *Acta Cryst.* **2015**, *A71*, 3–8.

[12] G. Sheldrick, *Acta Cryst.* **2008**, *A64*, 112–122.

[13] a) E. J. Baerends, T. Ziegler, A. J. Atkins, J. Autschbach, O. Baseggio, D. Bashford, A. Bérces, F. M. Bickelhaupt, C. Bo, P. M. Boerrigter, L. Cavallo, C. Daul, D. P. Chong, D. V. Chulhai, L. Deng, R. M. Dickson, J. M. Dieterich, D. E. Ellis, M. van Faassen, L. Fan, T. H. Fischer, C. Fonseca Guerra, M. Franchini, A. Ghysels, A. Giammona, S. J. A. van Gisbergen, A. Goez, A. W. Götz, J. A. Groeneveld, O. V. Gritsenko, M. Grüning, S. Gusarov, F. E. Harris, P. van den Hoek, Z. Hu, C. R. Jacob, H. Jacobsen, L. Jensen, L. Joubert, J. W. Kaminski, G. van Kessel, C. König, F. Kootstra, A. Kovalenko, M. V. Krykunov, E. van Lenthe, D. A. McCormack, A. Michalak, M. Mitoraj, S. M. Morton, J. Neugebauer, V. P. Nicu, L. Noodleman, V. P. Osinga, S. Patchkovskii, M. Pavanello, C. A. Peeples, P. H. T. Philipsen, D. Post, C. C. Pye, H. Ramanantoanina, P. Ramos, W. Ravenek, J. I. Rodríguez, P. Ros, R. Rüger, P. R. T. Schipper, D. Schlüns, H. van Schoot, G. Schreckenbach, J. S. Seldenthuis, M. Seth, J. G. Snijders, M. Solà, M. Stener, M. Swart, D. Swerhone, V. Tognetti, G. te Velde, P. Vernooijs, L. Versluis, L. Visscher, O. Visser, F. Wang, T. A. Wesolowski, E. M. van Wezenbeek, G. Wiesenekker, S. K. Wolff, T. K. Woo, A. L. Yakovlev, ADF2016, SCM, Theoretical Chemistry, Vrije Universiteit, http://www.scm.com, Amsterdam, The Netherlands, **2016**; b) C. Fonseca Guerra, J. G. Snijders, G. te Velde, E. J. Baerends, *Theor. Chem. Acc.* **1998**, *99*, 391-403; c) G. te Velde, F. M. Bickelhaupt, E. J. Baerends, C. Fonseca Guerra, S. J. A. van Gisbergen, J. G. Snijders, T. Ziegler, *J. Comput. Chem.* **2001**, *22*, 931-967.

[14] a) A. D. Becke, *J. Chem. Phys.* **1988**, *88*, 2547-2553; b.) M. Franchini, P. H. T. Philipsen, L. Visscher, *J. Comput. Chem.* **2013**, *34*, 1819-1827.

[15] E. Van Lenthe, E. J. Baerends, *J. Comput. Chem.* **2003**, *24*, 1142-1156.

[16] J. C. Slater, *Quantum Theory of Molecules and Solids*, 4 ed., McGraw-Hill, New York, **1974**.

[17] a) A. D. Becke, *J. Chem. Phys.* **1986**, *84*, 4524; b) A. D. Becke, *Phys. Rev. A* **1988**, *38*, 3098-3100.

[18] a) T. V. Russo, R. L. Martin, P. J. Hay, *J. Chem. Phys.* **1994**, *101*, 7729-7737; b) B. G. Johnson, P. M. W. Gill, J. A. Pople, *J. Chem. Phys.* **1993**, *98*, 5612-5626; c) C. Lee, W. Yang, R. G. Parr, *Phys. Rev. B* **1988**, *37*, 785-789.

[19] a) E. v. Lenthe, E. J. Baerends, J. G. Snijders, *J. Chem. Phys.* **1993**, *99*, 4597-4610; b) E. van Lenthe, E. J. Baerends, J. G. Snijders, *J. Chem. Phys.* **1994**, *101*, 9783-9792; c) E. van Lenthe, J. G. Snijders, E. J. Baerends, *J. Chem. Phys.* **1996**, *105*, 6505-6516.

[20] S. Grimme, S. Ehrlich, L. Goerigk, *J. Comput. Chem.* **2011**, *32*, 1456-1465.

[21] a) A. Berces, R. M. Dickson, L. Y. Fan, H. Jacobsen, D. Swerhone, T. Ziegler, *Comput. Phys. Commun.* **1997**, *100*, 247-262; b) H. Jacobsen, A. Berces, D. P. Swerhone, T. Ziegler, *Comput. Phys. Commun.* **1997**, *100*, 263-276; c) S. K. Wolff, *Int. J. Quantum Chem.* **2005**, *104*, 645-659.

[22] a) M. Swart, F. M. Bickelhaupt, *Journal of Chemical Theory and Computation* **2006**, *2*, 281-287; b) M. Swart, E. Rösler, F. M. Bickelhaupt, *J. Comput. Chem.* **2006**, *27*, 1486-1493.
[23] F. Jensen, *Introduction to Computational Chemistry*, 2. ed., Wiley, Chichester, **2007**.
